# Supplementary material for: Food and feed safety of the Bacillus thuringiensis derived protein Vpb4Da2, a novel protein for control of western corn rootworm
Source: PLoS One. 2022 Aug 3;17(8):e0272311. doi: 10.1371/journal.pone.0272311 (PMC9348738; doi:10.1371/journal.pone.0272311)
Supplement: S1 Raw images — (PDF) [file pone.0272311.s001.pdf]

Fig1 (supplement)

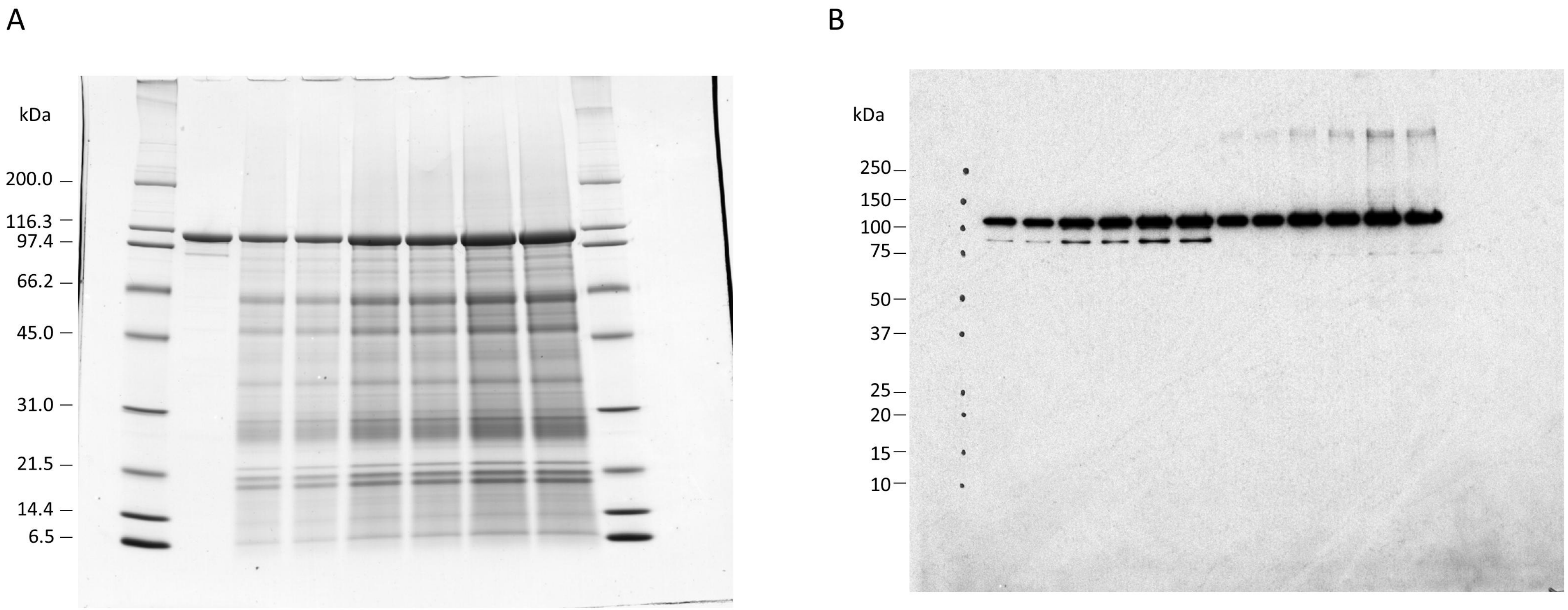

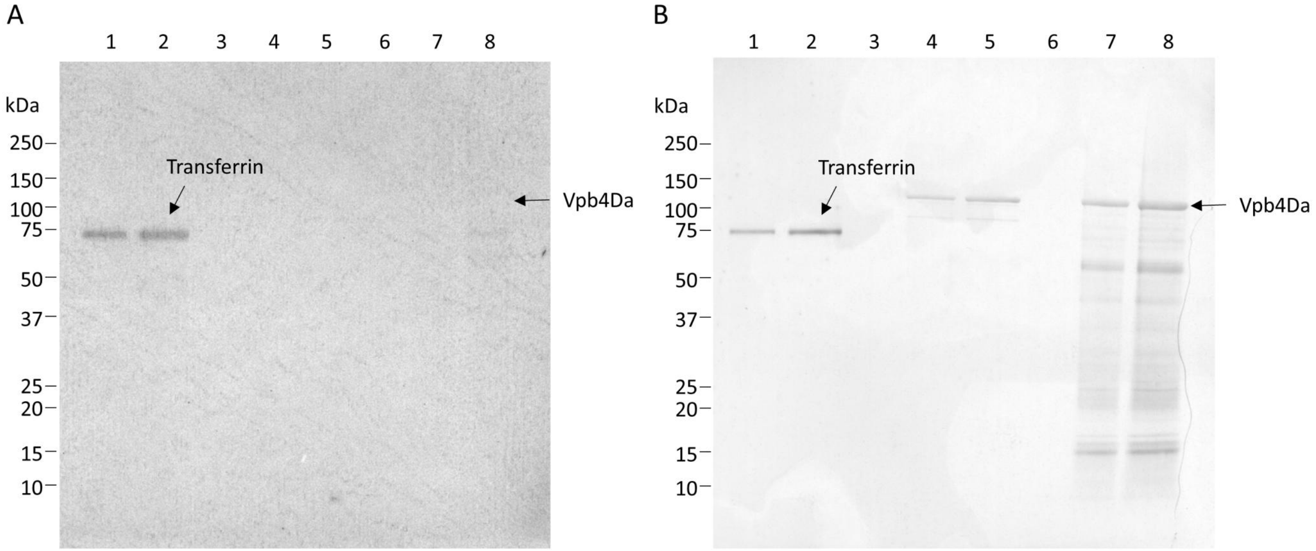
A
1 2 3 4 5 6 7 8
kDa
250
150
100
75
50
37
25
20
15
10
Transferrin
Vpb4Da
B
1 2 3 4 5 6 7 8
kDa
250
150
100
75
50
37
25
20
15
10
Transferrin
Vpb4Da

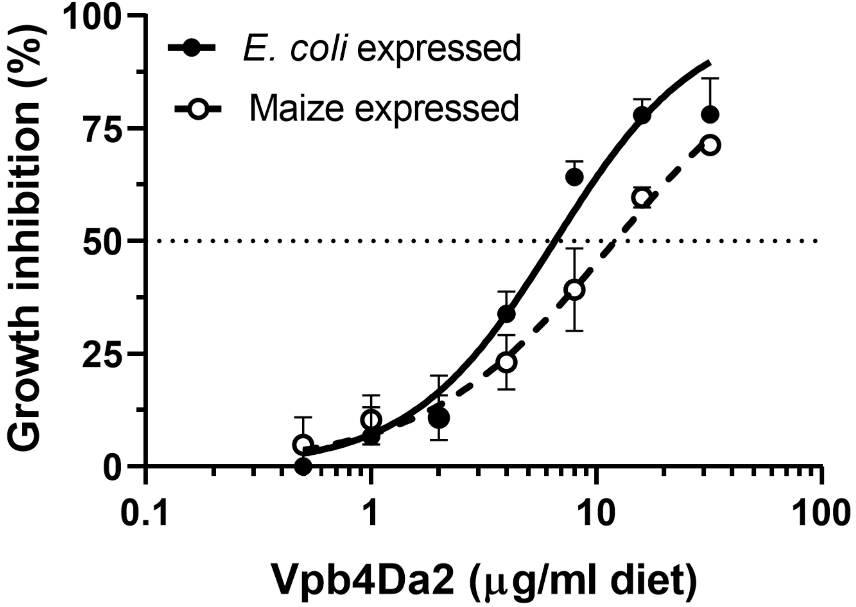

Vpb4Da2 Protein	Mean EC_{50} (ug/ml diet)	Standard Error	N
E. Coli expressed	6.1	0.25	3
Maize expressed	12.3	0.88	3

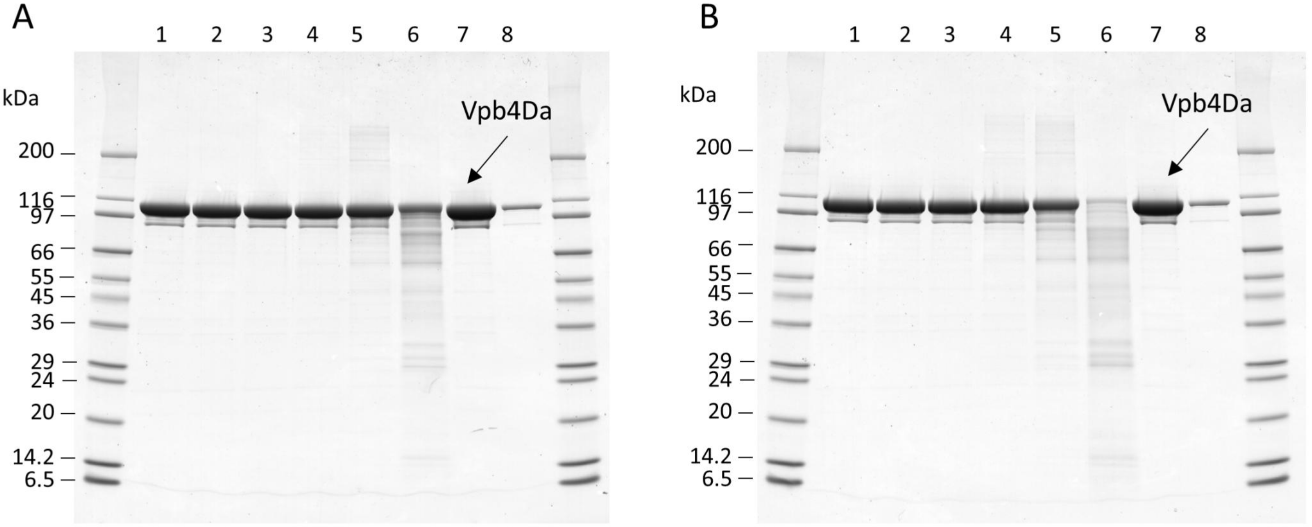
A
B
1 2 3 4 5 6 7 8
kDa
Vpb4Da
200
116
97
66
55
45
36
29
24
20
14.2
6.5

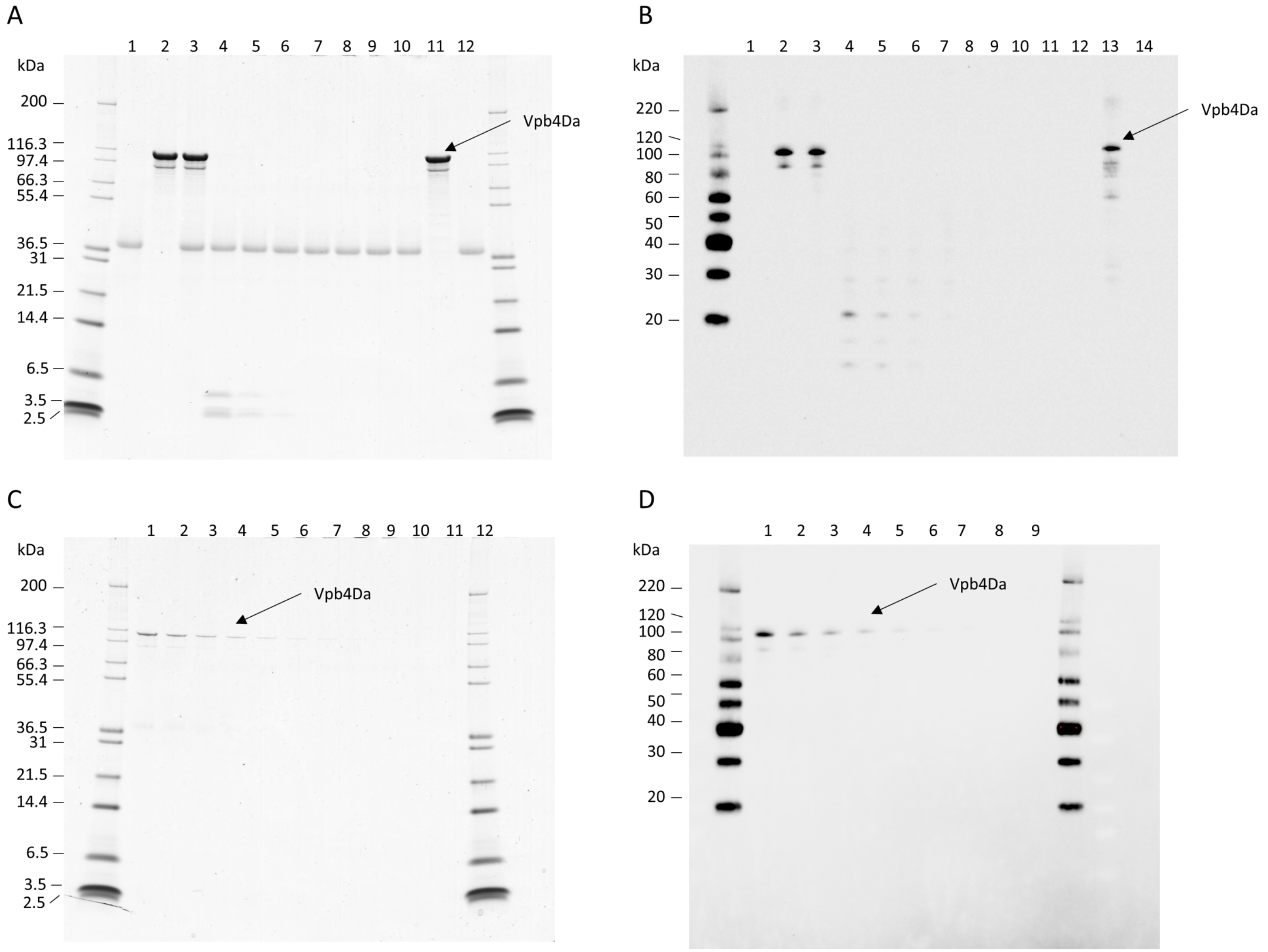
A
B
C
D
kDa
Vpb4Da
1 2 3 4 5 6 7 8 9 10 11 12
1 2 3 4 5 6 7 8 9 10 11 12 13 14
1 2 3 4 5 6 7 8 9
200
116.3
97.4
66.3
55.4
36.5
31
21.5
14.4
6.5
3.5
2.5
220
120
100
80
60
50
40
30
20
