# Supplementary material for: Food and feed safety of the Bacillus thuringiensis derived protein Vpb4Da2, a novel protein for control of western corn rootworm
Source: PLoS One. 2022 Aug 3;17(8):e0272311. doi: 10.1371/journal.pone.0272311 (PMC9348738; doi:10.1371/journal.pone.0272311)
Supplement: S1 File — (PDF) [file pone.0272311.s002.pdf]

MATRIX SCIENCE MASCOT Search Results

Protein View: TIC5290

TIC5290

**Database:**	TIC5290b
**Score:**	49738
**Nominal mass (M_r):**	104955
**Calculated pI:**	6.32

Sequence similarity is available as **an NCBI BLAST search of TIC5290 against nr**.

Search parameters

**MS data file:**	C:\Users\EKXMP\Desktop\20210216 Vpb\20210212Vpb(1).raw
**Enzyme:**	Trypsin: cuts C-term side of KR unless next residue is P.
**Fixed modifications:**	Carbamidomethyl (C)
**Variable modifications:**	Acetyl (Protein N-term), Oxidation (M)

Protein sequence coverage: 97%

Matched peptides shown in ***bold red***.

```
  1 MQNIVSSKSE QATVIGLVGF YFKDSTFKEL MFIQVGEKSN LMNKARINTD
 51 AQQIQSIRWM GNLKSPQTGE YRLSTSSDEN VILQINGETV INQASIQKNL
101 KLEANQVYEI KIEYRNTSNT LPDLQLFWSM NNAQKEQIPE KYILSPNFSE
151 KANSLAEKET QSFFPNYNLF DRQQENGEKQ SMSTPVDTDN DCIPDEWEEK
201 GYTFRNQQIV PWNDAYSAEG YKKYVSNPYH ARTVKDPYTD FEKVTGHMPA
251 ATKYEARDPL VAAYPSVGVG MEKLHFSKND TVTEGNADTK SKTTTKTDTT
301 TNTVEIGGSL GFSDKGFSFS ISPKYTHSWS SSTSVADTDS TTWSSQIGIN
351 TAERAYLNAN VRYYNGGTAP IYDLKPTTNF VFQNSGDSIT TITAGPNQIG
401 NSLGAGDTYP QKGQAPISLD KANEAGTVKI AINAEQLDKI QAGTEILNIE
451 TTQNRGQYGI LDEKGQVIPG GEWDPIRTNI DAVSGSLTLN LGTGKDSLER
501 RVAAKNMNDP EDKTPEITIK EAIKKAFNAQ EKDGRLYYTD QGEKDIFIDE
551 PSINLITDEN TKKEIERQLN QMPGKTVYDV KWKRGMKITL HVPIKYYDFE
601 TSENLWYYTY QESGGYTGKK RGRIGTDGHG TAMSNPQLKP YTSYTVRAYV
651 RTASTTGSNE VVFYADNSSG NGQGAKVSGK VTGGKWKIAE FSFNTFNNPE
701 YFKIIGLKNN GNANLHFDDV SVIEWKTNEN LQKKHIFEKW SFGSNDEMVI
751 GATFTRVPSS KIRYQWKING RLGSIIPAPP LDANGKRTVT YGSITAITPM
801 ELYAVDEKND NLKVKVAELG ESEIEKVMID AHKFSGWWYL SENPNLYSGL
851 SLYKLPDIFY NNVSSYKIRV NGKKVQTVSK PSPFLFQITF NLKNPNGGTY
901 PTKDASVELW ATVGGKDLKV LHKWIQKSDV MYSQTNN
```

Unformatted sequence string: **937 residues** (for pasting into other applications).

Sort peptides by ◉ Residue Number ○ Increasing Mass ○ Decreasing Mass

Show predicted peptides also

Query	Start - End	Observed	Mr(expt)	Mr(calc)	ppm	M	Score	Expect	Rank	U	Peptide
1575	1 - 8	474.7450	947.4754	947.4746	0.89	0	43	5.1e-005	1	U	-.MQNIVSSK.S + Acetyl (Protein N-term)
1670	1 - 8	482.7416	963.4687	963.4695	-0.82	0	19	0.013	1	U	-.MQNIVSSK.S + Acetyl (Protein N-term); Oxidation (M)
1671	1 - 8	482.7420	963.4695	963.4695	-0.059	0	32	0.00066	1	U	-.MQNIVSSK.S + Acetyl (Protein N-term); Oxidation (M)
1672	1 - 8	482.7421	963.4696	963.4695	0.13	0	34	0.00038	1	U	-.MQNIVSSK.S + Acetyl (Protein N-term); Oxidation (M)
1673	1 - 8	482.7422	963.4698	963.4695	0.32	0	22	0.0058	1	U	-.MQNIVSSK.S + Acetyl (Protein N-term); Oxidation (M)
1674	1 - 8	482.7422	963.4699	963.4695	0.39	0	0	1	1	U	-.MQNIVSSK.S + Acetyl (Protein N-term); Oxidation (M)
1675	1 - 8	482.7424	963.4703	963.4695	0.83	0	26	0.0024	1	U	-.MQNIVSSK.S + Acetyl (Protein N-term); Oxidation (M)
11194	1 - 23	868.7844	2603.3313	2603.3305	0.28	1	35	0.00029	1	U	-.MQNIVSSKSEQATVIGLVGFYFK.D + Acetyl (Protein N-term); Oxidation (M)
11195	1 - 23	868.7844	2603.3314	2603.3305	0.35	1	38	0.00017	1	U	-.MQNIVSSKSEQATVIGLVGFYFK.D + Acetyl (Protein N-term); Oxidation (M)
667	2 - 8	388.2186	774.4226	774.4236	-1.18	0	3	0.55	1	U	M.QNIVSSK.S
668	2 - 8	388.2190	774.4234	774.4236	-0.23	0	6	0.26	1	U	M.QNIVSSK.S
5485	9 - 23	553.6285	1657.8638	1657.8716	-4.69	0	21	0.008	1	U	K.SEQATVIGLVGFYFK.D
5486	9 - 23	829.9420	1657.8695	1657.8716	-1.26	0	88	1.8e-009	1	U	K.SEQATVIGLVGFYFK.D
5487	9 - 23	553.6307	1657.8702	1657.8716	-0.82	0	59	1.4e-006	1	U	K.SEQATVIGLVGFYFK.D
5488	9 - 23	553.6307	1657.8704	1657.8716	-0.71	0	42	6.7e-005	1	U	K.SEQATVIGLVGFYFK.D
5489	9 - 23	829.9425	1657.8705	1657.8716	-0.67	0	75	3.3e-008	1	U	K.SEQATVIGLVGFYFK.D
5490	9 - 23	553.6308	1657.8706	1657.8716	-0.60	0	35	0.00034	1	U	K.SEQATVIGLVGFYFK.D
5491	9 - 23	829.9426	1657.8707	1657.8716	-0.52	0	87	1.9e-009	1	U	K.SEQATVIGLVGFYFK.D
5492	9 - 23	553.6309	1657.8709	1657.8716	-0.38	0	32	0.00058	1	U	K.SEQATVIGLVGFYFK.D
5493	9 - 23	553.6309	1657.8709	1657.8716	-0.38	0	51	7.1e-006	1	U	K.SEQATVIGLVGFYFK.D
5494	9 - 23	829.9427	1657.8709	1657.8716	-0.37	0	76	2.7e-008	1	U	K.SEQATVIGLVGFYFK.D
5495	9 - 23	553.6310	1657.8711	1657.8716	-0.27	0	19	0.011	1	U	K.SEQATVIGLVGFYFK.D
5496	9 - 23	829.9429	1657.8712	1657.8716	-0.23	0	74	3.8e-008	1	U	K.SEQATVIGLVGFYFK.D

Query	Start - End	Observed	Mr(expt)	Mr(calc)	ppm	M	Score	Expect	Rank	U	Peptide
5497	9 - 23	553.6310	1657.8713	1657.8716	-0.16	0	44	4e-005	1	U	K.SEQATVIGLVGFYFK.D
5498	9 - 23	829.9429	1657.8713	1657.8716	-0.15	0	75	3.5e-008	1	U	K.SEQATVIGLVGFYFK.D
5499	9 - 23	829.9429	1657.8713	1657.8716	-0.15	0	88	1.7e-009	1	U	K.SEQATVIGLVGFYFK.D
5500	9 - 23	829.9430	1657.8714	1657.8716	-0.080	0	80	1e-008	1	U	K.SEQATVIGLVGFYFK.D
5501	9 - 23	553.6311	1657.8715	1657.8716	-0.050	0	54	4e-006	1	U	K.SEQATVIGLVGFYFK.D
5502	9 - 23	829.9431	1657.8716	1657.8716	-0.0066	0	79	1.2e-008	1	U	K.SEQATVIGLVGFYFK.D
5503	9 - 23	829.9431	1657.8716	1657.8716	-0.0066	0	78	1.5e-008	1	U	K.SEQATVIGLVGFYFK.D
5504	9 - 23	553.6312	1657.8719	1657.8716	0.17	0	41	7.3e-005	1	U	K.SEQATVIGLVGFYFK.D
5505	9 - 23	553.6312	1657.8719	1657.8716	0.17	0	48	1.7e-005	1	U	K.SEQATVIGLVGFYFK.D
5506	9 - 23	553.6312	1657.8719	1657.8716	0.17	0	44	4.1e-005	1	U	K.SEQATVIGLVGFYFK.D
5507	9 - 23	553.6312	1657.8719	1657.8716	0.17	0	61	8.8e-007	1	U	K.SEQATVIGLVGFYFK.D
5508	9 - 23	829.9432	1657.8719	1657.8716	0.21	0	83	4.9e-009	1	U	K.SEQATVIGLVGFYFK.D
5509	9 - 23	829.9432	1657.8719	1657.8716	0.21	0	71	8e-008	1	U	K.SEQATVIGLVGFYFK.D
5510	9 - 23	829.9432	1657.8719	1657.8716	0.21	0	87	1.8e-009	1	U	K.SEQATVIGLVGFYFK.D
5511	9 - 23	553.6313	1657.8720	1657.8716	0.28	0	36	0.00025	1	U	K.SEQATVIGLVGFYFK.D
5512	9 - 23	829.9434	1657.8722	1657.8716	0.36	0	79	1.3e-008	1	U	K.SEQATVIGLVGFYFK.D
5513	9 - 23	829.9434	1657.8722	1657.8716	0.36	0	83	4.7e-009	1	U	K.SEQATVIGLVGFYFK.D
5514	9 - 23	553.6313	1657.8722	1657.8716	0.39	0	59	1.4e-006	1	U	K.SEQATVIGLVGFYFK.D
5515	9 - 23	553.6313	1657.8722	1657.8716	0.39	0	40	0.0001	1	U	K.SEQATVIGLVGFYFK.D
5516	9 - 23	553.6313	1657.8722	1657.8716	0.39	0	62	6.1e-007	1	U	K.SEQATVIGLVGFYFK.D
5517	9 - 23	829.9434	1657.8723	1657.8716	0.43	0	80	9.7e-009	1	U	K.SEQATVIGLVGFYFK.D
5518	9 - 23	553.6314	1657.8724	1657.8716	0.50	0	42	5.7e-005	1	U	K.SEQATVIGLVGFYFK.D
5519	9 - 23	553.6314	1657.8724	1657.8716	0.50	0	58	1.6e-006	1	U	K.SEQATVIGLVGFYFK.D
5520	9 - 23	829.9435	1657.8724	1657.8716	0.51	0	68	1.8e-007	1	U	K.SEQATVIGLVGFYFK.D
5521	9 - 23	829.9435	1657.8725	1657.8716	0.58	0	69	1.3e-007	1	U	K.SEQATVIGLVGFYFK.D
5522	9 - 23	553.6315	1657.8726	1657.8716	0.61	0	34	0.0004	1	U	K.SEQATVIGLVGFYFK.D
5523	9 - 23	553.6315	1657.8726	1657.8716	0.61	0	39	0.00012	1	U	K.SEQATVIGLVGFYFK.D
5524	9 - 23	553.6315	1657.8728	1657.8716	0.72	0	32	0.00071	1	U	K.SEQATVIGLVGFYFK.D
5525	9 - 23	829.9437	1657.8728	1657.8716	0.73	0	77	2e-008	1	U	K.SEQATVIGLVGFYFK.D
5526	9 - 23	829.9437	1657.8729	1657.8716	0.80	0	74	3.9e-008	1	U	K.SEQATVIGLVGFYFK.D
5527	9 - 23	829.9437	1657.8729	1657.8716	0.80	0	79	1.3e-008	1	U	K.SEQATVIGLVGFYFK.D
5528	9 - 23	553.6316	1657.8729	1657.8716	0.83	0	54	3.7e-006	1	U	K.SEQATVIGLVGFYFK.D
5529	9 - 23	553.6316	1657.8729	1657.8716	0.83	0	60	1e-006	1	U	K.SEQATVIGLVGFYFK.D
5530	9 - 23	553.6316	1657.8729	1657.8716	0.83	0	55	3.4e-006	1	U	K.SEQATVIGLVGFYFK.D
5531	9 - 23	829.9438	1657.8730	1657.8716	0.88	0	83	4.6e-009	1	U	K.SEQATVIGLVGFYFK.D
5532	9 - 23	829.9438	1657.8731	1657.8716	0.95	0	92	6.4e-010	1	U	K.SEQATVIGLVGFYFK.D
5533	9 - 23	829.9439	1657.8733	1657.8716	1.02	0	84	4e-009	1	U	K.SEQATVIGLVGFYFK.D
5534	9 - 23	829.9439	1657.8733	1657.8716	1.02	0	89	1.2e-009	1	U	K.SEQATVIGLVGFYFK.D
5535	9 - 23	829.9440	1657.8734	1657.8716	1.10	0	83	4.8e-009	1	U	K.SEQATVIGLVGFYFK.D
5536	9 - 23	829.9440	1657.8734	1657.8716	1.10	0	74	4.1e-008	1	U	K.SEQATVIGLVGFYFK.D
5537	9 - 23	553.6318	1657.8735	1657.8716	1.16	0	33	0.00047	1	U	K.SEQATVIGLVGFYFK.D
5538	9 - 23	553.6318	1657.8735	1657.8716	1.16	0	55	3.2e-006	1	U	K.SEQATVIGLVGFYFK.D
5539	9 - 23	829.9440	1657.8735	1657.8716	1.17	0	76	2.3e-008	1	U	K.SEQATVIGLVGFYFK.D
5540	9 - 23	829.9441	1657.8736	1657.8716	1.25	0	82	6.7e-009	1	U	K.SEQATVIGLVGFYFK.D
5541	9 - 23	553.6318	1657.8737	1657.8716	1.27	0	32	0.00057	1	U	K.SEQATVIGLVGFYFK.D
5542	9 - 23	829.9442	1657.8738	1657.8716	1.32	0	81	7.7e-009	1	U	K.SEQATVIGLVGFYFK.D
5543	9 - 23	553.6320	1657.8740	1657.8716	1.50	0	44	3.8e-005	1	U	K.SEQATVIGLVGFYFK.D
5544	9 - 23	829.9444	1657.8742	1657.8716	1.61	0	68	1.7e-007	1	U	K.SEQATVIGLVGFYFK.D
5545	9 - 23	829.9445	1657.8744	1657.8716	1.69	0	75	3.4e-008	1	U	K.SEQATVIGLVGFYFK.D
5546	9 - 23	829.9446	1657.8746	1657.8716	1.83	0	73	5.2e-008	1	U	K.SEQATVIGLVGFYFK.D
5547	9 - 23	553.6324	1657.8753	1657.8716	2.27	0	27	0.0018	1	U	K.SEQATVIGLVGFYFK.D
5548	9 - 23	553.6324	1657.8755	1657.8716	2.38	0	28	0.0016	1	U	K.SEQATVIGLVGFYFK.D
5549	9 - 23	553.6325	1657.8757	1657.8716	2.49	0	29	0.0013	1	U	K.SEQATVIGLVGFYFK.D
5550	9 - 23	829.9454	1657.8762	1657.8716	2.79	0	82	6e-009	1	U	K.SEQATVIGLVGFYFK.D
5551	9 - 23	829.9454	1657.8763	1657.8716	2.87	0	75	2.9e-008	1	U	K.SEQATVIGLVGFYFK.D
5552	9 - 23	829.9470	1657.8794	1657.8716	4.71	0	17	0.021	1	U	K.SEQATVIGLVGFYFK.D
9121	9 - 28	746.3892	2236.1458	2236.1416	1.89	1	18	0.015	1	U	K.SEQATVIGLVGFYFKDSTFK.E
192	24 - 28	299.1475	596.2805	596.2806	-0.17	0	12	0.065	1	U	K.DSTFK.E
193	24 - 28	299.1476	596.2806	596.2806	-0.070	0	6	0.22	1	U	K.DSTFK.E
194	24 - 28	299.1476	596.2806	596.2806	-0.070	0	9	0.12	1	U	K.DSTFK.E
6139	24 - 38	886.4508	1770.8871	1770.8862	0.48	1	74	3.6e-008	1	U	K.DSTFKELMFIQVGEK.S
6140	24 - 38	591.3032	1770.8878	1770.8862	0.92	1	39	0.00012	1	U	K.DSTFKELMFIQVGEK.S
6141	24 - 38	591.3035	1770.8886	1770.8862	1.33	1	45	3.5e-005	1	U	K.DSTFKELMFIQVGEK.S
6142	24 - 38	591.3035	1770.8888	1770.8862	1.43	1	36	0.00027	1	U	K.DSTFKELMFIQVGEK.S
6289	24 - 38	596.6315	1786.8728	1786.8811	-4.68	1	36	0.00023	1	U	K.DSTFKELMFIQVGEK.S + Oxidation (M)
6290	24 - 38	894.4464	1786.8781	1786.8811	-1.67	1	21	0.0087	1	U	K.DSTFKELMFIQVGEK.S + Oxidation (M)
6291	24 - 38	596.6342	1786.8806	1786.8811	-0.28	1	32	0.00062	1	U	K.DSTFKELMFIQVGEK.S + Oxidation (M)
6292	24 - 38	596.6345	1786.8816	1786.8811	0.23	1	28	0.0016	1	U	K.DSTFKELMFIQVGEK.S + Oxidation (M)
6293	24 - 38	596.6347	1786.8823	1786.8811	0.65	1	19	0.014	1	U	K.DSTFKELMFIQVGEK.S + Oxidation (M)
6294	24 - 38	596.6347	1786.8823	1786.8811	0.65	1	21	0.0084	1	U	K.DSTFKELMFIQVGEK.S + Oxidation (M)
6295	24 - 38	894.4485	1786.8825	1786.8811	0.79	1	36	0.00022	1	U	K.DSTFKELMFIQVGEK.S + Oxidation (M)
6296	24 - 38	894.4489	1786.8833	1786.8811	1.20	1	66	2.4e-007	1	U	K.DSTFKELMFIQVGEK.S + Oxidation (M)
3041	29 - 38	597.3134	1192.6123	1192.6162	-3.27	0	33	0.00046	1	U	K.ELMFIQVGEK.S
3042	29 - 38	597.3151	1192.6157	1192.6162	-0.40	0	35	0.00029	1	U	K.ELMFIQVGEK.S
3043	29 - 38	597.3154	1192.6163	1192.6162	0.11	0	17	0.022	1	U	K.ELMFIQVGEK.S
3044	29 - 38	597.3163	1192.6180	1192.6162	1.54	0	42	6.7e-005	1	U	K.ELMFIQVGEK.S
3244	29 - 38	605.3101	1208.6056	1208.6111	-4.58	0	39	0.00012	1	U	K.ELMFIQVGEK.S + Oxidation (M)
3245	29 - 38	605.3120	1208.6093	1208.6111	-1.45	0	51	8.5e-006	1	U	K.ELMFIQVGEK.S + Oxidation (M)
3246	29 - 38	605.3120	1208.6095	1208.6111	-1.35	0	38	0.00015	1	U	K.ELMFIQVGEK.S + Oxidation (M)
3247	29 - 38	605.3123	1208.6101	1208.6111	-0.84	0	25	0.003	1	U	K.ELMFIQVGEK.S + Oxidation (M)
3248	29 - 38	605.3125	1208.6104	1208.6111	-0.54	0	24	0.0042	1	U	K.ELMFIQVGEK.S + Oxidation (M)
3249	29 - 38	605.3126	1208.6106	1208.6111	-0.44	0	17	0.019	1	U	K.ELMFIQVGEK.S + Oxidation (M)
3250	29 - 38	605.3126	1208.6107	1208.6111	-0.34	0	22	0.0067	1	U	K.ELMFIQVGEK.S + Oxidation (M)
3251	29 - 38	605.3127	1208.6108	1208.6111	-0.24	0	25	0.0031	1	U	K.ELMFIQVGEK.S + Oxidation (M)
3252	29 - 38	605.3128	1208.6111	1208.6111	-0.034	0	14	0.04	1	U	K.ELMFIQVGEK.S + Oxidation (M)
3253	29 - 38	605.3128	1208.6111	1208.6111	-0.034	0	20	0.0091	1	U	K.ELMFIQVGEK.S + Oxidation (M)
3254	29 - 38	605.3128	1208.6111	1208.6111	-0.034	0	25	0.0033	1	U	K.ELMFIQVGEK.S + Oxidation (M)
3255	29 - 38	605.3128	1208.6111	1208.6111	-0.034	0	12	0.057	1	U	K.ELMFIQVGEK.S + Oxidation (M)

Query	Start - End	Observed	Mr(expt)	Mr(calc)	ppm	M	Score	Expect	Rank	U	Peptide
3256	29 - 38	605.3129	1208.6113	1208.6111	0.17	0	18	0.016	1	U	K.ELMFIQVGEK.S + Oxidation (M)
3257	29 - 38	605.3130	1208.6115	1208.6111	0.37	0	20	0.01	1	U	K.ELMFIQVGEK.S + Oxidation (M)
3258	29 - 38	605.3131	1208.6117	1208.6111	0.47	0	33	0.00052	1	U	K.ELMFIQVGEK.S + Oxidation (M)
3259	29 - 38	605.3132	1208.6119	1208.6111	0.67	0	26	0.0025	1	U	K.ELMFIQVGEK.S + Oxidation (M)
3260	29 - 38	605.3132	1208.6119	1208.6111	0.67	0	34	0.00039	1	U	K.ELMFIQVGEK.S + Oxidation (M)
3261	29 - 38	605.3133	1208.6120	1208.6111	0.77	0	17	0.021	1	U	K.ELMFIQVGEK.S + Oxidation (M)
3262	29 - 38	605.3133	1208.6120	1208.6111	0.77	0	57	1.8e-006	1	U	K.ELMFIQVGEK.S + Oxidation (M)
3263	29 - 38	605.3133	1208.6120	1208.6111	0.77	0	13	0.048	1	U	K.ELMFIQVGEK.S + Oxidation (M)
3264	29 - 38	605.3134	1208.6122	1208.6111	0.87	0	26	0.0026	1	U	K.ELMFIQVGEK.S + Oxidation (M)
3265	29 - 38	605.3134	1208.6123	1208.6111	0.98	0	20	0.011	1	U	K.ELMFIQVGEK.S + Oxidation (M)
3266	29 - 38	605.3134	1208.6123	1208.6111	0.98	0	16	0.024	1	U	K.ELMFIQVGEK.S + Oxidation (M)
3267	29 - 38	605.3134	1208.6123	1208.6111	0.98	0	4	0.36	1	U	K.ELMFIQVGEK.S + Oxidation (M)
3268	29 - 38	605.3135	1208.6124	1208.6111	1.08	0	11	0.084	1	U	K.ELMFIQVGEK.S + Oxidation (M)
3269	29 - 38	605.3135	1208.6125	1208.6111	1.18	0	25	0.0029	1	U	K.ELMFIQVGEK.S + Oxidation (M)
3270	29 - 38	605.3137	1208.6129	1208.6111	1.48	0	29	0.0012	1	U	K.ELMFIQVGEK.S + Oxidation (M)
3271	29 - 38	605.3146	1208.6147	1208.6111	3.00	0	21	0.0085	1	U	K.ELMFIQVGEK.S + Oxidation (M)
434	39 - 44	353.6811	705.3477	705.3479	-0.34	0	37	0.00018	1	U	K.SNLMNK.A
435	39 - 44	353.6812	705.3478	705.3479	-0.25	0	27	0.0021	1	U	K.SNLMNK.A
467	39 - 44	361.6786	721.3426	721.3429	-0.40	0	12	0.057	1	U	K.SNLMNK.A + Oxidation (M)
468	39 - 44	361.6786	721.3426	721.3429	-0.31	0	29	0.0014	1	U	K.SNLMNK.A + Oxidation (M)
469	39 - 44	361.6787	721.3427	721.3429	-0.14	0	25	0.0034	1	U	K.SNLMNK.A + Oxidation (M)
470	39 - 44	361.6787	721.3427	721.3429	-0.14	0	26	0.0028	1	U	K.SNLMNK.A + Oxidation (M)
471	39 - 44	361.6787	721.3428	721.3429	-0.058	0	27	0.0018	1	U	K.SNLMNK.A + Oxidation (M)
4279	47 - 58	693.8682	1385.7219	1385.7263	-3.16	0	47	2e-005	1	U	R.INTDAQQIQSIR.W
4280	47 - 58	693.8687	1385.7228	1385.7263	-2.54	0	36	0.00027	1	U	R.INTDAQQIQSIR.W
4281	47 - 58	693.8699	1385.7253	1385.7263	-0.69	0	60	8.9e-007	1	U	R.INTDAQQIQSIR.W
4282	47 - 58	693.8701	1385.7256	1385.7263	-0.51	0	33	0.00054	1	U	R.INTDAQQIQSIR.W
4283	47 - 58	693.8701	1385.7257	1385.7263	-0.43	0	65	3.3e-007	1	U	R.INTDAQQIQSIR.W
4284	47 - 58	462.9160	1385.7261	1385.7263	-0.11	0	37	0.00021	1	U	R.INTDAQQIQSIR.W
4285	47 - 58	693.8704	1385.7262	1385.7263	-0.074	0	26	0.0023	1	U	R.INTDAQQIQSIR.W
4286	47 - 58	693.8704	1385.7262	1385.7263	-0.074	0	39	0.00011	1	U	R.INTDAQQIQSIR.W
4287	47 - 58	693.8704	1385.7262	1385.7263	-0.074	0	79	1.1e-008	1	U	R.INTDAQQIQSIR.W
4288	47 - 58	693.8704	1385.7262	1385.7263	-0.074	0	85	3.3e-009	1	U	R.INTDAQQIQSIR.W
4289	47 - 58	693.8704	1385.7263	1385.7263	0.014	0	34	0.00036	1	U	R.INTDAQQIQSIR.W
4290	47 - 58	693.8705	1385.7264	1385.7263	0.10	0	51	8.2e-006	1	U	R.INTDAQQIQSIR.W
4291	47 - 58	693.8705	1385.7264	1385.7263	0.10	0	67	2e-007	1	U	R.INTDAQQIQSIR.W
4292	47 - 58	693.8705	1385.7264	1385.7263	0.10	0	65	3.1e-007	1	U	R.INTDAQQIQSIR.W
4293	47 - 58	693.8707	1385.7268	1385.7263	0.37	0	36	0.00023	1	U	R.INTDAQQIQSIR.W
4294	47 - 58	693.8707	1385.7269	1385.7263	0.46	0	37	0.00021	1	U	R.INTDAQQIQSIR.W
4295	47 - 58	693.8708	1385.7270	1385.7263	0.54	0	42	6.9e-005	1	U	R.INTDAQQIQSIR.W
4296	47 - 58	693.8708	1385.7270	1385.7263	0.54	0	57	2e-006	1	U	R.INTDAQQIQSIR.W
4297	47 - 58	693.8709	1385.7271	1385.7263	0.63	0	18	0.015	1	U	R.INTDAQQIQSIR.W
4298	47 - 58	693.8709	1385.7273	1385.7263	0.72	0	18	0.017	1	U	R.INTDAQQIQSIR.W
4299	47 - 58	462.9164	1385.7275	1385.7263	0.89	0	42	5.9e-005	1	U	R.INTDAQQIQSIR.W
4300	47 - 58	693.8710	1385.7275	1385.7263	0.90	0	22	0.0057	1	U	R.INTDAQQIQSIR.W
4301	47 - 58	693.8710	1385.7275	1385.7263	0.90	0	70	1.1e-007	1	U	R.INTDAQQIQSIR.W
4302	47 - 58	693.8711	1385.7276	1385.7263	0.98	0	46	2.3e-005	1	U	R.INTDAQQIQSIR.W
4303	47 - 58	693.8712	1385.7278	1385.7263	1.07	0	13	0.047	1	U	R.INTDAQQIQSIR.W
4304	47 - 58	693.8712	1385.7279	1385.7263	1.16	0	47	1.8e-005	1	U	R.INTDAQQIQSIR.W
4305	47 - 58	693.8713	1385.7280	1385.7263	1.25	0	32	0.0006	1	U	R.INTDAQQIQSIR.W
4306	47 - 58	693.8713	1385.7280	1385.7263	1.25	0	45	2.9e-005	1	U	R.INTDAQQIQSIR.W
4307	47 - 58	693.8713	1385.7280	1385.7263	1.25	0	5	0.31	1	U	R.INTDAQQIQSIR.W
4308	47 - 58	693.8713	1385.7280	1385.7263	1.25	0	44	4.1e-005	1	U	R.INTDAQQIQSIR.W
4309	47 - 58	693.8713	1385.7281	1385.7263	1.34	0	15	0.032	1	U	R.INTDAQQIQSIR.W
4310	47 - 58	693.8713	1385.7281	1385.7263	1.34	0	37	0.00022	1	U	R.INTDAQQIQSIR.W
4311	47 - 58	693.8714	1385.7282	1385.7263	1.42	0	30	0.0011	1	U	R.INTDAQQIQSIR.W
4312	47 - 58	693.8714	1385.7282	1385.7263	1.42	0	31	0.00078	1	U	R.INTDAQQIQSIR.W
4313	47 - 58	693.8715	1385.7284	1385.7263	1.51	0	26	0.0024	1	U	R.INTDAQQIQSIR.W
4314	47 - 58	693.8716	1385.7287	1385.7263	1.78	0	3	0.53	1	U	R.INTDAQQIQSIR.W
4315	47 - 58	693.8718	1385.7291	1385.7263	2.04	0	29	0.0013	1	U	R.INTDAQQIQSIR.W
4316	47 - 58	693.8724	1385.7303	1385.7263	2.92	0	18	0.017	1	U	R.INTDAQQIQSIR.W
8714	47 - 64	711.3703	2131.0891	2131.0844	2.19	1	18	0.014	1	U	R.INTDAQQIQSIRWMGNLK.S + Oxidation (M)
555	59 - 64	374.6934	747.3723	747.3738	-1.97	0	8	0.16	1	U	R.WMGNLK.S
603	59 - 64	382.6911	763.3677	763.3687	-1.27	0	30	0.001	1	U	R.WMGNLK.S + Oxidation (M)
604	59 - 64	382.6913	763.3680	763.3687	-0.87	0	12	0.057	1	U	R.WMGNLK.S + Oxidation (M)
605	59 - 64	382.6915	763.3684	763.3687	-0.31	0	16	0.027	1	U	R.WMGNLK.S + Oxidation (M)
606	59 - 64	382.6916	763.3687	763.3687	0.085	0	29	0.0011	1	U	R.WMGNLK.S + Oxidation (M)
607	59 - 64	382.6916	763.3687	763.3687	0.085	0	13	0.049	1	U	R.WMGNLK.S + Oxidation (M)
608	59 - 64	382.6916	763.3687	763.3687	0.085	0	27	0.002	1	U	R.WMGNLK.S + Oxidation (M)
609	59 - 64	382.6917	763.3688	763.3687	0.17	0	17	0.022	1	U	R.WMGNLK.S + Oxidation (M)
610	59 - 64	382.6917	763.3689	763.3687	0.24	0	15	0.029	1	U	R.WMGNLK.S + Oxidation (M)
611	59 - 64	382.6918	763.3691	763.3687	0.49	0	21	0.0087	1	U	R.WMGNLK.S + Oxidation (M)
612	59 - 64	382.6919	763.3693	763.3687	0.81	0	27	0.0021	1	U	R.WMGNLK.S + Oxidation (M)
1507	65 - 72	469.2219	936.4293	936.4301	-0.86	0	32	0.00063	1	U	K.SPQTGEYR.L
1508	65 - 72	469.2219	936.4293	936.4301	-0.86	0	23	0.0045	1	U	K.SPQTGEYR.L
1509	65 - 72	469.2221	936.4297	936.4301	-0.40	0	12	0.062	1	U	K.SPQTGEYR.L
1510	65 - 72	469.2223	936.4300	936.4301	-0.076	0	11	0.078	1	U	K.SPQTGEYR.L
1511	65 - 72	469.2224	936.4302	936.4301	0.054	0	4	0.42	1	U	K.SPQTGEYR.L
1512	65 - 72	469.2224	936.4302	936.4301	0.12	0	1	0.89	1	U	K.SPQTGEYR.L
1513	65 - 72	469.2224	936.4303	936.4301	0.25	0	6	0.24	1	U	K.SPQTGEYR.L
1514	65 - 72	469.2224	936.4303	936.4301	0.25	0	32	0.00059	1	U	K.SPQTGEYR.L
1515	65 - 72	469.2224	936.4303	936.4301	0.25	0	35	0.00034	1	U	K.SPQTGEYR.L
1516	65 - 72	469.2225	936.4305	936.4301	0.45	0	47	2e-005	1	U	K.SPQTGEYR.L
1517	65 - 72	469.2226	936.4306	936.4301	0.51	0	4	0.37	1	U	K.SPQTGEYR.L
1519	65 - 72	469.2227	936.4308	936.4301	0.71	0	4	0.38	1	U	K.SPQTGEYR.L
1520	65 - 72	469.2227	936.4309	936.4301	0.90	0	5	0.28	1	U	K.SPQTGEYR.L
1521	65 - 72	469.2228	936.4311	936.4301	1.10	0	44	4.3e-005	1	U	K.SPQTGEYR.L
1523	65 - 72	469.2234	936.4323	936.4301	2.34	0	17	0.022	1	U	K.SPQTGEYR.L

Query	Start - End	Observed	Mr(expt)	Mr(calc)	ppm	M	Score	Expect	Rank	U	Peptide
11827	73 - 98	934.4849	2800.4329	2800.4454	-4.45	0	49	1.4e-005	1	U	R.LSTSSDENVILQINGETVINQASIQK.N
11828	73 - 98	934.4849	2800.4329	2800.4454	-4.45	0	53	4.9e-006	1	U	R.LSTSSDENVILQINGETVINQASIQK.N
11829	73 - 98	934.4851	2800.4335	2800.4454	-4.25	0	46	2.2e-005	1	U	R.LSTSSDENVILQINGETVINQASIQK.N
11830	73 - 98	934.4854	2800.4342	2800.4454	-3.99	0	57	2.2e-006	1	U	R.LSTSSDENVILQINGETVINQASIQK.N
11831	73 - 98	934.4857	2800.4351	2800.4454	-3.66	0	63	5.1e-007	1	U	R.LSTSSDENVILQINGETVINQASIQK.N
11832	73 - 98	934.4858	2800.4355	2800.4454	-3.53	0	50	9.6e-006	1	U	R.LSTSSDENVILQINGETVINQASIQK.N
11833	73 - 98	934.4858	2800.4355	2800.4454	-3.53	0	53	5e-006	1	U	R.LSTSSDENVILQINGETVINQASIQK.N
11834	73 - 98	934.4861	2800.4364	2800.4454	-3.21	0	56	2.6e-006	1	U	R.LSTSSDENVILQINGETVINQASIQK.N
11835	73 - 98	934.4861	2800.4366	2800.4454	-3.14	0	46	2.4e-005	1	U	R.LSTSSDENVILQINGETVINQASIQK.N
11836	73 - 98	934.4861	2800.4366	2800.4454	-3.14	0	50	9.5e-006	1	U	R.LSTSSDENVILQINGETVINQASIQK.N
11837	73 - 98	934.4863	2800.4370	2800.4454	-3.01	0	56	2.6e-006	1	U	R.LSTSSDENVILQINGETVINQASIQK.N
11838	73 - 98	934.4863	2800.4370	2800.4454	-3.01	0	59	1.2e-006	1	U	R.LSTSSDENVILQINGETVINQASIQK.N
11839	73 - 98	934.4863	2800.4372	2800.4454	-2.95	0	48	1.5e-005	1	U	R.LSTSSDENVILQINGETVINQASIQK.N
11840	73 - 98	1401.2260	2800.4374	2800.4454	-2.88	0	29	0.0013	1	U	R.LSTSSDENVILQINGETVINQASIQK.N
11841	73 - 98	934.4865	2800.4377	2800.4454	-2.75	0	59	1.3e-006	1	U	R.LSTSSDENVILQINGETVINQASIQK.N
11842	73 - 98	934.4865	2800.4377	2800.4454	-2.75	0	59	1.4e-006	1	U	R.LSTSSDENVILQINGETVINQASIQK.N
11843	73 - 98	1401.2264	2800.4383	2800.4454	-2.53	0	57	1.9e-006	1	U	R.LSTSSDENVILQINGETVINQASIQK.N
11844	73 - 98	934.4869	2800.4388	2800.4454	-2.36	0	59	1.3e-006	1	U	R.LSTSSDENVILQINGETVINQASIQK.N
11845	73 - 98	934.4871	2800.4395	2800.4454	-2.09	0	52	6.1e-006	1	U	R.LSTSSDENVILQINGETVINQASIQK.N
11846	73 - 98	1401.2273	2800.4400	2800.4454	-1.92	0	10	0.092	1	U	R.LSTSSDENVILQINGETVINQASIQK.N
11847	73 - 98	1401.2273	2800.4400	2800.4454	-1.92	0	33	0.00055	1	U	R.LSTSSDENVILQINGETVINQASIQK.N
11848	73 - 98	934.4876	2800.4410	2800.4454	-1.57	0	59	1.4e-006	1	U	R.LSTSSDENVILQINGETVINQASIQK.N
11849	73 - 98	934.4876	2800.4410	2800.4454	-1.57	0	50	1.1e-005	1	U	R.LSTSSDENVILQINGETVINQASIQK.N
11850	73 - 98	1401.2279	2800.4413	2800.4454	-1.48	0	23	0.0056	1	U	R.LSTSSDENVILQINGETVINQASIQK.N
11851	73 - 98	934.4877	2800.4414	2800.4454	-1.44	0	54	4e-006	1	U	R.LSTSSDENVILQINGETVINQASIQK.N
11852	73 - 98	934.4878	2800.4416	2800.4454	-1.38	0	52	6.3e-006	1	U	R.LSTSSDENVILQINGETVINQASIQK.N
11853	73 - 98	934.4879	2800.4417	2800.4454	-1.31	0	45	3.4e-005	1	U	R.LSTSSDENVILQINGETVINQASIQK.N
11854	73 - 98	934.4879	2800.4419	2800.4454	-1.25	0	44	4e-005	1	U	R.LSTSSDENVILQINGETVINQASIQK.N
11855	73 - 98	934.4881	2800.4425	2800.4454	-1.05	0	50	9.1e-006	1	U	R.LSTSSDENVILQINGETVINQASIQK.N
11856	73 - 98	934.4882	2800.4426	2800.4454	-0.98	0	52	6e-006	1	U	R.LSTSSDENVILQINGETVINQASIQK.N
11857	73 - 98	934.4882	2800.4428	2800.4454	-0.92	0	43	5.2e-005	1	U	R.LSTSSDENVILQINGETVINQASIQK.N
11858	73 - 98	1401.2289	2800.4432	2800.4454	-0.78	0	16	0.024	1	U	R.LSTSSDENVILQINGETVINQASIQK.N
11860	73 - 98	934.4885	2800.4437	2800.4454	-0.59	0	58	1.5e-006	1	U	R.LSTSSDENVILQINGETVINQASIQK.N
11861	73 - 98	934.4886	2800.4439	2800.4454	-0.53	0	52	5.9e-006	1	U	R.LSTSSDENVILQINGETVINQASIQK.N
11862	73 - 98	1401.2295	2800.4444	2800.4454	-0.35	0	7	0.2	1	U	R.LSTSSDENVILQINGETVINQASIQK.N
11863	73 - 98	934.4888	2800.4445	2800.4454	-0.33	0	52	6e-006	1	U	R.LSTSSDENVILQINGETVINQASIQK.N
11864	73 - 98	934.4888	2800.4447	2800.4454	-0.26	0	52	6e-006	1	U	R.LSTSSDENVILQINGETVINQASIQK.N
11865	73 - 98	1401.2296	2800.4447	2800.4454	-0.26	0	27	0.0021	1	U	R.LSTSSDENVILQINGETVINQASIQK.N
11866	73 - 98	934.4890	2800.4450	2800.4454	-0.13	0	48	1.7e-005	1	U	R.LSTSSDENVILQINGETVINQASIQK.N
11867	73 - 98	934.4891	2800.4456	2800.4454	0.063	0	51	7.9e-006	1	U	R.LSTSSDENVILQINGETVINQASIQK.N
11868	73 - 98	1401.2302	2800.4459	2800.4454	0.18	0	25	0.0035	1	U	R.LSTSSDENVILQINGETVINQASIQK.N
11869	73 - 98	934.4893	2800.4459	2800.4454	0.19	0	46	2.7e-005	1	U	R.LSTSSDENVILQINGETVINQASIQK.N
11871	73 - 98	1401.2306	2800.4466	2800.4454	0.44	0	44	3.7e-005	1	U	R.LSTSSDENVILQINGETVINQASIQK.N
11872	73 - 98	1401.2306	2800.4466	2800.4454	0.44	0	85	3.2e-009	1	U	R.LSTSSDENVILQINGETVINQASIQK.N
11873	73 - 98	934.4896	2800.4469	2800.4454	0.52	0	51	8e-006	1	U	R.LSTSSDENVILQINGETVINQASIQK.N
11874	73 - 98	701.1190	2800.4470	2800.4454	0.56	0	57	2.2e-006	1	U	R.LSTSSDENVILQINGETVINQASIQK.N
11875	73 - 98	934.4896	2800.4470	2800.4454	0.59	0	63	4.9e-007	1	U	R.LSTSSDENVILQINGETVINQASIQK.N
11876	73 - 98	934.4897	2800.4474	2800.4454	0.72	0	55	3e-006	1	U	R.LSTSSDENVILQINGETVINQASIQK.N
11877	73 - 98	1401.2311	2800.4476	2800.4454	0.79	0	62	6.2e-007	1	U	R.LSTSSDENVILQINGETVINQASIQK.N
11878	73 - 98	934.4899	2800.4478	2800.4454	0.85	0	34	0.00037	1	U	R.LSTSSDENVILQINGETVINQASIQK.N
11880	73 - 98	1401.2317	2800.4488	2800.4454	1.22	0	1	0.8	1	U	R.LSTSSDENVILQINGETVINQASIQK.N
11881	73 - 98	1401.2319	2800.4493	2800.4454	1.40	0	16	0.023	1	U	R.LSTSSDENVILQINGETVINQASIQK.N
11883	73 - 98	1401.2321	2800.4496	2800.4454	1.48	0	31	0.00087	1	U	R.LSTSSDENVILQINGETVINQASIQK.N
11884	73 - 98	1401.2323	2800.4500	2800.4454	1.66	0	16	0.026	1	U	R.LSTSSDENVILQINGETVINQASIQK.N
11886	73 - 98	1401.2329	2800.4513	2800.4454	2.09	0	8	0.18	1	U	R.LSTSSDENVILQINGETVINQASIQK.N
11887	73 - 98	1401.2329	2800.4513	2800.4454	2.09	0	12	0.063	1	U	R.LSTSSDENVILQINGETVINQASIQK.N
11891	73 - 98	1401.2335	2800.4525	2800.4454	2.53	0	4	0.36	1	U	R.LSTSSDENVILQINGETVINQASIQK.N
11892	73 - 98	934.4916	2800.4531	2800.4454	2.74	0	47	2e-005	1	U	R.LSTSSDENVILQINGETVINQASIQK.N
11893	73 - 98	1401.2351	2800.4557	2800.4454	3.66	0	10	0.1	1	U	R.LSTSSDENVILQINGETVINQASIQK.N
11894	73 - 98	1401.2354	2800.4562	2800.4454	3.84	0	10	0.11	1	U	R.LSTSSDENVILQINGETVINQASIQK.N
11895	73 - 98	934.4927	2800.4562	2800.4454	3.86	0	47	1.9e-005	1	U	R.LSTSSDENVILQINGETVINQASIQK.N
5034	99 - 111	521.2885	1560.8435	1560.8511	-4.85	1	33	0.00054	1	U	K.NLKLEANQVYEIK.I
5035	99 - 111	521.2889	1560.8448	1560.8511	-4.03	1	35	0.00033	1	U	K.NLKLEANQVYEIK.I
5036	99 - 111	521.2894	1560.8465	1560.8511	-2.98	1	33	0.00047	1	U	K.NLKLEANQVYEIK.I
5037	99 - 111	781.4311	1560.8476	1560.8511	-2.23	1	38	0.00018	1	U	K.NLKLEANQVYEIK.I
5038	99 - 111	521.2910	1560.8510	1560.8511	-0.044	1	30	0.001	1	U	K.NLKLEANQVYEIK.I
5039	99 - 111	521.2910	1560.8512	1560.8511	0.074	1	21	0.0082	1	U	K.NLKLEANQVYEIK.I
5040	99 - 111	521.2910	1560.8512	1560.8511	0.074	1	36	0.00025	1	U	K.NLKLEANQVYEIK.I
5041	99 - 111	521.2910	1560.8512	1560.8511	0.074	1	32	0.00064	1	U	K.NLKLEANQVYEIK.I
5042	99 - 111	781.4329	1560.8513	1560.8511	0.12	1	60	1.1e-006	1	U	K.NLKLEANQVYEIK.I
5043	99 - 111	781.4329	1560.8513	1560.8511	0.12	1	59	1.4e-006	1	U	K.NLKLEANQVYEIK.I
5044	99 - 111	521.2911	1560.8514	1560.8511	0.19	1	11	0.071	1	U	K.NLKLEANQVYEIK.I
5045	99 - 111	521.2911	1560.8516	1560.8511	0.31	1	29	0.0014	1	U	K.NLKLEANQVYEIK.I
5046	99 - 111	521.2911	1560.8516	1560.8511	0.31	1	40	9.5e-005	1	U	K.NLKLEANQVYEIK.I
5047	99 - 111	781.4331	1560.8517	1560.8511	0.35	1	65	2.9e-007	1	U	K.NLKLEANQVYEIK.I
5048	99 - 111	521.2912	1560.8518	1560.8511	0.43	1	27	0.0021	1	U	K.NLKLEANQVYEIK.I
5049	99 - 111	521.2912	1560.8518	1560.8511	0.43	1	28	0.0017	1	U	K.NLKLEANQVYEIK.I
5050	99 - 111	521.2912	1560.8518	1560.8511	0.43	1	27	0.0019	1	U	K.NLKLEANQVYEIK.I
5051	99 - 111	521.2912	1560.8518	1560.8511	0.43	1	34	0.00044	1	U	K.NLKLEANQVYEIK.I
5052	99 - 111	521.2913	1560.8520	1560.8511	0.54	1	37	0.00018	1	U	K.NLKLEANQVYEIK.I
5053	99 - 111	781.4335	1560.8525	1560.8511	0.90	1	56	2.4e-006	1	U	K.NLKLEANQVYEIK.I
5054	99 - 111	521.2915	1560.8527	1560.8511	1.01	1	24	0.0042	1	U	K.NLKLEANQVYEIK.I
5055	99 - 111	521.2915	1560.8527	1560.8511	1.01	1	30	0.00091	1	U	K.NLKLEANQVYEIK.I
5056	99 - 111	521.2915	1560.8527	1560.8511	1.01	1	37	0.0002	1	U	K.NLKLEANQVYEIK.I
5057	99 - 111	521.2916	1560.8529	1560.8511	1.13	1	31	0.00087	1	U	K.NLKLEANQVYEIK.I
5058	99 - 111	781.4338	1560.8530	1560.8511	1.22	1	5	0.29	1	U	K.NLKLEANQVYEIK.I
5059	99 - 111	521.2916	1560.8530	1560.8511	1.25	1	25	0.0029	1	U	K.NLKLEANQVYEIK.I
5060	99 - 111	521.2916	1560.8530	1560.8511	1.25	1	37	0.00022	1	U	K.NLKLEANQVYEIK.I

Query	Start - End	Observed	Mr(expt)	Mr(calc)	ppm	M	Score	Expect	Rank	U	Peptide
5061	99 - 111	521.2917	1560.8534	1560.8511	1.48	1	32	0.00063	1	U	K.NLKLEANQVYEIK.I
5062	99 - 111	781.4340	1560.8535	1560.8511	1.53	1	54	3.5e-006	1	U	K.NLKLEANQVYEIK.I
5063	99 - 111	521.2918	1560.8536	1560.8511	1.60	1	28	0.0017	1	U	K.NLKLEANQVYEIK.I
5064	99 - 111	781.4344	1560.8543	1560.8511	2.08	1	35	0.0003	1	U	K.NLKLEANQVYEIK.I
5065	99 - 111	781.4349	1560.8552	1560.8511	2.62	1	23	0.0052	1	U	K.NLKLEANQVYEIK.I
5066	99 - 111	781.4349	1560.8552	1560.8511	2.62	1	62	7e-007	1	U	K.NLKLEANQVYEIK.I
5067	99 - 111	521.2926	1560.8560	1560.8511	3.12	1	37	0.00018	1	U	K.NLKLEANQVYEIK.I
5068	99 - 111	781.4357	1560.8568	1560.8511	3.64	1	51	8.7e-006	1	U	K.NLKLEANQVYEIK.I
8647	99 - 115	708.3891	2122.1455	2122.1422	1.55	2	7	0.19	1	U	K.NLKLEANQVYEIKIEYR.N
3190	102 - 111	603.8193	1205.6241	1205.6292	-4.19	0	41	8e-005	1	U	K.LEANQVYEIK.I
3192	102 - 111	603.8215	1205.6285	1205.6292	-0.54	0	20	0.0096	1	U	K.LEANQVYEIK.I
3193	102 - 111	603.8215	1205.6285	1205.6292	-0.54	0	30	0.001	1	U	K.LEANQVYEIK.I
3194	102 - 111	603.8215	1205.6285	1205.6292	-0.54	0	28	0.0017	1	U	K.LEANQVYEIK.I
3195	102 - 111	603.8215	1205.6285	1205.6292	-0.54	0	57	1.9e-006	1	U	K.LEANQVYEIK.I
3196	102 - 111	603.8216	1205.6286	1205.6292	-0.44	0	22	0.0067	1	U	K.LEANQVYEIK.I
3197	102 - 111	603.8217	1205.6289	1205.6292	-0.24	0	23	0.0052	1	U	K.LEANQVYEIK.I
3198	102 - 111	603.8217	1205.6289	1205.6292	-0.24	0	19	0.012	1	U	K.LEANQVYEIK.I
3199	102 - 111	603.8217	1205.6289	1205.6292	-0.24	0	22	0.0064	1	U	K.LEANQVYEIK.I
3200	102 - 111	603.8218	1205.6291	1205.6292	-0.035	0	35	0.00031	1	U	K.LEANQVYEIK.I
3201	102 - 111	603.8218	1205.6291	1205.6292	-0.035	0	38	0.00018	1	U	K.LEANQVYEIK.I
3202	102 - 111	603.8219	1205.6292	1205.6292	0.066	0	41	7.3e-005	1	U	K.LEANQVYEIK.I
3203	102 - 111	603.8219	1205.6292	1205.6292	0.066	0	53	4.6e-006	1	U	K.LEANQVYEIK.I
3204	102 - 111	603.8219	1205.6292	1205.6292	0.066	0	66	2.3e-007	1	U	K.LEANQVYEIK.I
3205	102 - 111	603.8220	1205.6294	1205.6292	0.17	0	15	0.028	1	U	K.LEANQVYEIK.I
3206	102 - 111	603.8220	1205.6295	1205.6292	0.27	0	32	0.00068	1	U	K.LEANQVYEIK.I
3207	102 - 111	603.8220	1205.6295	1205.6292	0.27	0	23	0.0054	1	U	K.LEANQVYEIK.I
3208	102 - 111	603.8220	1205.6295	1205.6292	0.27	0	58	1.8e-006	1	U	K.LEANQVYEIK.I
3209	102 - 111	603.8220	1205.6295	1205.6292	0.27	0	51	7.5e-006	1	U	K.LEANQVYEIK.I
3210	102 - 111	603.8221	1205.6296	1205.6292	0.37	0	20	0.0096	1	U	K.LEANQVYEIK.I
3211	102 - 111	603.8221	1205.6297	1205.6292	0.47	0	4	0.39	1	U	K.LEANQVYEIK.I
3212	102 - 111	603.8221	1205.6297	1205.6292	0.47	0	9	0.12	1	U	K.LEANQVYEIK.I
3213	102 - 111	603.8222	1205.6299	1205.6292	0.57	0	26	0.0026	1	U	K.LEANQVYEIK.I
3214	102 - 111	603.8222	1205.6299	1205.6292	0.57	0	36	0.00025	1	U	K.LEANQVYEIK.I
3215	102 - 111	603.8222	1205.6299	1205.6292	0.57	0	29	0.0013	1	U	K.LEANQVYEIK.I
3216	102 - 111	603.8223	1205.6300	1205.6292	0.68	0	28	0.0017	1	U	K.LEANQVYEIK.I
3217	102 - 111	603.8223	1205.6300	1205.6292	0.68	0	46	2.4e-005	1	U	K.LEANQVYEIK.I
3218	102 - 111	603.8223	1205.6300	1205.6292	0.68	0	5	0.3	1	U	K.LEANQVYEIK.I
3219	102 - 111	603.8223	1205.6300	1205.6292	0.68	0	31	0.00088	1	U	K.LEANQVYEIK.I
3220	102 - 111	603.8223	1205.6301	1205.6292	0.78	0	15	0.035	1	U	K.LEANQVYEIK.I
3221	102 - 111	603.8223	1205.6301	1205.6292	0.78	0	25	0.0031	1	U	K.LEANQVYEIK.I
3222	102 - 111	603.8224	1205.6303	1205.6292	0.98	0	36	0.00028	1	U	K.LEANQVYEIK.I
3223	102 - 111	603.8226	1205.6306	1205.6292	1.18	0	8	0.17	1	U	K.LEANQVYEIK.I
3224	102 - 111	603.8226	1205.6307	1205.6292	1.28	0	31	0.00079	1	U	K.LEANQVYEIK.I
3225	102 - 111	603.8228	1205.6310	1205.6292	1.48	0	3	0.45	1	U	K.LEANQVYEIK.I
3226	102 - 111	402.8843	1205.6311	1205.6292	1.60	0	34	0.00041	1	U	K.LEANQVYEIK.I
3227	102 - 111	603.8229	1205.6312	1205.6292	1.69	0	19	0.013	1	U	K.LEANQVYEIK.I
3228	102 - 111	603.8231	1205.6316	1205.6292	1.99	0	20	0.0099	1	U	K.LEANQVYEIK.I
160	112 - 115	290.6581	579.3016	579.3016	-0.045	0	18	0.016	1	U	K.IEYR.N
161	112 - 115	290.6582	579.3019	579.3016	0.48	0	19	0.013	1	U	K.IEYR.N
162	112 - 115	290.6582	579.3019	579.3016	0.48	0	12	0.069	1	U	K.IEYR.N
163	112 - 115	290.6583	579.3021	579.3016	0.80	0	19	0.012	1	U	K.IEYR.N
164	112 - 115	290.6584	579.3022	579.3016	0.90	0	14	0.041	1	U	K.IEYR.N
9456	116 - 135	774.7084	2321.1035	2321.1110	-3.24	0	6	0.24	1	U	R.NTSNTLPDLQLFWSMNNAQK.E
9458	116 - 135	774.7118	2321.1135	2321.1110	1.09	0	46	2.8e-005	1	U	R.NTSNTLPDLQLFWSMNNAQK.E
9459	116 - 135	1161.5657	2321.1168	2321.1110	2.49	0	46	2.5e-005	1	U	R.NTSNTLPDLQLFWSMNNAQK.E
9517	116 - 135	780.0416	2337.1029	2337.1059	-1.31	0	37	0.00018	1	U	R.NTSNTLPDLQLFWSMNNAQK.E + Oxidation (M)
9518	116 - 135	780.0421	2337.1045	2337.1059	-0.60	0	32	0.00056	1	U	R.NTSNTLPDLQLFWSMNNAQK.E + Oxidation (M)
9519	116 - 135	1169.5603	2337.1061	2337.1059	0.054	0	41	8.6e-005	1	U	R.NTSNTLPDLQLFWSMNNAQK.E + Oxidation (M)
9521	116 - 135	1169.5607	2337.1068	2337.1059	0.37	0	20	0.01	1	U	R.NTSNTLPDLQLFWSMNNAQK.E + Oxidation (M)
9522	116 - 135	1169.5607	2337.1068	2337.1059	0.37	0	22	0.0058	1	U	R.NTSNTLPDLQLFWSMNNAQK.E + Oxidation (M)
9523	116 - 135	780.0429	2337.1069	2337.1059	0.41	0	41	8.4e-005	1	U	R.NTSNTLPDLQLFWSMNNAQK.E + Oxidation (M)
9524	116 - 135	1169.5608	2337.1070	2337.1059	0.47	0	23	0.0056	1	U	R.NTSNTLPDLQLFWSMNNAQK.E + Oxidation (M)
9525	116 - 135	780.0430	2337.1073	2337.1059	0.57	0	29	0.0012	1	U	R.NTSNTLPDLQLFWSMNNAQK.E + Oxidation (M)
9526	116 - 135	780.0435	2337.1085	2337.1059	1.12	0	12	0.068	1	U	R.NTSNTLPDLQLFWSMNNAQK.E + Oxidation (M)
9527	116 - 135	1169.5635	2337.1124	2337.1059	2.77	0	44	4e-005	1	U	R.NTSNTLPDLQLFWSMNNAQK.E + Oxidation (M)
13181	116 - 141	766.3784	3061.4846	3061.4815	1.01	1	17	0.02	1	U	R.NTSNTLPDLQLFWSMNNAQKEQIPEK.Y + Oxidation (M)
13182	116 - 141	1021.5031	3061.4875	3061.4815	1.98	1	32	0.00065	1	U	R.NTSNTLPDLQLFWSMNNAQKEQIPEK.Y + Oxidation (M)
534	136 - 141	372.2001	742.3857	742.3861	-0.59	0	4	0.39	1	U	K.EQIPEK.Y
535	136 - 141	372.2004	742.3863	742.3861	0.23	0	13	0.05	1	U	K.EQIPEK.Y
536	136 - 141	372.2007	742.3869	742.3861	1.05	0	18	0.016	1	U	K.EQIPEK.Y
6992	136 - 151	641.3355	1920.9847	1920.9832	0.76	1	15	0.03	1	U	K.EQIPEKYILSPNFSEK.A
6993	136 - 151	961.5025	1920.9905	1920.9832	3.75	1	68	1.5e-007	1	U	K.EQIPEKYILSPNFSEK.A
3057	142 - 151	599.3086	1196.6026	1196.6077	-4.24	0	50	1.1e-005	1	U	K.YILSPNFSEK.A
3058	142 - 151	599.3104	1196.6062	1196.6077	-1.28	0	13	0.055	1	U	K.YILSPNFSEK.A
3059	142 - 151	599.3107	1196.6068	1196.6077	-0.77	0	25	0.0032	1	U	K.YILSPNFSEK.A
3060	142 - 151	599.3107	1196.6069	1196.6077	-0.67	0	27	0.0018	1	U	K.YILSPNFSEK.A
3061	142 - 151	599.3107	1196.6069	1196.6077	-0.67	0	25	0.0032	1	U	K.YILSPNFSEK.A
3062	142 - 151	599.3107	1196.6069	1196.6077	-0.67	0	26	0.0023	1	U	K.YILSPNFSEK.A
3063	142 - 151	599.3109	1196.6073	1196.6077	-0.37	0	14	0.043	1	U	K.YILSPNFSEK.A
3064	142 - 151	599.3109	1196.6073	1196.6077	-0.37	0	21	0.0081	1	U	K.YILSPNFSEK.A
3065	142 - 151	599.3110	1196.6074	1196.6077	-0.26	0	18	0.016	1	U	K.YILSPNFSEK.A
3066	142 - 151	599.3110	1196.6074	1196.6077	-0.26	0	23	0.0051	1	U	K.YILSPNFSEK.A
3067	142 - 151	599.3110	1196.6075	1196.6077	-0.16	0	25	0.0035	1	U	K.YILSPNFSEK.A
3068	142 - 151	599.3111	1196.6076	1196.6077	-0.060	0	19	0.013	1	U	K.YILSPNFSEK.A
3069	142 - 151	599.3112	1196.6078	1196.6077	0.042	0	22	0.0068	1	U	K.YILSPNFSEK.A
3070	142 - 151	599.3113	1196.6080	1196.6077	0.25	0	22	0.0059	1	U	K.YILSPNFSEK.A
3071	142 - 151	599.3113	1196.6081	1196.6077	0.35	0	27	0.0022	1	U	K.YILSPNFSEK.A
3072	142 - 151	599.3113	1196.6081	1196.6077	0.35	0	24	0.004	1	U	K.YILSPNFSEK.A

Query	Start - End	Observed	Mr(expt)	Mr(calc)	ppm	M	Score	Expect	Rank	U	Peptide
3073	142 - 151	599.3113	1196.6081	1196.6077	0.35	0	28	0.0016	1	U	K.YILSPNFSEK.A
3074	142 - 151	599.3114	1196.6083	1196.6077	0.45	0	24	0.0038	1	U	K.YILSPNFSEK.A
3075	142 - 151	599.3115	1196.6084	1196.6077	0.55	0	27	0.0018	1	U	K.YILSPNFSEK.A
3076	142 - 151	599.3115	1196.6084	1196.6077	0.55	0	36	0.00023	1	U	K.YILSPNFSEK.A
3077	142 - 151	599.3115	1196.6084	1196.6077	0.55	0	15	0.03	1	U	K.YILSPNFSEK.A
3078	142 - 151	599.3115	1196.6084	1196.6077	0.55	0	35	0.00035	1	U	K.YILSPNFSEK.A
3079	142 - 151	599.3115	1196.6085	1196.6077	0.65	0	11	0.087	1	U	K.YILSPNFSEK.A
3080	142 - 151	599.3115	1196.6085	1196.6077	0.65	0	19	0.014	1	U	K.YILSPNFSEK.A
3081	142 - 151	599.3116	1196.6086	1196.6077	0.76	0	25	0.0034	1	U	K.YILSPNFSEK.A
3082	142 - 151	599.3116	1196.6087	1196.6077	0.86	0	30	0.00098	1	U	K.YILSPNFSEK.A
3083	142 - 151	599.3116	1196.6087	1196.6077	0.86	0	32	0.00062	1	U	K.YILSPNFSEK.A
3084	142 - 151	599.3117	1196.6089	1196.6077	0.96	0	15	0.031	1	U	K.YILSPNFSEK.A
3085	142 - 151	599.3118	1196.6090	1196.6077	1.06	0	18	0.017	1	U	K.YILSPNFSEK.A
3086	142 - 151	599.3118	1196.6090	1196.6077	1.06	0	28	0.0015	1	U	K.YILSPNFSEK.A
3087	142 - 151	599.3118	1196.6090	1196.6077	1.06	0	20	0.009	1	U	K.YILSPNFSEK.A
3088	142 - 151	599.3118	1196.6091	1196.6077	1.16	0	21	0.0087	1	U	K.YILSPNFSEK.A
3089	142 - 151	599.3118	1196.6091	1196.6077	1.16	0	27	0.002	1	U	K.YILSPNFSEK.A
3090	142 - 151	599.3118	1196.6091	1196.6077	1.16	0	22	0.0065	1	U	K.YILSPNFSEK.A
3091	142 - 151	599.3118	1196.6091	1196.6077	1.16	0	27	0.0022	1	U	K.YILSPNFSEK.A
3092	142 - 151	599.3119	1196.6092	1196.6077	1.27	0	15	0.031	1	U	K.YILSPNFSEK.A
3093	142 - 151	599.3120	1196.6095	1196.6077	1.47	0	22	0.0061	1	U	K.YILSPNFSEK.A
3094	142 - 151	599.3121	1196.6097	1196.6077	1.67	0	8	0.16	1	U	K.YILSPNFSEK.A
3095	142 - 151	599.3123	1196.6100	1196.6077	1.88	0	38	0.00014	1	U	K.YILSPNFSEK.A
3096	142 - 151	599.3123	1196.6100	1196.6077	1.88	0	6	0.25	1	U	K.YILSPNFSEK.A
3097	142 - 151	599.3123	1196.6100	1196.6077	1.88	0	39	0.00012	1	U	K.YILSPNFSEK.A
3098	142 - 151	599.3127	1196.6109	1196.6077	2.69	0	18	0.016	1	U	K.YILSPNFSEK.A
3099	142 - 151	599.3128	1196.6111	1196.6077	2.80	0	9	0.14	1	U	K.YILSPNFSEK.A
6902	142 - 158	637.6666	1909.9781	1909.9785	-0.23	1	1	0.83	1	U	K.YILSPNFSEKANSLAEK.E
520	152 - 158	366.6978	731.3811	731.3813	-0.27	0	45	3e-005	1	U	K.ANSLAEK.E
521	152 - 158	366.6979	731.3813	731.3813	-0.098	0	46	2.3e-005	1	U	K.ANSLAEK.E
522	152 - 158	366.6979	731.3813	731.3813	-0.098	0	52	7e-006	1	U	K.ANSLAEK.E
523	152 - 158	366.6980	731.3814	731.3813	0.071	0	36	0.00025	1	U	K.ANSLAEK.E
10476	152 - 172	831.0660	2490.1761	2490.1815	-2.17	1	40	9e-005	1	U	K.ANSLAEKETQSFFPNYNLFDR.Q
10477	152 - 172	831.0661	2490.1765	2490.1815	-2.03	1	19	0.012	1	U	K.ANSLAEKETQSFFPNYNLFDR.Q
10478	152 - 172	831.0666	2490.1779	2490.1815	-1.44	1	22	0.0063	1	U	K.ANSLAEKETQSFFPNYNLFDR.Q
10479	152 - 172	831.0670	2490.1792	2490.1815	-0.92	1	28	0.0016	1	U	K.ANSLAEKETQSFFPNYNLFDR.Q
10480	152 - 172	831.0673	2490.1801	2490.1815	-0.55	1	24	0.0038	1	U	K.ANSLAEKETQSFFPNYNLFDR.Q
10481	152 - 172	831.0676	2490.1809	2490.1815	-0.26	1	16	0.026	1	U	K.ANSLAEKETQSFFPNYNLFDR.Q
10482	152 - 172	831.0682	2490.1829	2490.1815	0.55	1	3	0.53	1	U	K.ANSLAEKETQSFFPNYNLFDR.Q
10483	152 - 172	831.0684	2490.1834	2490.1815	0.77	1	43	5.6e-005	1	U	K.ANSLAEKETQSFFPNYNLFDR.Q
10484	152 - 172	1246.0994	2490.1842	2490.1815	1.07	1	66	2.5e-007	1	U	K.ANSLAEKETQSFFPNYNLFDR.Q
10485	152 - 172	623.5534	2490.1845	2490.1815	1.21	1	33	0.00054	1	U	K.ANSLAEKETQSFFPNYNLFDR.Q
14262	152 - 179	826.8949	3303.5505	3303.5432	2.21	2	5	0.29	1	U	K.ANSLAEKETQSFFPNYNLFDRQQENGEK.Q
6163	159 - 172	889.4113	1776.8081	1776.8107	-1.50	0	15	0.035	1	U	K.ETQSFFPNYNLFDR.Q
6164	159 - 172	889.4118	1776.8091	1776.8107	-0.95	0	28	0.0018	1	U	K.ETQSFFPNYNLFDR.Q
6165	159 - 172	889.4122	1776.8098	1776.8107	-0.54	0	17	0.019	1	U	K.ETQSFFPNYNLFDR.Q
6166	159 - 172	889.4124	1776.8102	1776.8107	-0.33	0	0	0.98	1	U	K.ETQSFFPNYNLFDR.Q
6167	159 - 172	889.4124	1776.8103	1776.8107	-0.26	0	6	0.24	1	U	K.ETQSFFPNYNLFDR.Q
6168	159 - 172	889.4124	1776.8103	1776.8107	-0.26	0	20	0.01	1	U	K.ETQSFFPNYNLFDR.Q
6169	159 - 172	889.4126	1776.8106	1776.8107	-0.057	0	19	0.012	1	U	K.ETQSFFPNYNLFDR.Q
6170	159 - 172	889.4127	1776.8108	1776.8107	0.012	0	9	0.14	1	U	K.ETQSFFPNYNLFDR.Q
6171	159 - 172	889.4127	1776.8109	1776.8107	0.080	0	3	0.46	1	U	K.ETQSFFPNYNLFDR.Q
6173	159 - 172	889.4127	1776.8109	1776.8107	0.080	0	29	0.0014	1	U	K.ETQSFFPNYNLFDR.Q
6174	159 - 172	889.4128	1776.8110	1776.8107	0.15	0	15	0.035	1	U	K.ETQSFFPNYNLFDR.Q
6175	159 - 172	889.4128	1776.8111	1776.8107	0.22	0	13	0.046	1	U	K.ETQSFFPNYNLFDR.Q
6176	159 - 172	889.4130	1776.8114	1776.8107	0.36	0	7	0.22	1	U	K.ETQSFFPNYNLFDR.Q
6177	159 - 172	889.4130	1776.8115	1776.8107	0.42	0	8	0.16	1	U	K.ETQSFFPNYNLFDR.Q
6178	159 - 172	889.4130	1776.8115	1776.8107	0.42	0	50	9.1e-006	1	U	K.ETQSFFPNYNLFDR.Q
6179	159 - 172	889.4131	1776.8116	1776.8107	0.49	0	18	0.015	1	U	K.ETQSFFPNYNLFDR.Q
6180	159 - 172	889.4132	1776.8119	1776.8107	0.63	0	10	0.093	1	U	K.ETQSFFPNYNLFDR.Q
6181	159 - 172	889.4132	1776.8119	1776.8107	0.63	0	20	0.01	1	U	K.ETQSFFPNYNLFDR.Q
6184	159 - 172	889.4133	1776.8121	1776.8107	0.77	0	45	3.2e-005	1	U	K.ETQSFFPNYNLFDR.Q
6185	159 - 172	889.4133	1776.8121	1776.8107	0.77	0	21	0.0076	1	U	K.ETQSFFPNYNLFDR.Q
6186	159 - 172	889.4134	1776.8122	1776.8107	0.84	0	12	0.061	1	U	K.ETQSFFPNYNLFDR.Q
6187	159 - 172	889.4134	1776.8122	1776.8107	0.84	0	9	0.12	1	U	K.ETQSFFPNYNLFDR.Q
6188	159 - 172	889.4134	1776.8122	1776.8107	0.84	0	49	1.2e-005	1	U	K.ETQSFFPNYNLFDR.Q
6189	159 - 172	889.4135	1776.8124	1776.8107	0.90	0	19	0.012	1	U	K.ETQSFFPNYNLFDR.Q
6190	159 - 172	593.2781	1776.8124	1776.8107	0.93	0	30	0.00091	1	U	K.ETQSFFPNYNLFDR.Q
6191	159 - 172	889.4135	1776.8125	1776.8107	0.97	0	27	0.0018	1	U	K.ETQSFFPNYNLFDR.Q
6192	159 - 172	593.2781	1776.8126	1776.8107	1.03	0	30	0.00093	1	U	K.ETQSFFPNYNLFDR.Q
6193	159 - 172	889.4138	1776.8130	1776.8107	1.25	0	24	0.0042	1	U	K.ETQSFFPNYNLFDR.Q
6194	159 - 172	889.4138	1776.8131	1776.8107	1.32	0	6	0.27	1	U	K.ETQSFFPNYNLFDR.Q
6195	159 - 172	889.4139	1776.8132	1776.8107	1.39	0	5	0.32	1	U	K.ETQSFFPNYNLFDR.Q
6196	159 - 172	889.4139	1776.8133	1776.8107	1.45	0	15	0.03	1	U	K.ETQSFFPNYNLFDR.Q
6197	159 - 172	889.4142	1776.8139	1776.8107	1.80	0	11	0.073	1	U	K.ETQSFFPNYNLFDR.Q
6198	159 - 172	889.4142	1776.8139	1776.8107	1.80	0	22	0.0063	1	U	K.ETQSFFPNYNLFDR.Q
6199	159 - 172	889.4144	1776.8142	1776.8107	1.94	0	14	0.044	1	U	K.ETQSFFPNYNLFDR.Q
6200	159 - 172	889.4145	1776.8144	1776.8107	2.07	0	0	0.91	1	U	K.ETQSFFPNYNLFDR.Q
11102	159 - 179	864.3996	2590.1770	2590.1724	1.75	1	14	0.041	1	U	K.ETQSFFPNYNLFDRQQENGEK.Q
10508	180 - 200	832.6792	2495.0158	2495.0105	2.13	0	34	0.00036	1	U	K.QSMSTPVDTDNDCIPDEWEEK.G
10509	180 - 200	1248.5178	2495.0211	2495.0105	4.26	0	44	3.7e-005	1	U	K.QSMSTPVDTDNDCIPDEWEEK.G
10574	180 - 200	838.0078	2511.0014	2511.0054	-1.57	0	11	0.081	1	U	K.QSMSTPVDTDNDCIPDEWEEK.G + Oxidation (M)
10575	180 - 200	1256.5110	2511.0074	2511.0054	0.82	0	47	2.2e-005	1	U	K.QSMSTPVDTDNDCIPDEWEEK.G + Oxidation (M)
10576	180 - 200	1256.5139	2511.0133	2511.0054	3.15	0	72	6.6e-008	1	U	K.QSMSTPVDTDNDCIPDEWEEK.G + Oxidation (M)
10577	180 - 200	838.0128	2511.0164	2511.0054	4.41	0	24	0.0043	1	U	K.QSMSTPVDTDNDCIPDEWEEK.G + Oxidation (M)
13344	180 - 205	1040.7798	3119.3175	3119.3125	1.63	1	45	3.3e-005	1	U	K.QSMSTPVDTDNDCIPDEWEEKGYTFR.N
13376	180 - 205	1046.1083	3135.3030	3135.3074	-1.39	1	68	1.6e-007	1	U	K.QSMSTPVDTDNDCIPDEWEEKGYTFR.N + Oxidation (M)
13377	180 - 205	784.8346	3135.3093	3135.3074	0.61	1	42	6e-005	1	U	K.QSMSTPVDTDNDCIPDEWEEKGYTFR.N + Oxidation (M)

Query	Start - End	Observed	Mr(expt)	Mr(calc)	ppm	M	Score	Expect	Rank	U	Peptide
290	201 - 205	322.1635	642.3125	642.3126	-0.14	0	8	0.15	1	U	K.GYTFR.N
291	201 - 205	322.1636	642.3127	642.3126	0.15	0	8	0.17	1	U	K.GYTFR.N
7641	206 - 222	661.6433	1981.9081	1981.9170	-4.48	0	48	1.4e-005	1	U	R.NQQIVPWNDAYSAEGYK.K
7642	206 - 222	991.9675	1981.9204	1981.9170	1.72	0	54	4.2e-006	1	U	R.NQQIVPWNDAYSAEGYK.K
7643	206 - 222	991.9675	1981.9204	1981.9170	1.72	0	26	0.0025	1	U	R.NQQIVPWNDAYSAEGYK.K
8565	206 - 223	1056.0083	2110.0021	2110.0119	-4.68	1	85	3.5e-009	1	U	R.NQQIVPWNDAYSAEGYKK.Y
8566	206 - 223	528.5078	2110.0021	2110.0119	-4.64	1	13	0.046	1	U	R.NQQIVPWNDAYSAEGYKK.Y
8567	206 - 223	704.3427	2110.0063	2110.0119	-2.67	1	9	0.14	1	U	R.NQQIVPWNDAYSAEGYKK.Y
8568	206 - 223	704.3431	2110.0076	2110.0119	-2.06	1	21	0.0076	1	U	R.NQQIVPWNDAYSAEGYKK.Y
8569	206 - 223	704.3437	2110.0092	2110.0119	-1.28	1	10	0.097	1	U	R.NQQIVPWNDAYSAEGYKK.Y
8571	206 - 223	704.3444	2110.0113	2110.0119	-0.32	1	11	0.089	1	U	R.NQQIVPWNDAYSAEGYKK.Y
8573	206 - 223	704.3445	2110.0116	2110.0119	-0.15	1	12	0.06	1	U	R.NQQIVPWNDAYSAEGYKK.Y
8574	206 - 223	704.3446	2110.0120	2110.0119	0.025	1	10	0.096	1	U	R.NQQIVPWNDAYSAEGYKK.Y
8575	206 - 223	704.3447	2110.0124	2110.0119	0.20	1	14	0.039	1	U	R.NQQIVPWNDAYSAEGYKK.Y
8576	206 - 223	704.3448	2110.0125	2110.0119	0.29	1	23	0.0052	1	U	R.NQQIVPWNDAYSAEGYKK.Y
8577	206 - 223	704.3448	2110.0127	2110.0119	0.37	1	27	0.0021	1	U	R.NQQIVPWNDAYSAEGYKK.Y
8578	206 - 223	704.3449	2110.0129	2110.0119	0.46	1	17	0.018	1	U	R.NQQIVPWNDAYSAEGYKK.Y
8579	206 - 223	704.3450	2110.0131	2110.0119	0.55	1	26	0.0023	1	U	R.NQQIVPWNDAYSAEGYKK.Y
8580	206 - 223	704.3450	2110.0133	2110.0119	0.63	1	13	0.055	1	U	R.NQQIVPWNDAYSAEGYKK.Y
8581	206 - 223	704.3452	2110.0136	2110.0119	0.81	1	9	0.12	1	U	R.NQQIVPWNDAYSAEGYKK.Y
8582	206 - 223	704.3453	2110.0142	2110.0119	1.07	1	4	0.43	1	U	R.NQQIVPWNDAYSAEGYKK.Y
8583	206 - 223	1056.0144	2110.0143	2110.0119	1.10	1	46	2.6e-005	1	U	R.NQQIVPWNDAYSAEGYKK.Y
8584	206 - 223	704.3455	2110.0145	2110.0119	1.24	1	1	0.84	1	U	R.NQQIVPWNDAYSAEGYKK.Y
8585	206 - 223	704.3456	2110.0149	2110.0119	1.41	1	1	0.71	1	U	R.NQQIVPWNDAYSAEGYKK.Y
8586	206 - 223	704.3456	2110.0149	2110.0119	1.41	1	8	0.15	1	U	R.NQQIVPWNDAYSAEGYKK.Y
8588	206 - 223	704.3458	2110.0155	2110.0119	1.67	1	0	0.97	1	U	R.NQQIVPWNDAYSAEGYKK.Y
8591	206 - 223	704.3460	2110.0162	2110.0119	2.02	1	12	0.062	1	U	R.NQQIVPWNDAYSAEGYKK.Y
8592	206 - 223	704.3461	2110.0164	2110.0119	2.11	1	12	0.066	1	U	R.NQQIVPWNDAYSAEGYKK.Y
8593	206 - 223	704.3462	2110.0167	2110.0119	2.28	1	4	0.38	1	U	R.NQQIVPWNDAYSAEGYKK.Y
8594	206 - 223	704.3467	2110.0182	2110.0119	2.98	1	5	0.31	1	U	R.NQQIVPWNDAYSAEGYKK.Y
8596	206 - 223	704.3473	2110.0200	2110.0119	3.84	1	14	0.038	1	U	R.NQQIVPWNDAYSAEGYKK.Y
3449	223 - 232	412.2157	1233.6252	1233.6254	-0.20	1	38	0.00018	1	U	K.KYVSNPYHAR.T
3450	223 - 232	412.2157	1233.6253	1233.6254	-0.13	1	39	0.00014	1	U	K.KYVSNPYHAR.T
3451	223 - 232	309.4138	1233.6260	1233.6254	0.50	1	14	0.039	1	U	K.KYVSNPYHAR.T
3452	223 - 232	617.8206	1233.6267	1233.6254	1.02	1	33	0.00045	1	U	K.KYVSNPYHAR.T
2542	224 - 232	553.7720	1105.5294	1105.5305	-0.98	0	23	0.0053	1	U	K.YVSNPYHAR.T
2543	224 - 232	553.7721	1105.5296	1105.5305	-0.75	0	24	0.0041	1	U	K.YVSNPYHAR.T
2544	224 - 232	553.7722	1105.5298	1105.5305	-0.64	0	26	0.0027	1	U	K.YVSNPYHAR.T
2545	224 - 232	553.7723	1105.5301	1105.5305	-0.31	0	32	0.00057	1	U	K.YVSNPYHAR.T
2546	224 - 232	369.5174	1105.5304	1105.5305	-0.10	0	17	0.02	1	U	K.YVSNPYHAR.T
2547	224 - 232	553.7725	1105.5304	1105.5305	-0.092	0	41	8.7e-005	1	U	K.YVSNPYHAR.T
2548	224 - 232	369.5174	1105.5305	1105.5305	-0.020	0	29	0.0012	1	U	K.YVSNPYHAR.T
2549	224 - 232	369.5174	1105.5305	1105.5305	-0.020	0	29	0.0014	1	U	K.YVSNPYHAR.T
2550	224 - 232	553.7726	1105.5306	1105.5305	0.13	0	22	0.0062	1	U	K.YVSNPYHAR.T
2551	224 - 232	369.5175	1105.5306	1105.5305	0.15	0	30	0.00093	1	U	K.YVSNPYHAR.T
2552	224 - 232	369.5176	1105.5309	1105.5305	0.39	0	26	0.0024	1	U	K.YVSNPYHAR.T
2553	224 - 232	553.7730	1105.5315	1105.5305	0.90	0	35	0.00032	1	U	K.YVSNPYHAR.T
2554	224 - 232	553.7736	1105.5327	1105.5305	2.01	0	20	0.0095	1	U	K.YVSNPYHAR.T
4030	233 - 243	671.8283	1341.6421	1341.6452	-2.37	1	29	0.0012	1	U	R.TVKDPYTDFEK.V
4031	233 - 243	448.2214	1341.6424	1341.6452	-2.13	1	29	0.0012	1	U	R.TVKDPYTDFEK.V
4032	233 - 243	671.8287	1341.6428	1341.6452	-1.83	1	10	0.099	1	U	R.TVKDPYTDFEK.V
4033	233 - 243	671.8292	1341.6438	1341.6452	-1.10	1	9	0.14	1	U	R.TVKDPYTDFEK.V
4034	233 - 243	448.2220	1341.6442	1341.6452	-0.77	1	23	0.0047	1	U	R.TVKDPYTDFEK.V
4035	233 - 243	448.2220	1341.6443	1341.6452	-0.70	1	20	0.01	1	U	R.TVKDPYTDFEK.V
4036	233 - 243	448.2221	1341.6446	1341.6452	-0.50	1	27	0.0022	1	U	R.TVKDPYTDFEK.V
4037	233 - 243	448.2222	1341.6448	1341.6452	-0.36	1	26	0.0025	1	U	R.TVKDPYTDFEK.V
4038	233 - 243	448.2222	1341.6449	1341.6452	-0.29	1	29	0.0014	1	U	R.TVKDPYTDFEK.V
4039	233 - 243	448.2224	1341.6452	1341.6452	-0.017	1	5	0.3	1	U	R.TVKDPYTDFEK.V
4040	233 - 243	671.8301	1341.6456	1341.6452	0.27	1	19	0.012	1	U	R.TVKDPYTDFEK.V
4041	233 - 243	448.2225	1341.6457	1341.6452	0.32	1	25	0.0029	1	U	R.TVKDPYTDFEK.V
4042	233 - 243	448.2225	1341.6458	1341.6452	0.39	1	29	0.0014	1	U	R.TVKDPYTDFEK.V
4043	233 - 243	448.2226	1341.6461	1341.6452	0.60	1	21	0.0079	1	U	R.TVKDPYTDFEK.V
4044	233 - 243	448.2227	1341.6463	1341.6452	0.80	1	29	0.0013	1	U	R.TVKDPYTDFEK.V
4045	233 - 243	448.2228	1341.6466	1341.6452	1.01	1	28	0.0016	1	U	R.TVKDPYTDFEK.V
4046	233 - 243	448.2229	1341.6469	1341.6452	1.21	1	21	0.0087	1	U	R.TVKDPYTDFEK.V
4047	233 - 243	671.8308	1341.6471	1341.6452	1.36	1	20	0.01	1	U	R.TVKDPYTDFEK.V
9516	233 - 253	584.7938	2335.1459	2335.1518	-2.52	2	25	0.0029	1	U	R.TVKDPYTDFEKVTGHMPAATK.Y
9594	233 - 253	588.7936	2351.1452	2351.1468	-0.66	2	26	0.0027	1	U	R.TVKDPYTDFEKVTGHMPAATK.Y + Oxidation (M)
9595	233 - 253	784.7224	2351.1454	2351.1468	-0.57	2	33	0.00051	1	U	R.TVKDPYTDFEKVTGHMPAATK.Y + Oxidation (M)
9596	233 - 253	471.2367	2351.1471	2351.1468	0.14	2	14	0.039	1	U	R.TVKDPYTDFEKVTGHMPAATK.Y + Oxidation (M)
2028	236 - 243	507.7238	1013.4330	1013.4342	-1.16	0	4	0.37	1	U	K.DPYTDFEK.V
2029	236 - 243	507.7240	1013.4335	1013.4342	-0.67	0	15	0.033	1	U	K.DPYTDFEK.V
2031	236 - 243	507.7244	1013.4343	1013.4342	0.11	0	26	0.0024	1	U	K.DPYTDFEK.V
2032	236 - 243	507.7245	1013.4344	1013.4342	0.23	0	33	0.00048	1	U	K.DPYTDFEK.V
2033	236 - 243	507.7245	1013.4345	1013.4342	0.35	0	8	0.15	1	U	K.DPYTDFEK.V
7961	236 - 253	675.3193	2022.9362	2022.9357	0.24	1	43	4.9e-005	1	U	K.DPYTDFEKVTGHMPAATK.Y + Oxidation (M)
7962	236 - 253	506.7417	2022.9377	2022.9357	0.99	1	29	0.0012	1	U	K.DPYTDFEKVTGHMPAATK.Y + Oxidation (M)
2018	244 - 253	506.7657	1011.5169	1011.5172	-0.27	0	27	0.0019	1	U	K.VTGHMPAATK.Y
2019	244 - 253	506.7660	1011.5174	1011.5172	0.28	0	52	6.2e-006	1	U	K.VTGHMPAATK.Y
2020	244 - 253	338.1810	1011.5212	1011.5172	3.98	0	25	0.0031	1	U	K.VTGHMPAATK.Y
2128	244 - 253	343.5111	1027.5115	1027.5121	-0.56	0	18	0.016	1	U	K.VTGHMPAATK.Y + Oxidation (M)
2129	244 - 253	343.5112	1027.5119	1027.5121	-0.20	0	37	0.0002	1	U	K.VTGHMPAATK.Y + Oxidation (M)
2130	244 - 253	343.5112	1027.5119	1027.5121	-0.20	0	37	0.00019	1	U	K.VTGHMPAATK.Y + Oxidation (M)
2131	244 - 253	514.7632	1027.5119	1027.5121	-0.13	0	23	0.0049	1	U	K.VTGHMPAATK.Y + Oxidation (M)
2132	244 - 253	514.7632	1027.5119	1027.5121	-0.13	0	43	5.5e-005	1	U	K.VTGHMPAATK.Y + Oxidation (M)
2133	244 - 253	343.5113	1027.5120	1027.5121	-0.11	0	27	0.0018	1	U	K.VTGHMPAATK.Y + Oxidation (M)
2134	244 - 253	514.7634	1027.5122	1027.5121	0.11	0	20	0.0091	1	U	K.VTGHMPAATK.Y + Oxidation (M)
2135	244 - 253	514.7635	1027.5124	1027.5121	0.35	0	43	4.6e-005	1	U	K.VTGHMPAATK.Y + Oxidation (M)

Query	Start – End	Observed	Mr(expt)	Mr(calc)	ppm	M	Score	Expect	Rank	U	Peptide
2136	244 – 253	514.7635	1027.5124	1027.5121	0.35	0	51	8.6e-006	1	U	K.VTGHMPAATK.Y + Oxidation (M)
4876	244 – 257	511.2606	1530.7598	1530.7613	-0.93	1	19	0.011	1	U	K.VTGHMPAATKYEAR.D
4936	244 – 257	516.5924	1546.7554	1546.7562	-0.52	1	51	7.8e-006	1	U	K.VTGHMPAATKYEAR.D + Oxidation (M)
4937	244 – 257	387.6961	1546.7554	1546.7562	-0.49	1	30	0.0011	1	U	K.VTGHMPAATKYEAR.D + Oxidation (M)
4938	244 – 257	774.3857	1546.7569	1546.7562	0.48	1	27	0.002	1	U	K.VTGHMPAATKYEAR.D + Oxidation (M)
13633	244 – 273	795.1485	3176.5649	3176.5634	0.46	2	22	0.0062	1	U	K.VTGHMPAATKYEARDPLVAAYPSVGVGMEK.L + 2 Oxidation (M)
96	254 – 257	269.6345	537.2545	537.2547	-0.36	0	15	0.031	1	U	K.YEAR.D
8802	254 – 273	718.0309	2151.0708	2151.0670	1.77	1	52	6.7e-006	1	U	K.YEARDPLVAAYPSVGVGMEK.L
8803	254 – 273	718.0316	2151.0728	2151.0670	2.71	1	29	0.0012	1	U	K.YEARDPLVAAYPSVGVGMEK.L
8804	254 – 273	1076.5449	2151.0753	2151.0670	3.85	1	61	8.5e-007	1	U	K.YEARDPLVAAYPSVGVGMEK.L
8849	254 – 273	723.3608	2167.0605	2167.0619	-0.66	1	6	0.26	1	U	K.YEARDPLVAAYPSVGVGMEK.L + Oxidation (M)
8850	254 – 273	723.3610	2167.0612	2167.0619	-0.32	1	5	0.33	1	U	K.YEARDPLVAAYPSVGVGMEK.L + Oxidation (M)
8851	254 – 273	723.3611	2167.0616	2167.0619	-0.15	1	2	0.65	1	U	K.YEARDPLVAAYPSVGVGMEK.L + Oxidation (M)
8852	254 – 273	723.3613	2167.0620	2167.0619	0.018	1	6	0.28	1	U	K.YEARDPLVAAYPSVGVGMEK.L + Oxidation (M)
8853	254 – 273	723.3616	2167.0631	2167.0619	0.52	1	5	0.32	1	U	K.YEARDPLVAAYPSVGVGMEK.L + Oxidation (M)
8854	254 – 273	723.3617	2167.0633	2167.0619	0.61	1	38	0.00015	1	U	K.YEARDPLVAAYPSVGVGMEK.L + Oxidation (M)
8855	254 – 273	723.3619	2167.0638	2167.0619	0.86	1	20	0.0099	1	U	K.YEARDPLVAAYPSVGVGMEK.L + Oxidation (M)
8856	254 – 273	723.3622	2167.0649	2167.0619	1.37	1	9	0.13	1	U	K.YEARDPLVAAYPSVGVGMEK.L + Oxidation (M)
8857	254 – 273	1084.5398	2167.0650	2167.0619	1.43	1	44	3.6e-005	1	U	K.YEARDPLVAAYPSVGVGMEK.L + Oxidation (M)
8858	254 – 273	723.3623	2167.0651	2167.0619	1.45	1	22	0.0069	1	U	K.YEARDPLVAAYPSVGVGMEK.L + Oxidation (M)
8859	254 – 273	723.3633	2167.0682	2167.0619	2.89	1	47	1.8e-005	1	U	K.YEARDPLVAAYPSVGVGMEK.L + Oxidation (M)
8860	254 – 273	723.3637	2167.0693	2167.0619	3.40	1	18	0.016	1	U	K.YEARDPLVAAYPSVGVGMEK.L + Oxidation (M)
8861	254 – 273	723.3643	2167.0709	2167.0619	4.16	1	35	0.00033	1	U	K.YEARDPLVAAYPSVGVGMEK.L + Oxidation (M)
8862	254 – 273	1084.5430	2167.0714	2167.0619	4.36	1	12	0.069	1	U	K.YEARDPLVAAYPSVGVGMEK.L + Oxidation (M)
8863	254 – 273	1084.5435	2167.0724	2167.0619	4.81	1	34	0.00043	1	U	K.YEARDPLVAAYPSVGVGMEK.L + Oxidation (M)
5383	258 – 273	544.9485	1631.8236	1631.8229	0.45	0	53	5.4e-006	1	U	R.DPLVAAYPSVGVGMEK.L
5384	258 – 273	816.9211	1631.8277	1631.8229	2.97	0	46	2.2e-005	1	U	R.DPLVAAYPSVGVGMEK.L
5428	258 – 273	824.9151	1647.8156	1647.8178	-1.31	0	17	0.022	1	U	R.DPLVAAYPSVGVGMEK.L + Oxidation (M)
5429	258 – 273	824.9155	1647.8165	1647.8178	-0.79	0	14	0.039	1	U	R.DPLVAAYPSVGVGMEK.L + Oxidation (M)
5430	258 – 273	824.9156	1647.8167	1647.8178	-0.65	0	59	1.4e-006	1	U	R.DPLVAAYPSVGVGMEK.L + Oxidation (M)
5431	258 – 273	824.9156	1647.8167	1647.8178	-0.65	0	75	3.2e-008	1	U	R.DPLVAAYPSVGVGMEK.L + Oxidation (M)
5432	258 – 273	824.9157	1647.8169	1647.8178	-0.57	0	10	0.099	1	U	R.DPLVAAYPSVGVGMEK.L + Oxidation (M)
5433	258 – 273	824.9157	1647.8169	1647.8178	-0.57	0	24	0.0044	1	U	R.DPLVAAYPSVGVGMEK.L + Oxidation (M)
5434	258 – 273	824.9158	1647.8170	1647.8178	-0.50	0	12	0.064	1	U	R.DPLVAAYPSVGVGMEK.L + Oxidation (M)
5435	258 – 273	824.9159	1647.8172	1647.8178	-0.35	0	63	5.5e-007	1	U	R.DPLVAAYPSVGVGMEK.L + Oxidation (M)
5436	258 – 273	824.9162	1647.8178	1647.8178	0.022	0	14	0.036	1	U	R.DPLVAAYPSVGVGMEK.L + Oxidation (M)
5437	258 – 273	550.2799	1647.8179	1647.8178	0.050	0	28	0.0015	1	U	R.DPLVAAYPSVGVGMEK.L + Oxidation (M)
5438	258 – 273	824.9163	1647.8181	1647.8178	0.17	0	37	0.0002	1	U	R.DPLVAAYPSVGVGMEK.L + Oxidation (M)
5439	258 – 273	824.9164	1647.8182	1647.8178	0.24	0	13	0.054	1	U	R.DPLVAAYPSVGVGMEK.L + Oxidation (M)
5440	258 – 273	824.9165	1647.8185	1647.8178	0.39	0	14	0.036	1	U	R.DPLVAAYPSVGVGMEK.L + Oxidation (M)
5441	258 – 273	824.9169	1647.8193	1647.8178	0.91	0	6	0.23	1	U	R.DPLVAAYPSVGVGMEK.L + Oxidation (M)
5442	258 – 273	550.2805	1647.8195	1647.8178	1.05	0	47	2e-005	1	U	R.DPLVAAYPSVGVGMEK.L + Oxidation (M)
5443	258 – 273	824.9171	1647.8196	1647.8178	1.06	0	15	0.035	1	U	R.DPLVAAYPSVGVGMEK.L + Oxidation (M)
5444	258 – 273	550.2805	1647.8197	1647.8178	1.16	0	44	4.1e-005	1	U	R.DPLVAAYPSVGVGMEK.L + Oxidation (M)
5445	258 – 273	824.9172	1647.8198	1647.8178	1.21	0	23	0.0054	1	U	R.DPLVAAYPSVGVGMEK.L + Oxidation (M)
5446	258 – 273	824.9172	1647.8199	1647.8178	1.28	0	12	0.067	1	U	R.DPLVAAYPSVGVGMEK.L + Oxidation (M)
262	274 – 278	316.1816	630.3487	630.3489	-0.43	0	28	0.0015	1	U	K.LHFSK.N
263	274 – 278	316.1817	630.3488	630.3489	-0.23	0	24	0.0042	1	U	K.LHFSK.N
264	274 – 278	316.1817	630.3488	630.3489	-0.23	0	23	0.0048	1	U	K.LHFSK.N
265	274 – 278	211.1235	630.3488	630.3489	-0.18	0	15	0.029	1	U	K.LHFSK.N
266	274 – 278	211.1236	630.3489	630.3489	-0.11	0	12	0.061	1	U	K.LHFSK.N
267	274 – 278	211.1236	630.3490	630.3489	0.11	0	11	0.077	1	U	K.LHFSK.N
268	274 – 278	211.1236	630.3491	630.3489	0.25	0	15	0.033	1	U	K.LHFSK.N
269	274 – 278	211.1238	630.3497	630.3489	1.20	0	11	0.087	1	U	K.LHFSK.N
6782	274 – 290	626.3066	1875.8979	1875.8963	0.87	1	36	0.00026	1	U	K.LHFSKNDTVTEGNADTK.S
6784	274 – 290	938.9568	1875.8991	1875.8963	1.53	1	64	3.6e-007	1	U	K.LHFSKNDTVTEGNADTK.S
6785	274 – 290	469.9822	1875.8999	1875.8963	1.91	1	28	0.0017	1	U	K.LHFSKNDTVTEGNADTK.S
3635	279 – 290	632.7855	1263.5564	1263.5579	-1.20	0	43	4.9e-005	1	U	K.NDTVTEGNADTK.S
3636	279 – 290	632.7863	1263.5581	1263.5579	0.15	0	73	4.7e-008	1	U	K.NDTVTEGNADTK.S
3637	279 – 290	632.7863	1263.5581	1263.5579	0.15	0	44	4e-005	1	U	K.NDTVTEGNADTK.S
3638	279 – 290	632.7864	1263.5582	1263.5579	0.25	0	58	1.6e-006	1	U	K.NDTVTEGNADTK.S
3639	279 – 290	632.7865	1263.5584	1263.5579	0.44	0	53	4.7e-006	1	U	K.NDTVTEGNADTK.S
3640	279 – 290	632.7865	1263.5584	1263.5579	0.44	0	26	0.0023	1	U	K.NDTVTEGNADTK.S
3641	279 – 290	632.7866	1263.5586	1263.5579	0.53	0	37	0.00022	1	U	K.NDTVTEGNADTK.S
3642	279 – 290	632.7866	1263.5586	1263.5579	0.53	0	54	3.8e-006	1	U	K.NDTVTEGNADTK.S
3643	279 – 290	422.1936	1263.5589	1263.5579	0.79	0	42	5.7e-005	1	U	K.NDTVTEGNADTK.S
3644	279 – 290	632.7868	1263.5591	1263.5579	0.92	0	48	1.4e-005	1	U	K.NDTVTEGNADTK.S
4682	279 – 292	740.3492	1478.6838	1478.6849	-0.72	1	32	0.00068	1	U	K.NDTVTEGNADTKSK.T
6901	279 – 296	637.6490	1909.9253	1909.9229	1.27	2	17	0.02	1	U	K.NDTVTEGNADTKSKTTTK.T
16	293 – 296	225.6315	449.2484	449.2486	-0.33	0	12	0.07	1	U	K.TTTK.T
17	293 – 296	225.6315	449.2485	449.2486	-0.12	0	13	0.053	1	U	K.TTTK.T
18	293 – 296	225.6316	449.2486	449.2486	0.080	0	11	0.083	1	U	K.TTTK.T
7142	297 – 315	971.9621	1941.9096	1941.9168	-3.67	0	110	1.1e-011	1	U	K.TDTTTNTVEIGGSLGFSDK.G
7143	297 – 315	648.3106	1941.9100	1941.9168	-3.48	0	55	2.9e-006	1	U	K.TDTTTNTVEIGGSLGFSDK.G
7144	297 – 315	971.9624	1941.9103	1941.9168	-3.35	0	106	2.3e-011	1	U	K.TDTTTNTVEIGGSLGFSDK.G
7145	297 – 315	971.9637	1941.9128	1941.9168	-2.03	0	86	2.7e-009	1	U	K.TDTTTNTVEIGGSLGFSDK.G
7146	297 – 315	648.3117	1941.9133	1941.9168	-1.79	0	35	0.00035	1	U	K.TDTTTNTVEIGGSLGFSDK.G
7147	297 – 315	971.9641	1941.9135	1941.9168	-1.66	0	95	3.2e-010	1	U	K.TDTTTNTVEIGGSLGFSDK.G
7148	297 – 315	648.3121	1941.9144	1941.9168	-1.22	0	38	0.00015	1	U	K.TDTTTNTVEIGGSLGFSDK.G
7149	297 – 315	971.9646	1941.9146	1941.9168	-1.09	0	128	1.5e-013	1	U	K.TDTTTNTVEIGGSLGFSDK.G
7150	297 – 315	971.9648	1941.9150	1941.9168	-0.90	0	97	1.9e-010	1	U	K.TDTTTNTVEIGGSLGFSDK.G
7151	297 – 315	648.3123	1941.9151	1941.9168	-0.84	0	53	5e-006	1	U	K.TDTTTNTVEIGGSLGFSDK.G
7152	297 – 315	648.3123	1941.9151	1941.9168	-0.84	0	57	2.1e-006	1	U	K.TDTTTNTVEIGGSLGFSDK.G
7153	297 – 315	971.9650	1941.9154	1941.9168	-0.71	0	109	1.1e-011	1	U	K.TDTTTNTVEIGGSLGFSDK.G
7154	297 – 315	648.3124	1941.9155	1941.9168	-0.66	0	50	1e-005	1	U	K.TDTTTNTVEIGGSLGFSDK.G
7155	297 – 315	648.3124	1941.9155	1941.9168	-0.66	0	58	1.4e-006	1	U	K.TDTTTNTVEIGGSLGFSDK.G
7156	297 – 315	648.3125	1941.9157	1941.9168	-0.56	0	51	7.1e-006	1	U	K.TDTTTNTVEIGGSLGFSDK.G

Query	Start - End	Observed	Mr(expt)	Mr(calc)	ppm	M	Score	Expect	Rank	U	Peptide
7157	297 - 315	648.3125	1941.9157	1941.9168	-0.56	0	38	0.00015	1	U	K.TDTTTNTVEIGGSLGFSDK.G
7158	297 - 315	648.3126	1941.9159	1941.9168	-0.47	0	37	0.00022	1	U	K.TDTTTNTVEIGGSLGFSDK.G
7159	297 - 315	971.9653	1941.9160	1941.9168	-0.40	0	83	4.9e-009	1	U	K.TDTTTNTVEIGGSLGFSDK.G
7160	297 - 315	648.3126	1941.9160	1941.9168	-0.37	0	36	0.00027	1	U	K.TDTTTNTVEIGGSLGFSDK.G
7161	297 - 315	971.9653	1941.9161	1941.9168	-0.34	0	83	5.2e-009	1	U	K.TDTTTNTVEIGGSLGFSDK.G
7162	297 - 315	971.9654	1941.9162	1941.9168	-0.27	0	85	3.4e-009	1	U	K.TDTTTNTVEIGGSLGFSDK.G
7163	297 - 315	971.9656	1941.9166	1941.9168	-0.084	0	90	1e-009	1	U	K.TDTTTNTVEIGGSLGFSDK.G
7164	297 - 315	971.9656	1941.9167	1941.9168	-0.021	0	75	2.9e-008	1	U	K.TDTTTNTVEIGGSLGFSDK.G
7165	297 - 315	971.9656	1941.9167	1941.9168	-0.021	0	75	2.9e-008	1	U	K.TDTTTNTVEIGGSLGFSDK.G
7166	297 - 315	648.3129	1941.9168	1941.9168	0.0036	0	49	1.3e-005	1	U	K.TDTTTNTVEIGGSLGFSDK.G
7167	297 - 315	648.3129	1941.9170	1941.9168	0.098	0	29	0.0012	1	U	K.TDTTTNTVEIGGSLGFSDK.G
7168	297 - 315	648.3129	1941.9170	1941.9168	0.098	0	43	4.6e-005	1	U	K.TDTTTNTVEIGGSLGFSDK.G
7169	297 - 315	648.3129	1941.9170	1941.9168	0.098	0	54	4.4e-006	1	U	K.TDTTTNTVEIGGSLGFSDK.G
7170	297 - 315	971.9658	1941.9170	1941.9168	0.10	0	102	5.7e-011	1	U	K.TDTTTNTVEIGGSLGFSDK.G
7171	297 - 315	971.9658	1941.9170	1941.9168	0.10	0	98	1.7e-010	1	U	K.TDTTTNTVEIGGSLGFSDK.G
7172	297 - 315	971.9658	1941.9170	1941.9168	0.10	0	94	4e-010	1	U	K.TDTTTNTVEIGGSLGFSDK.G
7173	297 - 315	971.9658	1941.9170	1941.9168	0.10	0	102	6.5e-011	1	U	K.TDTTTNTVEIGGSLGFSDK.G
7174	297 - 315	971.9658	1941.9170	1941.9168	0.10	0	109	1.2e-011	1	U	K.TDTTTNTVEIGGSLGFSDK.G
7175	297 - 315	971.9658	1941.9170	1941.9168	0.10	0	88	1.8e-009	1	U	K.TDTTTNTVEIGGSLGFSDK.G
7176	297 - 315	648.3130	1941.9171	1941.9168	0.19	0	53	4.9e-006	1	U	K.TDTTTNTVEIGGSLGFSDK.G
7177	297 - 315	971.9659	1941.9172	1941.9168	0.23	0	107	1.8e-011	1	U	K.TDTTTNTVEIGGSLGFSDK.G
7178	297 - 315	648.3130	1941.9173	1941.9168	0.29	0	53	4.7e-006	1	U	K.TDTTTNTVEIGGSLGFSDK.G
7179	297 - 315	648.3130	1941.9173	1941.9168	0.29	0	51	8.3e-006	1	U	K.TDTTTNTVEIGGSLGFSDK.G
7180	297 - 315	971.9659	1941.9173	1941.9168	0.29	0	96	2.4e-010	1	U	K.TDTTTNTVEIGGSLGFSDK.G
7181	297 - 315	971.9660	1941.9175	1941.9168	0.36	0	79	1.3e-008	1	U	K.TDTTTNTVEIGGSLGFSDK.G
7182	297 - 315	648.3131	1941.9175	1941.9168	0.38	0	29	0.0013	1	U	K.TDTTTNTVEIGGSLGFSDK.G
7183	297 - 315	648.3131	1941.9175	1941.9168	0.38	0	29	0.0012	1	U	K.TDTTTNTVEIGGSLGFSDK.G
7184	297 - 315	648.3131	1941.9175	1941.9168	0.38	0	54	4.1e-006	1	U	K.TDTTTNTVEIGGSLGFSDK.G
7185	297 - 315	648.3131	1941.9175	1941.9168	0.38	0	62	6.9e-007	1	U	K.TDTTTNTVEIGGSLGFSDK.G
7186	297 - 315	971.9661	1941.9176	1941.9168	0.42	0	90	1.1e-009	1	U	K.TDTTTNTVEIGGSLGFSDK.G
7187	297 - 315	971.9661	1941.9176	1941.9168	0.42	0	89	1.4e-009	1	U	K.TDTTTNTVEIGGSLGFSDK.G
7188	297 - 315	971.9661	1941.9176	1941.9168	0.42	0	108	1.7e-011	1	U	K.TDTTTNTVEIGGSLGFSDK.G
7189	297 - 315	971.9661	1941.9176	1941.9168	0.42	0	81	8.7e-009	1	U	K.TDTTTNTVEIGGSLGFSDK.G
7190	297 - 315	648.3132	1941.9177	1941.9168	0.47	0	48	1.4e-005	1	U	K.TDTTTNTVEIGGSLGFSDK.G
7191	297 - 315	648.3132	1941.9177	1941.9168	0.47	0	43	4.8e-005	1	U	K.TDTTTNTVEIGGSLGFSDK.G
7192	297 - 315	971.9661	1941.9177	1941.9168	0.48	0	94	4.4e-010	1	U	K.TDTTTNTVEIGGSLGFSDK.G
7193	297 - 315	971.9662	1941.9178	1941.9168	0.55	0	94	4.4e-010	1	U	K.TDTTTNTVEIGGSLGFSDK.G
7194	297 - 315	971.9662	1941.9179	1941.9168	0.61	0	115	3.5e-012	1	U	K.TDTTTNTVEIGGSLGFSDK.G
7195	297 - 315	971.9662	1941.9179	1941.9168	0.61	0	72	6.5e-008	1	U	K.TDTTTNTVEIGGSLGFSDK.G
7196	297 - 315	971.9662	1941.9179	1941.9168	0.61	0	83	4.7e-009	1	U	K.TDTTTNTVEIGGSLGFSDK.G
7197	297 - 315	648.3133	1941.9181	1941.9168	0.66	0	31	0.00085	1	U	K.TDTTTNTVEIGGSLGFSDK.G
7198	297 - 315	648.3133	1941.9181	1941.9168	0.66	0	55	3e-006	1	U	K.TDTTTNTVEIGGSLGFSDK.G
7199	297 - 315	971.9663	1941.9181	1941.9168	0.67	0	80	9.7e-009	1	U	K.TDTTTNTVEIGGSLGFSDK.G
7200	297 - 315	971.9663	1941.9181	1941.9168	0.67	0	85	3.3e-009	1	U	K.TDTTTNTVEIGGSLGFSDK.G
7201	297 - 315	971.9664	1941.9182	1941.9168	0.73	0	96	2.6e-010	1	U	K.TDTTTNTVEIGGSLGFSDK.G
7202	297 - 315	971.9664	1941.9183	1941.9168	0.80	0	87	2.1e-009	1	U	K.TDTTTNTVEIGGSLGFSDK.G
7203	297 - 315	971.9665	1941.9184	1941.9168	0.86	0	89	1.3e-009	1	U	K.TDTTTNTVEIGGSLGFSDK.G
7204	297 - 315	648.3135	1941.9186	1941.9168	0.95	0	47	1.8e-005	1	U	K.TDTTTNTVEIGGSLGFSDK.G
7205	297 - 315	648.3135	1941.9186	1941.9168	0.95	0	49	1.1e-005	1	U	K.TDTTTNTVEIGGSLGFSDK.G
7206	297 - 315	648.3135	1941.9186	1941.9168	0.95	0	66	2.6e-007	1	U	K.TDTTTNTVEIGGSLGFSDK.G
7207	297 - 315	971.9666	1941.9187	1941.9168	0.99	0	109	1.2e-011	1	U	K.TDTTTNTVEIGGSLGFSDK.G
7208	297 - 315	648.3135	1941.9188	1941.9168	1.04	0	43	4.6e-005	1	U	K.TDTTTNTVEIGGSLGFSDK.G
7209	297 - 315	648.3135	1941.9188	1941.9168	1.04	0	30	0.001	1	U	K.TDTTTNTVEIGGSLGFSDK.G
7210	297 - 315	648.3135	1941.9188	1941.9168	1.04	0	58	1.7e-006	1	U	K.TDTTTNTVEIGGSLGFSDK.G
7211	297 - 315	648.3135	1941.9188	1941.9168	1.04	0	43	4.8e-005	1	U	K.TDTTTNTVEIGGSLGFSDK.G
7212	297 - 315	648.3135	1941.9188	1941.9168	1.04	0	56	2.4e-006	1	U	K.TDTTTNTVEIGGSLGFSDK.G
7213	297 - 315	971.9667	1941.9189	1941.9168	1.11	0	73	5.4e-008	1	U	K.TDTTTNTVEIGGSLGFSDK.G
7214	297 - 315	971.9667	1941.9189	1941.9168	1.11	0	100	9.2e-011	1	U	K.TDTTTNTVEIGGSLGFSDK.G
7215	297 - 315	971.9667	1941.9189	1941.9168	1.11	0	89	1.4e-009	1	U	K.TDTTTNTVEIGGSLGFSDK.G
7216	297 - 315	648.3136	1941.9190	1941.9168	1.14	0	33	0.00048	1	U	K.TDTTTNTVEIGGSLGFSDK.G
7217	297 - 315	648.3136	1941.9190	1941.9168	1.14	0	50	9.1e-006	1	U	K.TDTTTNTVEIGGSLGFSDK.G
7218	297 - 315	971.9668	1941.9190	1941.9168	1.17	0	101	7.1e-011	1	U	K.TDTTTNTVEIGGSLGFSDK.G
7219	297 - 315	648.3137	1941.9192	1941.9168	1.23	0	38	0.00016	1	U	K.TDTTTNTVEIGGSLGFSDK.G
7220	297 - 315	648.3137	1941.9192	1941.9168	1.23	0	41	8.4e-005	1	U	K.TDTTTNTVEIGGSLGFSDK.G
7221	297 - 315	648.3137	1941.9192	1941.9168	1.23	0	43	5e-005	1	U	K.TDTTTNTVEIGGSLGFSDK.G
7222	297 - 315	648.3137	1941.9192	1941.9168	1.23	0	67	2.2e-007	1	U	K.TDTTTNTVEIGGSLGFSDK.G
7223	297 - 315	971.9669	1941.9192	1941.9168	1.24	0	109	1.2e-011	1	U	K.TDTTTNTVEIGGSLGFSDK.G
7224	297 - 315	971.9669	1941.9193	1941.9168	1.30	0	79	1.3e-008	1	U	K.TDTTTNTVEIGGSLGFSDK.G
7225	297 - 315	648.3137	1941.9193	1941.9168	1.32	0	43	5.2e-005	1	U	K.TDTTTNTVEIGGSLGFSDK.G
7226	297 - 315	971.9670	1941.9194	1941.9168	1.36	0	67	2.2e-007	1	U	K.TDTTTNTVEIGGSLGFSDK.G
7227	297 - 315	971.9671	1941.9197	1941.9168	1.49	0	103	5.4e-011	1	U	K.TDTTTNTVEIGGSLGFSDK.G
7228	297 - 315	648.3139	1941.9199	1941.9168	1.61	0	34	0.00044	1	U	K.TDTTTNTVEIGGSLGFSDK.G
7229	297 - 315	648.3140	1941.9201	1941.9168	1.70	0	42	5.8e-005	1	U	K.TDTTTNTVEIGGSLGFSDK.G
7230	297 - 315	648.3141	1941.9204	1941.9168	1.89	0	56	2.5e-006	1	U	K.TDTTTNTVEIGGSLGFSDK.G
7231	297 - 315	648.3141	1941.9206	1941.9168	1.98	0	47	2.2e-005	1	U	K.TDTTTNTVEIGGSLGFSDK.G
7232	297 - 315	648.3141	1941.9206	1941.9168	1.98	0	36	0.00023	1	U	K.TDTTTNTVEIGGSLGFSDK.G
7233	297 - 315	971.9677	1941.9208	1941.9168	2.05	0	80	9.4e-009	1	U	K.TDTTTNTVEIGGSLGFSDK.G
7234	297 - 315	648.3145	1941.9215	1941.9168	2.46	0	47	1.8e-005	1	U	K.TDTTTNTVEIGGSLGFSDK.G
7235	297 - 315	971.9681	1941.9216	1941.9168	2.49	0	85	3.1e-009	1	U	K.TDTTTNTVEIGGSLGFSDK.G
7236	297 - 315	971.9686	1941.9226	1941.9168	3.00	0	90	9.9e-010	1	U	K.TDTTTNTVEIGGSLGFSDK.G
7237	297 - 315	971.9691	1941.9237	1941.9168	3.56	0	65	3.4e-007	1	U	K.TDTTTNTVEIGGSLGFSDK.G
7238	297 - 315	971.9701	1941.9256	1941.9168	4.57	0	88	1.7e-009	1	U	K.TDTTTNTVEIGGSLGFSDK.G
1703	316 - 324	485.2541	968.4936	968.4967	-3.21	0	26	0.0025	1	U	K.GFSFSISPK.Y
1704	316 - 324	485.2548	968.4950	968.4967	-1.82	0	32	0.00058	1	U	K.GFSFSISPK.Y
1705	316 - 324	485.2549	968.4952	968.4967	-1.57	0	25	0.0035	1	U	K.GFSFSISPK.Y
1706	316 - 324	485.2549	968.4952	968.4967	-1.57	0	25	0.0034	1	U	K.GFSFSISPK.Y
1707	316 - 324	485.2551	968.4956	968.4967	-1.13	0	20	0.0099	1	U	K.GFSFSISPK.Y
1708	316 - 324	485.2552	968.4958	968.4967	-1.00	0	11	0.074	1	U	K.GFSFSISPK.Y

Query	Start - End	Observed	Mr(expt)	Mr(calc)	ppm	M	Score	Expect	Rank	U	Peptide
1709	316 - 324	485.2552	968.4959	968.4967	-0.88	0	18	0.017	1	U	K.GFSFSISPK.Y
1710	316 - 324	485.2553	968.4960	968.4967	-0.75	0	32	0.00058	1	U	K.GFSFSISPK.Y
1711	316 - 324	485.2553	968.4961	968.4967	-0.62	0	29	0.0012	1	U	K.GFSFSISPK.Y
1712	316 - 324	485.2553	968.4961	968.4967	-0.62	0	41	7.5e-005	1	U	K.GFSFSISPK.Y
1713	316 - 324	485.2554	968.4963	968.4967	-0.50	0	22	0.0062	1	U	K.GFSFSISPK.Y
1714	316 - 324	485.2554	968.4963	968.4967	-0.50	0	32	0.00061	1	U	K.GFSFSISPK.Y
1715	316 - 324	485.2554	968.4963	968.4967	-0.44	0	19	0.013	1	U	K.GFSFSISPK.Y
1716	316 - 324	485.2555	968.4964	968.4967	-0.37	0	29	0.0013	1	U	K.GFSFSISPK.Y
1717	316 - 324	485.2556	968.4966	968.4967	-0.18	0	27	0.0021	1	U	K.GFSFSISPK.Y
1718	316 - 324	485.2556	968.4966	968.4967	-0.12	0	26	0.0025	1	U	K.GFSFSISPK.Y
1719	316 - 324	485.2556	968.4966	968.4967	-0.12	0	40	9.4e-005	1	U	K.GFSFSISPK.Y
1720	316 - 324	485.2556	968.4967	968.4967	-0.059	0	29	0.0011	1	U	K.GFSFSISPK.Y
1721	316 - 324	485.2557	968.4968	968.4967	0.067	0	19	0.012	1	U	K.GFSFSISPK.Y
1722	316 - 324	485.2557	968.4968	968.4967	0.067	0	26	0.0025	1	U	K.GFSFSISPK.Y
1723	316 - 324	485.2557	968.4969	968.4967	0.13	0	29	0.0014	1	U	K.GFSFSISPK.Y
1724	316 - 324	485.2557	968.4969	968.4967	0.19	0	28	0.0016	1	U	K.GFSFSISPK.Y
1725	316 - 324	485.2557	968.4969	968.4967	0.19	0	26	0.0027	1	U	K.GFSFSISPK.Y
1726	316 - 324	485.2558	968.4970	968.4967	0.26	0	32	0.00058	1	U	K.GFSFSISPK.Y
1727	316 - 324	485.2558	968.4970	968.4967	0.26	0	24	0.0041	1	U	K.GFSFSISPK.Y
1728	316 - 324	485.2558	968.4970	968.4967	0.26	0	28	0.0015	1	U	K.GFSFSISPK.Y
1729	316 - 324	485.2558	968.4970	968.4967	0.26	0	26	0.0026	1	U	K.GFSFSISPK.Y
1730	316 - 324	485.2558	968.4970	968.4967	0.32	0	26	0.0025	1	U	K.GFSFSISPK.Y
1731	316 - 324	485.2558	968.4971	968.4967	0.38	0	7	0.18	1	U	K.GFSFSISPK.Y
1732	316 - 324	485.2559	968.4972	968.4967	0.51	0	15	0.029	1	U	K.GFSFSISPK.Y
1733	316 - 324	485.2559	968.4972	968.4967	0.51	0	31	0.00075	1	U	K.GFSFSISPK.Y
1734	316 - 324	485.2559	968.4973	968.4967	0.57	0	16	0.028	1	U	K.GFSFSISPK.Y
1735	316 - 324	485.2559	968.4973	968.4967	0.57	0	18	0.015	1	U	K.GFSFSISPK.Y
1736	316 - 324	485.2560	968.4974	968.4967	0.70	0	23	0.0052	1	U	K.GFSFSISPK.Y
1737	316 - 324	485.2560	968.4974	968.4967	0.70	0	40	9.4e-005	1	U	K.GFSFSISPK.Y
1738	316 - 324	485.2560	968.4975	968.4967	0.76	0	20	0.0098	1	U	K.GFSFSISPK.Y
1739	316 - 324	485.2560	968.4975	968.4967	0.76	0	23	0.0054	1	U	K.GFSFSISPK.Y
1740	316 - 324	485.2560	968.4975	968.4967	0.76	0	31	0.00087	1	U	K.GFSFSISPK.Y
1741	316 - 324	485.2561	968.4976	968.4967	0.89	0	30	0.00096	1	U	K.GFSFSISPK.Y
1742	316 - 324	485.2561	968.4977	968.4967	1.01	0	20	0.0096	1	U	K.GFSFSISPK.Y
1743	316 - 324	485.2562	968.4978	968.4967	1.08	0	29	0.0013	1	U	K.GFSFSISPK.Y
1744	316 - 324	485.2567	968.4988	968.4967	2.15	0	21	0.0082	1	U	K.GFSFSISPK.Y
1745	316 - 324	485.2568	968.4990	968.4967	2.34	0	27	0.0018	1	U	K.GFSFSISPK.Y
13950	325 - 354	1092.4907	3274.4503	3274.4651	-4.50	0	60	1e-006	1	U	K.YTHSWSSSTSVADTDSTTWSSQIGINTAER.A
13951	325 - 354	819.6210	3274.4550	3274.4651	-3.07	0	42	6.5e-005	1	U	K.YTHSWSSSTSVADTDSTTWSSQIGINTAER.A
13952	325 - 354	1092.4928	3274.4566	3274.4651	-2.60	0	45	2.8e-005	1	U	K.YTHSWSSSTSVADTDSTTWSSQIGINTAER.A
13953	325 - 354	819.6218	3274.4580	3274.4651	-2.17	0	12	0.065	1	U	K.YTHSWSSSTSVADTDSTTWSSQIGINTAER.A
13954	325 - 354	1092.4937	3274.4591	3274.4651	-1.82	0	54	3.7e-006	1	U	K.YTHSWSSSTSVADTDSTTWSSQIGINTAER.A
13955	325 - 354	1092.4939	3274.4599	3274.4651	-1.59	0	48	1.7e-005	1	U	K.YTHSWSSSTSVADTDSTTWSSQIGINTAER.A
13956	325 - 354	819.6224	3274.4606	3274.4651	-1.35	0	41	7.4e-005	1	U	K.YTHSWSSSTSVADTDSTTWSSQIGINTAER.A
13957	325 - 354	819.6226	3274.4611	3274.4651	-1.20	0	29	0.0012	1	U	K.YTHSWSSSTSVADTDSTTWSSQIGINTAER.A
13958	325 - 354	1092.4948	3274.4624	3274.4651	-0.81	0	27	0.0021	1	U	K.YTHSWSSSTSVADTDSTTWSSQIGINTAER.A
13959	325 - 354	1092.4951	3274.4635	3274.4651	-0.47	0	65	3.1e-007	1	U	K.YTHSWSSSTSVADTDSTTWSSQIGINTAER.A
13960	325 - 354	1092.4952	3274.4639	3274.4651	-0.36	0	40	9.4e-005	1	U	K.YTHSWSSSTSVADTDSTTWSSQIGINTAER.A
13961	325 - 354	1092.4952	3274.4639	3274.4651	-0.36	0	41	8.1e-005	1	U	K.YTHSWSSSTSVADTDSTTWSSQIGINTAER.A
13962	325 - 354	1092.4954	3274.4643	3274.4651	-0.25	0	30	0.0011	1	U	K.YTHSWSSSTSVADTDSTTWSSQIGINTAER.A
13963	325 - 354	1092.4955	3274.4646	3274.4651	-0.14	0	48	1.4e-005	1	U	K.YTHSWSSSTSVADTDSTTWSSQIGINTAER.A
13964	325 - 354	1092.4955	3274.4646	3274.4651	-0.14	0	37	0.00018	1	U	K.YTHSWSSSTSVADTDSTTWSSQIGINTAER.A
13965	325 - 354	819.6235	3274.4648	3274.4651	-0.086	0	33	0.00048	1	U	K.YTHSWSSSTSVADTDSTTWSSQIGINTAER.A
13966	325 - 354	819.6235	3274.4650	3274.4651	-0.011	0	28	0.0015	1	U	K.YTHSWSSSTSVADTDSTTWSSQIGINTAER.A
13967	325 - 354	1092.4957	3274.4654	3274.4651	0.086	0	58	1.7e-006	1	U	K.YTHSWSSSTSVADTDSTTWSSQIGINTAER.A
13968	325 - 354	1092.4957	3274.4654	3274.4651	0.086	0	66	2.6e-007	1	U	K.YTHSWSSSTSVADTDSTTWSSQIGINTAER.A
13969	325 - 354	1092.4957	3274.4654	3274.4651	0.086	0	35	0.00032	1	U	K.YTHSWSSSTSVADTDSTTWSSQIGINTAER.A
13970	325 - 354	1638.2400	3274.4654	3274.4651	0.11	0	49	1.2e-005	1	U	K.YTHSWSSSTSVADTDSTTWSSQIGINTAER.A
13971	325 - 354	1092.4959	3274.4657	3274.4651	0.20	0	27	0.002	1	U	K.YTHSWSSSTSVADTDSTTWSSQIGINTAER.A
13972	325 - 354	1092.4960	3274.4661	3274.4651	0.31	0	43	4.8e-005	1	U	K.YTHSWSSSTSVADTDSTTWSSQIGINTAER.A
13973	325 - 354	1092.4960	3274.4661	3274.4651	0.31	0	71	7.6e-008	1	U	K.YTHSWSSSTSVADTDSTTWSSQIGINTAER.A
13974	325 - 354	1092.4960	3274.4661	3274.4651	0.31	0	42	6.2e-005	1	U	K.YTHSWSSSTSVADTDSTTWSSQIGINTAER.A
13975	325 - 354	1092.4961	3274.4665	3274.4651	0.42	0	55	3.5e-006	1	U	K.YTHSWSSSTSVADTDSTTWSSQIGINTAER.A
13976	325 - 354	1092.4961	3274.4665	3274.4651	0.42	0	44	4.4e-005	1	U	K.YTHSWSSSTSVADTDSTTWSSQIGINTAER.A
13977	325 - 354	1092.4962	3274.4668	3274.4651	0.53	0	40	9e-005	1	U	K.YTHSWSSSTSVADTDSTTWSSQIGINTAER.A
13978	325 - 354	1092.4962	3274.4668	3274.4651	0.53	0	53	4.9e-006	1	U	K.YTHSWSSSTSVADTDSTTWSSQIGINTAER.A
13979	325 - 354	1092.4962	3274.4668	3274.4651	0.53	0	41	7.8e-005	1	U	K.YTHSWSSSTSVADTDSTTWSSQIGINTAER.A
13980	325 - 354	1092.4963	3274.4672	3274.4651	0.65	0	59	1.4e-006	1	U	K.YTHSWSSSTSVADTDSTTWSSQIGINTAER.A
13981	325 - 354	1092.4963	3274.4672	3274.4651	0.65	0	60	1.1e-006	1	U	K.YTHSWSSSTSVADTDSTTWSSQIGINTAER.A
13982	325 - 354	1092.4963	3274.4672	3274.4651	0.65	0	66	2.5e-007	1	U	K.YTHSWSSSTSVADTDSTTWSSQIGINTAER.A
13983	325 - 354	1092.4965	3274.4676	3274.4651	0.76	0	56	2.5e-006	1	U	K.YTHSWSSSTSVADTDSTTWSSQIGINTAER.A
13984	325 - 354	1092.4965	3274.4676	3274.4651	0.76	0	29	0.0012	1	U	K.YTHSWSSSTSVADTDSTTWSSQIGINTAER.A
13985	325 - 354	1092.4965	3274.4676	3274.4651	0.76	0	37	0.00018	1	U	K.YTHSWSSSTSVADTDSTTWSSQIGINTAER.A
13986	325 - 354	1092.4966	3274.4679	3274.4651	0.87	0	60	1e-006	1	U	K.YTHSWSSSTSVADTDSTTWSSQIGINTAER.A
13987	325 - 354	1092.4966	3274.4679	3274.4651	0.87	0	48	1.5e-005	1	U	K.YTHSWSSSTSVADTDSTTWSSQIGINTAER.A
13988	325 - 354	1092.4967	3274.4683	3274.4651	0.98	0	30	0.001	1	U	K.YTHSWSSSTSVADTDSTTWSSQIGINTAER.A
13989	325 - 354	1092.4967	3274.4683	3274.4651	0.98	0	65	3.3e-007	1	U	K.YTHSWSSSTSVADTDSTTWSSQIGINTAER.A
13990	325 - 354	1092.4968	3274.4686	3274.4651	1.09	0	38	0.00015	1	U	K.YTHSWSSSTSVADTDSTTWSSQIGINTAER.A
13991	325 - 354	1092.4969	3274.4690	3274.4651	1.20	0	46	2.7e-005	1	U	K.YTHSWSSSTSVADTDSTTWSSQIGINTAER.A
13992	325 - 354	1092.4969	3274.4690	3274.4651	1.20	0	35	0.00032	1	U	K.YTHSWSSSTSVADTDSTTWSSQIGINTAER.A
13993	325 - 354	1092.4969	3274.4690	3274.4651	1.20	0	39	0.00013	1	U	K.YTHSWSSSTSVADTDSTTWSSQIGINTAER.A
13994	325 - 354	1092.4969	3274.4690	3274.4651	1.20	0	44	3.9e-005	1	U	K.YTHSWSSSTSVADTDSTTWSSQIGINTAER.A
13995	325 - 354	1092.4969	3274.4690	3274.4651	1.20	0	55	3.4e-006	1	U	K.YTHSWSSSTSVADTDSTTWSSQIGINTAER.A
13996	325 - 354	819.6246	3274.4694	3274.4651	1.33	0	18	0.018	1	U	K.YTHSWSSSTSVADTDSTTWSSQIGINTAER.A
13997	325 - 354	1092.4972	3274.4697	3274.4651	1.43	0	49	1.2e-005	1	U	K.YTHSWSSSTSVADTDSTTWSSQIGINTAER.A
13998	325 - 354	1092.4974	3274.4705	3274.4651	1.65	0	43	5.1e-005	1	U	K.YTHSWSSSTSVADTDSTTWSSQIGINTAER.A
13999	325 - 354	1092.4977	3274.4712	3274.4651	1.88	0	41	8.5e-005	1	U	K.YTHSWSSSTSVADTDSTTWSSQIGINTAER.A
14000	325 - 354	1092.4978	3274.4716	3274.4651	1.99	0	55	3.3e-006	1	U	K.YTHSWSSSTSVADTDSTTWSSQIGINTAER.A

Query	Start - End	Observed	Mr(expt)	Mr(calc)	ppm	M	Score	Expect	Rank	U	Peptide
14001	325 - 354	1092.4987	3274.4741	3274.4651	2.77	0	56	2.3e-006	1	U	K.YTHSWSSSTSVADTDSTTWSSQIGINTAER.A
14002	325 - 354	1092.4990	3274.4752	3274.4651	3.11	0	50	9.8e-006	1	U	K.YTHSWSSSTSVADTDSTTWSSQIGINTAER.A
14003	325 - 354	1092.4991	3274.4756	3274.4651	3.22	0	54	3.9e-006	1	U	K.YTHSWSSSTSVADTDSTTWSSQIGINTAER.A
14004	325 - 354	1092.4993	3274.4760	3274.4651	3.33	0	53	4.7e-006	1	U	K.YTHSWSSSTSVADTDSTTWSSQIGINTAER.A
1376	355 - 362	460.7500	919.4854	919.4875	-2.34	0	26	0.0026	1	U	R.AYLNANVR.Y
1377	355 - 362	460.7505	919.4865	919.4875	-1.07	0	16	0.023	1	U	R.AYLNANVR.Y
1378	355 - 362	460.7505	919.4865	919.4875	-1.07	0	35	0.00029	1	U	R.AYLNANVR.Y
1379	355 - 362	460.7506	919.4867	919.4875	-0.94	0	22	0.0069	1	U	R.AYLNANVR.Y
1380	355 - 362	460.7508	919.4870	919.4875	-0.61	0	36	0.00026	1	U	R.AYLNANVR.Y
1381	355 - 362	460.7508	919.4870	919.4875	-0.54	0	11	0.082	1	U	R.AYLNANVR.Y
1382	355 - 362	460.7508	919.4871	919.4875	-0.48	0	15	0.035	1	U	R.AYLNANVR.Y
1383	355 - 362	460.7509	919.4872	919.4875	-0.34	0	7	0.19	1	U	R.AYLNANVR.Y
1384	355 - 362	460.7509	919.4872	919.4875	-0.34	0	9	0.13	1	U	R.AYLNANVR.Y
1385	355 - 362	460.7509	919.4873	919.4875	-0.21	0	16	0.027	1	U	R.AYLNANVR.Y
1386	355 - 362	460.7510	919.4874	919.4875	-0.14	0	12	0.063	1	U	R.AYLNANVR.Y
1387	355 - 362	460.7510	919.4875	919.4875	-0.078	0	12	0.057	1	U	R.AYLNANVR.Y
1388	355 - 362	460.7510	919.4875	919.4875	-0.078	0	17	0.019	1	U	R.AYLNANVR.Y
1389	355 - 362	460.7511	919.4876	919.4875	0.054	0	17	0.018	1	U	R.AYLNANVR.Y
1390	355 - 362	307.5032	919.4879	919.4875	0.37	0	33	0.00054	1	U	R.AYLNANVR.Y
1391	355 - 362	460.7515	919.4884	919.4875	0.98	0	16	0.027	1	U	R.AYLNANVR.Y
16318	363 - 412	1054.3153	5266.5402	5266.5371	0.58	0	28	0.0014	1	U	R.YYNGGTAPIYDLKPTTNFVFQNSGDSITTITAGPNQIGNSLGAGI
16319	363 - 412	1317.6434	5266.5446	5266.5371	1.42	0	36	0.00023	1	U	R.YYNGGTAPIYDLKPTTNFVFQNSGDSITTITAGPNQIGNSLGAGI
1447	413 - 421	464.7572	927.4999	927.5025	-2.89	0	18	0.015	1	U	K.GQAPISLDK.A
1448	413 - 421	464.7583	927.5020	927.5025	-0.52	0	22	0.0059	1	U	K.GQAPISLDK.A
1449	413 - 421	464.7583	927.5021	927.5025	-0.46	0	11	0.086	1	U	K.GQAPISLDK.A
1450	413 - 421	464.7584	927.5022	927.5025	-0.39	0	17	0.018	1	U	K.GQAPISLDK.A
1451	413 - 421	464.7584	927.5023	927.5025	-0.26	0	33	0.00049	1	U	K.GQAPISLDK.A
1452	413 - 421	464.7585	927.5024	927.5025	-0.13	0	11	0.089	1	U	K.GQAPISLDK.A
1453	413 - 421	464.7586	927.5027	927.5025	0.14	0	8	0.14	1	U	K.GQAPISLDK.A
1454	413 - 421	464.7587	927.5028	927.5025	0.34	0	7	0.18	1	U	K.GQAPISLDK.A
1455	413 - 421	464.7592	927.5038	927.5025	1.32	0	12	0.069	1	U	K.GQAPISLDK.A
5751	413 - 429	849.9540	1697.8934	1697.8948	-0.82	1	47	2.2e-005	1	U	K.GQAPISLDKANEAGTVK.I
5752	413 - 429	566.9719	1697.8938	1697.8948	-0.61	1	27	0.002	1	U	K.GQAPISLDKANEAGTVK.I
5753	413 - 429	566.9719	1697.8938	1697.8948	-0.61	1	36	0.00024	1	U	K.GQAPISLDKANEAGTVK.I
5754	413 - 429	849.9543	1697.8941	1697.8948	-0.38	1	77	1.9e-008	1	U	K.GQAPISLDKANEAGTVK.I
720	422 - 429	395.2086	788.4026	788.4028	-0.24	0	46	2.5e-005	1	U	K.ANEAGTVK.I
721	422 - 429	395.2086	788.4026	788.4028	-0.24	0	46	2.4e-005	1	U	K.ANEAGTVK.I
722	422 - 429	395.2086	788.4026	788.4028	-0.24	0	55	3e-006	1	U	K.ANEAGTVK.I
723	422 - 429	395.2087	788.4028	788.4028	-0.014	0	46	2.5e-005	1	U	K.ANEAGTVK.I
2574	430 - 439	557.8083	1113.6020	1113.6030	-0.83	0	12	0.057	1	U	K.IAINAEQLDK.I
2575	430 - 439	557.8083	1113.6020	1113.6030	-0.83	0	27	0.0021	1	U	K.IAINAEQLDK.I
2576	430 - 439	557.8084	1113.6021	1113.6030	-0.72	0	29	0.0013	1	U	K.IAINAEQLDK.I
2577	430 - 439	557.8085	1113.6024	1113.6030	-0.50	0	27	0.0018	1	U	K.IAINAEQLDK.I
2578	430 - 439	557.8086	1113.6026	1113.6030	-0.28	0	19	0.012	1	U	K.IAINAEQLDK.I
2579	430 - 439	557.8087	1113.6028	1113.6030	-0.17	0	29	0.0011	1	U	K.IAINAEQLDK.I
2580	430 - 439	557.8087	1113.6028	1113.6030	-0.17	0	29	0.0012	1	U	K.IAINAEQLDK.I
2581	430 - 439	557.8087	1113.6028	1113.6030	-0.17	0	33	0.0005	1	U	K.IAINAEQLDK.I
2582	430 - 439	557.8087	1113.6028	1113.6030	-0.17	0	67	2.1e-007	1	U	K.IAINAEQLDK.I
2583	430 - 439	557.8087	1113.6029	1113.6030	-0.064	0	37	0.0002	1	U	K.IAINAEQLDK.I
2584	430 - 439	557.8087	1113.6029	1113.6030	-0.064	0	48	1.5e-005	1	U	K.IAINAEQLDK.I
2585	430 - 439	557.8088	1113.6030	1113.6030	0.046	0	31	0.00088	1	U	K.IAINAEQLDK.I
2586	430 - 439	557.8088	1113.6030	1113.6030	0.046	0	61	8.6e-007	1	U	K.IAINAEQLDK.I
2587	430 - 439	557.8089	1113.6032	1113.6030	0.26	0	22	0.0059	1	U	K.IAINAEQLDK.I
2589	430 - 439	557.8089	1113.6032	1113.6030	0.26	0	28	0.0016	1	U	K.IAINAEQLDK.I
2590	430 - 439	557.8089	1113.6032	1113.6030	0.26	0	58	1.4e-006	1	U	K.IAINAEQLDK.I
2591	430 - 439	557.8090	1113.6034	1113.6030	0.37	0	34	0.00038	1	U	K.IAINAEQLDK.I
2592	430 - 439	557.8090	1113.6034	1113.6030	0.37	0	23	0.0048	1	U	K.IAINAEQLDK.I
2593	430 - 439	557.8090	1113.6034	1113.6030	0.37	0	31	0.00076	1	U	K.IAINAEQLDK.I
2594	430 - 439	557.8090	1113.6035	1113.6030	0.48	0	23	0.0048	1	U	K.IAINAEQLDK.I
2595	430 - 439	557.8091	1113.6036	1113.6030	0.59	0	19	0.014	1	U	K.IAINAEQLDK.I
2596	430 - 439	557.8091	1113.6037	1113.6030	0.70	0	7	0.18	1	U	K.IAINAEQLDK.I
2597	430 - 439	557.8091	1113.6037	1113.6030	0.70	0	26	0.0024	1	U	K.IAINAEQLDK.I
2598	430 - 439	557.8092	1113.6039	1113.6030	0.81	0	28	0.0015	1	U	K.IAINAEQLDK.I
2599	430 - 439	557.8093	1113.6040	1113.6030	0.92	0	17	0.022	1	U	K.IAINAEQLDK.I
2600	430 - 439	557.8093	1113.6040	1113.6030	0.92	0	15	0.035	1	U	K.IAINAEQLDK.I
2601	430 - 439	557.8093	1113.6040	1113.6030	0.92	0	36	0.00025	1	U	K.IAINAEQLDK.I
2602	430 - 439	557.8094	1113.6042	1113.6030	1.14	0	23	0.0053	1	U	K.IAINAEQLDK.I
2603	430 - 439	557.8095	1113.6045	1113.6030	1.36	0	19	0.014	1	U	K.IAINAEQLDK.I
2604	430 - 439	557.8095	1113.6045	1113.6030	1.36	0	20	0.01	1	U	K.IAINAEQLDK.I
2605	430 - 439	557.8096	1113.6047	1113.6030	1.58	0	10	0.11	1	U	K.IAINAEQLDK.I
12439	430 - 455	966.1804	2895.5192	2895.5301	-3.75	1	33	0.00049	1	U	K.IAINAEQLDKIQAGTEILNIETTQNR.G
12440	430 - 455	724.8883	2895.5241	2895.5301	-2.07	1	39	0.00014	1	U	K.IAINAEQLDKIQAGTEILNIETTQNR.G
12441	430 - 455	724.8885	2895.5249	2895.5301	-1.81	1	18	0.015	1	U	K.IAINAEQLDKIQAGTEILNIETTQNR.G
12442	430 - 455	966.1825	2895.5257	2895.5301	-1.54	1	53	4.5e-006	1	U	K.IAINAEQLDKIQAGTEILNIETTQNR.G
12443	430 - 455	724.8894	2895.5285	2895.5301	-0.55	1	49	1.3e-005	1	U	K.IAINAEQLDKIQAGTEILNIETTQNR.G
12444	430 - 455	966.1835	2895.5286	2895.5301	-0.52	1	38	0.00016	1	U	K.IAINAEQLDKIQAGTEILNIETTQNR.G
12445	430 - 455	966.1835	2895.5286	2895.5301	-0.52	1	45	3.2e-005	1	U	K.IAINAEQLDKIQAGTEILNIETTQNR.G
12446	430 - 455	966.1835	2895.5286	2895.5301	-0.52	1	45	3e-005	1	U	K.IAINAEQLDKIQAGTEILNIETTQNR.G
12447	430 - 455	724.8895	2895.5288	2895.5301	-0.47	1	41	8e-005	1	U	K.IAINAEQLDKIQAGTEILNIETTQNR.G
12448	430 - 455	724.8895	2895.5290	2895.5301	-0.38	1	4	0.37	1	U	K.IAINAEQLDKIQAGTEILNIETTQNR.G
12449	430 - 455	966.1837	2895.5291	2895.5301	-0.33	1	44	3.7e-005	1	U	K.IAINAEQLDKIQAGTEILNIETTQNR.G
12450	430 - 455	724.8896	2895.5295	2895.5301	-0.21	1	49	1.3e-005	1	U	K.IAINAEQLDKIQAGTEILNIETTQNR.G
12451	430 - 455	966.1840	2895.5301	2895.5301	-0.019	1	37	0.0002	1	U	K.IAINAEQLDKIQAGTEILNIETTQNR.G
12452	430 - 455	724.8898	2895.5302	2895.5301	0.041	1	48	1.7e-005	1	U	K.IAINAEQLDKIQAGTEILNIETTQNR.G
12453	430 - 455	966.1841	2895.5304	2895.5301	0.11	1	44	4e-005	1	U	K.IAINAEQLDKIQAGTEILNIETTQNR.G
12454	430 - 455	966.1841	2895.5304	2895.5301	0.11	1	50	9.5e-006	1	U	K.IAINAEQLDKIQAGTEILNIETTQNR.G
12455	430 - 455	724.8899	2895.5305	2895.5301	0.13	1	70	8.9e-008	1	U	K.IAINAEQLDKIQAGTEILNIETTQNR.G
12456	430 - 455	966.1841	2895.5306	2895.5301	0.17	1	49	1.3e-005	1	U	K.IAINAEQLDKIQAGTEILNIETTQNR.G

Query	Start - End	Observed	Mr(expt)	Mr(calc)	ppm	M	Score	Expect	Rank	U	Peptide
12457	430 - 455	724.8900	2895.5307	2895.5301	0.21	1	56	2.3e-006	1	U	K.IAINAEQLDKIQAGTEILNIETTQNR.G
12458	430 - 455	966.1842	2895.5308	2895.5301	0.23	1	56	2.6e-006	1	U	K.IAINAEQLDKIQAGTEILNIETTQNR.G
12459	430 - 455	966.1843	2895.5310	2895.5301	0.30	1	57	2e-006	1	U	K.IAINAEQLDKIQAGTEILNIETTQNR.G
12460	430 - 455	966.1843	2895.5312	2895.5301	0.36	1	45	2.9e-005	1	U	K.IAINAEQLDKIQAGTEILNIETTQNR.G
12461	430 - 455	966.1843	2895.5312	2895.5301	0.36	1	28	0.0014	1	U	K.IAINAEQLDKIQAGTEILNIETTQNR.G
12462	430 - 455	966.1844	2895.5313	2895.5301	0.42	1	22	0.0068	1	U	K.IAINAEQLDKIQAGTEILNIETTQNR.G
12463	430 - 455	966.1844	2895.5315	2895.5301	0.49	1	43	5.3e-005	1	U	K.IAINAEQLDKIQAGTEILNIETTQNR.G
12464	430 - 455	966.1844	2895.5315	2895.5301	0.49	1	43	5.2e-005	1	U	K.IAINAEQLDKIQAGTEILNIETTQNR.G
12465	430 - 455	966.1846	2895.5319	2895.5301	0.61	1	42	6.7e-005	1	U	K.IAINAEQLDKIQAGTEILNIETTQNR.G
12466	430 - 455	966.1846	2895.5321	2895.5301	0.68	1	46	2.5e-005	1	U	K.IAINAEQLDKIQAGTEILNIETTQNR.G
12467	430 - 455	966.1846	2895.5321	2895.5301	0.68	1	41	7.8e-005	1	U	K.IAINAEQLDKIQAGTEILNIETTQNR.G
12468	430 - 455	724.8903	2895.5322	2895.5301	0.71	1	32	0.00056	1	U	K.IAINAEQLDKIQAGTEILNIETTQNR.G
12469	430 - 455	724.8903	2895.5322	2895.5301	0.71	1	50	1.1e-005	1	U	K.IAINAEQLDKIQAGTEILNIETTQNR.G
12470	430 - 455	966.1847	2895.5322	2895.5301	0.74	1	38	0.00016	1	U	K.IAINAEQLDKIQAGTEILNIETTQNR.G
12471	430 - 455	966.1847	2895.5322	2895.5301	0.74	1	56	2.7e-006	1	U	K.IAINAEQLDKIQAGTEILNIETTQNR.G
12472	430 - 455	966.1848	2895.5324	2895.5301	0.80	1	57	2e-006	1	U	K.IAINAEQLDKIQAGTEILNIETTQNR.G
12473	430 - 455	966.1848	2895.5326	2895.5301	0.87	1	43	4.7e-005	1	U	K.IAINAEQLDKIQAGTEILNIETTQNR.G
12474	430 - 455	724.8904	2895.5327	2895.5301	0.88	1	42	6.2e-005	1	U	K.IAINAEQLDKIQAGTEILNIETTQNR.G
12475	430 - 455	966.1849	2895.5328	2895.5301	0.93	1	49	1.3e-005	1	U	K.IAINAEQLDKIQAGTEILNIETTQNR.G
12476	430 - 455	724.8905	2895.5329	2895.5301	0.97	1	14	0.044	1	U	K.IAINAEQLDKIQAGTEILNIETTQNR.G
12477	430 - 455	724.8905	2895.5329	2895.5301	0.97	1	25	0.0034	1	U	K.IAINAEQLDKIQAGTEILNIETTQNR.G
12478	430 - 455	966.1849	2895.5330	2895.5301	0.99	1	57	1.9e-006	1	U	K.IAINAEQLDKIQAGTEILNIETTQNR.G
12479	430 - 455	966.1849	2895.5330	2895.5301	0.99	1	65	3.4e-007	1	U	K.IAINAEQLDKIQAGTEILNIETTQNR.G
12480	430 - 455	724.8906	2895.5332	2895.5301	1.05	1	39	0.00013	1	U	K.IAINAEQLDKIQAGTEILNIETTQNR.G
12481	430 - 455	966.1850	2895.5332	2895.5301	1.06	1	42	6.5e-005	1	U	K.IAINAEQLDKIQAGTEILNIETTQNR.G
12482	430 - 455	966.1850	2895.5332	2895.5301	1.06	1	44	4.3e-005	1	U	K.IAINAEQLDKIQAGTEILNIETTQNR.G
12483	430 - 455	966.1851	2895.5333	2895.5301	1.12	1	47	2e-005	1	U	K.IAINAEQLDKIQAGTEILNIETTQNR.G
12484	430 - 455	966.1851	2895.5333	2895.5301	1.12	1	37	0.00019	1	U	K.IAINAEQLDKIQAGTEILNIETTQNR.G
12485	430 - 455	966.1851	2895.5335	2895.5301	1.18	1	35	0.00034	1	U	K.IAINAEQLDKIQAGTEILNIETTQNR.G
12486	430 - 455	724.8907	2895.5336	2895.5301	1.22	1	47	2.1e-005	1	U	K.IAINAEQLDKIQAGTEILNIETTQNR.G
12487	430 - 455	966.1852	2895.5339	2895.5301	1.31	1	35	0.00034	1	U	K.IAINAEQLDKIQAGTEILNIETTQNR.G
12488	430 - 455	966.1853	2895.5341	2895.5301	1.37	1	32	0.00063	1	U	K.IAINAEQLDKIQAGTEILNIETTQNR.G
12489	430 - 455	724.8908	2895.5341	2895.5301	1.39	1	18	0.017	1	U	K.IAINAEQLDKIQAGTEILNIETTQNR.G
12490	430 - 455	724.8909	2895.5344	2895.5301	1.47	1	24	0.0043	1	U	K.IAINAEQLDKIQAGTEILNIETTQNR.G
12491	430 - 455	724.8909	2895.5344	2895.5301	1.47	1	42	6.9e-005	1	U	K.IAINAEQLDKIQAGTEILNIETTQNR.G
12492	430 - 455	724.8909	2895.5344	2895.5301	1.47	1	22	0.0063	1	U	K.IAINAEQLDKIQAGTEILNIETTQNR.G
12493	430 - 455	966.1854	2895.5344	2895.5301	1.50	1	49	1.3e-005	1	U	K.IAINAEQLDKIQAGTEILNIETTQNR.G
12494	430 - 455	724.8909	2895.5346	2895.5301	1.56	1	18	0.015	1	U	K.IAINAEQLDKIQAGTEILNIETTQNR.G
12495	430 - 455	724.8909	2895.5346	2895.5301	1.56	1	57	2.1e-006	1	U	K.IAINAEQLDKIQAGTEILNIETTQNR.G
12496	430 - 455	966.1855	2895.5346	2895.5301	1.56	1	37	0.00018	1	U	K.IAINAEQLDKIQAGTEILNIETTQNR.G
12497	430 - 455	966.1855	2895.5348	2895.5301	1.63	1	32	0.00062	1	U	K.IAINAEQLDKIQAGTEILNIETTQNR.G
12498	430 - 455	966.1856	2895.5350	2895.5301	1.69	1	49	1.3e-005	1	U	K.IAINAEQLDKIQAGTEILNIETTQNR.G
12499	430 - 455	1448.7751	2895.5357	2895.5301	1.95	1	75	3.1e-008	1	U	K.IAINAEQLDKIQAGTEILNIETTQNR.G
12500	430 - 455	724.8912	2895.5358	2895.5301	1.98	1	34	0.00041	1	U	K.IAINAEQLDKIQAGTEILNIETTQNR.G
12501	430 - 455	724.8914	2895.5363	2895.5301	2.15	1	34	0.00039	1	U	K.IAINAEQLDKIQAGTEILNIETTQNR.G
12502	430 - 455	966.1863	2895.5372	2895.5301	2.45	1	34	0.00042	1	U	K.IAINAEQLDKIQAGTEILNIETTQNR.G
12503	430 - 455	724.8918	2895.5380	2895.5301	2.74	1	15	0.031	1	U	K.IAINAEQLDKIQAGTEILNIETTQNR.G
12504	430 - 455	724.8922	2895.5395	2895.5301	3.24	1	20	0.0096	1	U	K.IAINAEQLDKIQAGTEILNIETTQNR.G
12505	430 - 455	966.1876	2895.5409	2895.5301	3.71	1	44	3.7e-005	1	U	K.IAINAEQLDKIQAGTEILNIETTQNR.G
12506	430 - 455	724.8926	2895.5412	2895.5301	3.83	1	25	0.0029	1	U	K.IAINAEQLDKIQAGTEILNIETTQNR.G
12507	430 - 455	966.1882	2895.5427	2895.5301	4.34	1	27	0.0021	1	U	K.IAINAEQLDKIQAGTEILNIETTQNR.G
6371	440 - 455	900.9726	1799.9306	1799.9377	-3.93	0	95	3.5e-010	1	U	K.IQAGTEILNIETTQNR.G
6372	440 - 455	900.9741	1799.9336	1799.9377	-2.30	0	79	1.2e-008	1	U	K.IQAGTEILNIETTQNR.G
6373	440 - 455	900.9745	1799.9345	1799.9377	-1.76	0	38	0.00017	1	U	K.IQAGTEILNIETTQNR.G
6374	440 - 455	900.9750	1799.9354	1799.9377	-1.29	0	26	0.0027	1	U	K.IQAGTEILNIETTQNR.G
6375	440 - 455	600.9858	1799.9355	1799.9377	-1.23	0	45	3.1e-005	1	U	K.IQAGTEILNIETTQNR.G
6376	440 - 455	900.9750	1799.9355	1799.9377	-1.22	0	19	0.013	1	U	K.IQAGTEILNIETTQNR.G
6377	440 - 455	900.9753	1799.9360	1799.9377	-0.95	0	49	1.2e-005	1	U	K.IQAGTEILNIETTQNR.G
6378	440 - 455	900.9753	1799.9360	1799.9377	-0.95	0	119	1.2e-012	1	U	K.IQAGTEILNIETTQNR.G
6379	440 - 455	900.9753	1799.9361	1799.9377	-0.88	0	78	1.7e-008	1	U	K.IQAGTEILNIETTQNR.G
6380	440 - 455	900.9755	1799.9365	1799.9377	-0.68	0	26	0.0026	1	U	K.IQAGTEILNIETTQNR.G
6381	440 - 455	900.9755	1799.9365	1799.9377	-0.68	0	75	3.4e-008	1	U	K.IQAGTEILNIETTQNR.G
6382	440 - 455	900.9757	1799.9369	1799.9377	-0.47	0	30	0.00099	1	U	K.IQAGTEILNIETTQNR.G
6383	440 - 455	600.9863	1799.9370	1799.9377	-0.41	0	52	5.9e-006	1	U	K.IQAGTEILNIETTQNR.G
6384	440 - 455	900.9758	1799.9370	1799.9377	-0.40	0	97	2.2e-010	1	U	K.IQAGTEILNIETTQNR.G
6385	440 - 455	900.9759	1799.9372	1799.9377	-0.27	0	110	9.2e-012	1	U	K.IQAGTEILNIETTQNR.G
6386	440 - 455	600.9864	1799.9373	1799.9377	-0.21	0	32	0.00057	1	U	K.IQAGTEILNIETTQNR.G
6387	440 - 455	900.9760	1799.9374	1799.9377	-0.20	0	46	2.8e-005	1	U	K.IQAGTEILNIETTQNR.G
6388	440 - 455	900.9760	1799.9374	1799.9377	-0.20	0	24	0.0038	1	U	K.IQAGTEILNIETTQNR.G
6389	440 - 455	900.9760	1799.9375	1799.9377	-0.13	0	104	4e-011	1	U	K.IQAGTEILNIETTQNR.G
6390	440 - 455	600.9864	1799.9375	1799.9377	-0.11	0	45	3e-005	1	U	K.IQAGTEILNIETTQNR.G
6391	440 - 455	900.9761	1799.9376	1799.9377	-0.066	0	74	3.8e-008	1	U	K.IQAGTEILNIETTQNR.G
6392	440 - 455	900.9762	1799.9378	1799.9377	0.070	0	65	2.9e-007	1	U	K.IQAGTEILNIETTQNR.G
6393	440 - 455	900.9763	1799.9380	1799.9377	0.14	0	23	0.0045	1	U	K.IQAGTEILNIETTQNR.G
6394	440 - 455	900.9764	1799.9382	1799.9377	0.27	0	85	3e-009	1	U	K.IQAGTEILNIETTQNR.G
6395	440 - 455	900.9764	1799.9382	1799.9377	0.27	0	118	1.6e-012	1	U	K.IQAGTEILNIETTQNR.G
6396	440 - 455	600.9867	1799.9383	1799.9377	0.30	0	31	0.00073	1	U	K.IQAGTEILNIETTQNR.G
6397	440 - 455	900.9764	1799.9383	1799.9377	0.34	0	68	1.7e-007	1	U	K.IQAGTEILNIETTQNR.G
6398	440 - 455	900.9765	1799.9384	1799.9377	0.41	0	36	0.00028	1	U	K.IQAGTEILNIETTQNR.G
6399	440 - 455	900.9766	1799.9386	1799.9377	0.48	0	28	0.0016	1	U	K.IQAGTEILNIETTQNR.G
6400	440 - 455	600.9869	1799.9388	1799.9377	0.61	0	32	0.00068	1	U	K.IQAGTEILNIETTQNR.G
6401	440 - 455	900.9767	1799.9388	1799.9377	0.61	0	38	0.00017	1	U	K.IQAGTEILNIETTQNR.G
6402	440 - 455	900.9767	1799.9388	1799.9377	0.61	0	49	1.3e-005	1	U	K.IQAGTEILNIETTQNR.G
6403	440 - 455	900.9767	1799.9388	1799.9377	0.61	0	59	1.2e-006	1	U	K.IQAGTEILNIETTQNR.G
6404	440 - 455	900.9767	1799.9389	1799.9377	0.68	0	49	1.1e-005	1	U	K.IQAGTEILNIETTQNR.G
6405	440 - 455	600.9870	1799.9392	1799.9377	0.81	0	46	2.6e-005	1	U	K.IQAGTEILNIETTQNR.G
6406	440 - 455	900.9769	1799.9392	1799.9377	0.82	0	36	0.00023	1	U	K.IQAGTEILNIETTQNR.G
6407	440 - 455	900.9769	1799.9393	1799.9377	0.88	0	49	1.4e-005	1	U	K.IQAGTEILNIETTQNR.G

Query	Start - End	Observed	Mr(expt)	Mr(calc)	ppm	M	Score	Expect	Rank	U	Peptide
6408	440 - 455	900.9769	1799.9393	1799.9377	0.88	0	97	2e-010	1	U	K.IQAGTEILNIETTQNR.G
6409	440 - 455	900.9770	1799.9394	1799.9377	0.95	0	41	8.8e-005	1	U	K.IQAGTEILNIETTQNR.G
6410	440 - 455	600.9871	1799.9395	1799.9377	1.01	0	13	0.05	1	U	K.IQAGTEILNIETTQNR.G
6411	440 - 455	600.9871	1799.9395	1799.9377	1.01	0	31	0.00071	1	U	K.IQAGTEILNIETTQNR.G
6412	440 - 455	900.9771	1799.9395	1799.9377	1.02	0	28	0.0016	1	U	K.IQAGTEILNIETTQNR.G
6413	440 - 455	900.9771	1799.9397	1799.9377	1.09	0	119	1.2e-012	1	U	K.IQAGTEILNIETTQNR.G
6414	440 - 455	900.9772	1799.9399	1799.9377	1.22	0	75	2.9e-008	1	U	K.IQAGTEILNIETTQNR.G
6415	440 - 455	900.9772	1799.9399	1799.9377	1.22	0	81	7.5e-009	1	U	K.IQAGTEILNIETTQNR.G
6416	440 - 455	900.9773	1799.9400	1799.9377	1.29	0	37	0.00018	1	U	K.IQAGTEILNIETTQNR.G
6417	440 - 455	900.9774	1799.9402	1799.9377	1.36	0	47	2e-005	1	U	K.IQAGTEILNIETTQNR.G
6418	440 - 455	900.9774	1799.9403	1799.9377	1.43	0	22	0.0067	1	U	K.IQAGTEILNIETTQNR.G
6419	440 - 455	900.9775	1799.9404	1799.9377	1.49	0	49	1.2e-005	1	U	K.IQAGTEILNIETTQNR.G
6420	440 - 455	900.9775	1799.9405	1799.9377	1.56	0	56	2.6e-006	1	U	K.IQAGTEILNIETTQNR.G
6421	440 - 455	900.9777	1799.9408	1799.9377	1.70	0	76	2.2e-008	1	U	K.IQAGTEILNIETTQNR.G
6422	440 - 455	900.9780	1799.9415	1799.9377	2.10	0	44	3.6e-005	1	U	K.IQAGTEILNIETTQNR.G
6423	440 - 455	900.9781	1799.9416	1799.9377	2.17	0	57	1.8e-006	1	U	K.IQAGTEILNIETTQNR.G
6424	440 - 455	900.9782	1799.9419	1799.9377	2.31	0	37	0.00018	1	U	K.IQAGTEILNIETTQNR.G
6425	440 - 455	900.9786	1799.9426	1799.9377	2.72	0	81	7.5e-009	1	U	K.IQAGTEILNIETTQNR.G
6426	440 - 455	900.9786	1799.9427	1799.9377	2.78	0	93	4.8e-010	1	U	K.IQAGTEILNIETTQNR.G
6427	440 - 455	900.9805	1799.9465	1799.9377	4.89	0	61	8.9e-007	1	U	K.IQAGTEILNIETTQNR.G
12004	440 - 464	935.4858	2803.4357	2803.4352	0.19	1	15	0.033	1	U	K.IQAGTEILNIETTQNRGQYGILDEK.G
2076	456 - 464	511.7595	1021.5044	1021.5080	-3.51	0	24	0.0036	1	U	R.GQYGILDEK.G
2077	456 - 464	511.7604	1021.5063	1021.5080	-1.65	0	12	0.066	1	U	R.GQYGILDEK.G
2078	456 - 464	511.7605	1021.5064	1021.5080	-1.53	0	27	0.0018	1	U	R.GQYGILDEK.G
2079	456 - 464	511.7607	1021.5069	1021.5080	-1.06	0	49	1.3e-005	1	U	R.GQYGILDEK.G
2080	456 - 464	511.7608	1021.5071	1021.5080	-0.94	0	29	0.0013	1	U	R.GQYGILDEK.G
2081	456 - 464	511.7610	1021.5074	1021.5080	-0.58	0	48	1.7e-005	1	U	R.GQYGILDEK.G
2082	456 - 464	511.7610	1021.5075	1021.5080	-0.52	0	45	3.1e-005	1	U	R.GQYGILDEK.G
2083	456 - 464	511.7613	1021.5080	1021.5080	0.021	0	25	0.003	1	U	R.GQYGILDEK.G
2084	456 - 464	511.7613	1021.5080	1021.5080	0.021	0	41	8.3e-005	1	U	R.GQYGILDEK.G
2085	456 - 464	511.7614	1021.5082	1021.5080	0.14	0	9	0.11	1	U	R.GQYGILDEK.G
2086	456 - 464	511.7614	1021.5083	1021.5080	0.32	0	40	9.4e-005	1	U	R.GQYGILDEK.G
2087	456 - 464	511.7615	1021.5085	1021.5080	0.44	0	16	0.027	1	U	R.GQYGILDEK.G
2088	456 - 464	511.7619	1021.5093	1021.5080	1.21	0	21	0.0081	1	U	R.GQYGILDEK.G
2089	456 - 464	511.7623	1021.5100	1021.5080	1.99	0	7	0.18	1	U	R.GQYGILDEK.G
10022	456 - 477	809.7465	2426.2177	2426.2230	-2.18	1	26	0.0024	1	U	R.GQYGILDEKGQVIPGGEWDPIR.T
10024	456 - 477	809.7468	2426.2187	2426.2230	-1.80	1	15	0.03	1	U	R.GQYGILDEKGQVIPGGEWDPIR.T
10025	456 - 477	607.5620	2426.2187	2426.2230	-1.78	1	8	0.15	1	U	R.GQYGILDEKGQVIPGGEWDPIR.T
10026	456 - 477	1214.1171	2426.2196	2426.2230	-1.42	1	64	4e-007	1	U	R.GQYGILDEKGQVIPGGEWDPIR.T
10028	456 - 477	809.7479	2426.2218	2426.2230	-0.52	1	5	0.32	1	U	R.GQYGILDEKGQVIPGGEWDPIR.T
10029	456 - 477	809.7480	2426.2223	2426.2230	-0.29	1	8	0.18	1	U	R.GQYGILDEKGQVIPGGEWDPIR.T
10030	456 - 477	809.7485	2426.2236	2426.2230	0.24	1	5	0.35	1	U	R.GQYGILDEKGQVIPGGEWDPIR.T
10031	456 - 477	809.7487	2426.2241	2426.2230	0.46	1	19	0.013	1	U	R.GQYGILDEKGQVIPGGEWDPIR.T
10032	456 - 477	809.7489	2426.2249	2426.2230	0.76	1	5	0.35	1	U	R.GQYGILDEKGQVIPGGEWDPIR.T
10033	456 - 477	809.7489	2426.2249	2426.2230	0.76	1	2	0.66	1	U	R.GQYGILDEKGQVIPGGEWDPIR.T
10034	456 - 477	809.7496	2426.2271	2426.2230	1.67	1	1	0.85	1	U	R.GQYGILDEKGQVIPGGEWDPIR.T
10035	456 - 477	809.7497	2426.2273	2426.2230	1.75	1	6	0.25	1	U	R.GQYGILDEKGQVIPGGEWDPIR.T
10036	456 - 477	809.7497	2426.2273	2426.2230	1.75	1	29	0.0014	1	U	R.GQYGILDEKGQVIPGGEWDPIR.T
4431	465 - 477	712.3668	1422.7191	1422.7256	-4.56	0	68	1.5e-007	1	U	K.GQVIPGGEWDPIR.T
4432	465 - 477	712.3669	1422.7192	1422.7256	-4.47	0	31	0.00079	1	U	K.GQVIPGGEWDPIR.T
4433	465 - 477	712.3680	1422.7215	1422.7256	-2.84	0	21	0.0072	1	U	K.GQVIPGGEWDPIR.T
4434	465 - 477	712.3690	1422.7235	1422.7256	-1.47	0	3	0.48	1	U	K.GQVIPGGEWDPIR.T
4435	465 - 477	712.3691	1422.7237	1422.7256	-1.29	0	9	0.14	1	U	K.GQVIPGGEWDPIR.T
4436	465 - 477	712.3693	1422.7241	1422.7256	-1.04	0	8	0.15	1	U	K.GQVIPGGEWDPIR.T
4437	465 - 477	712.3694	1422.7243	1422.7256	-0.87	0	12	0.058	1	U	K.GQVIPGGEWDPIR.T
4438	465 - 477	712.3698	1422.7250	1422.7256	-0.44	0	3	0.54	1	U	K.GQVIPGGEWDPIR.T
4439	465 - 477	712.3700	1422.7254	1422.7256	-0.093	0	11	0.075	1	U	K.GQVIPGGEWDPIR.T
4440	465 - 477	475.2491	1422.7256	1422.7256	0.027	0	42	5.8e-005	1	U	K.GQVIPGGEWDPIR.T
4441	465 - 477	712.3701	1422.7257	1422.7256	0.078	0	15	0.035	1	U	K.GQVIPGGEWDPIR.T
4442	465 - 477	712.3702	1422.7258	1422.7256	0.16	0	8	0.15	1	U	K.GQVIPGGEWDPIR.T
4443	465 - 477	712.3702	1422.7258	1422.7256	0.16	0	6	0.24	1	U	K.GQVIPGGEWDPIR.T
4444	465 - 477	712.3702	1422.7258	1422.7256	0.16	0	41	7.2e-005	1	U	K.GQVIPGGEWDPIR.T
4445	465 - 477	712.3702	1422.7259	1422.7256	0.25	0	1	0.78	1	U	K.GQVIPGGEWDPIR.T
4446	465 - 477	712.3703	1422.7260	1422.7256	0.34	0	6	0.27	1	U	K.GQVIPGGEWDPIR.T
4447	465 - 477	712.3705	1422.7264	1422.7256	0.59	0	17	0.019	1	U	K.GQVIPGGEWDPIR.T
4448	465 - 477	712.3706	1422.7267	1422.7256	0.76	0	11	0.074	1	U	K.GQVIPGGEWDPIR.T
4450	465 - 477	712.3707	1422.7268	1422.7256	0.85	0	7	0.21	1	U	K.GQVIPGGEWDPIR.T
4451	465 - 477	712.3707	1422.7269	1422.7256	0.94	0	12	0.063	1	U	K.GQVIPGGEWDPIR.T
4452	465 - 477	712.3709	1422.7271	1422.7256	1.11	0	3	0.47	1	U	K.GQVIPGGEWDPIR.T
4453	465 - 477	712.3709	1422.7271	1422.7256	1.11	0	13	0.047	1	U	K.GQVIPGGEWDPIR.T
4454	465 - 477	712.3709	1422.7273	1422.7256	1.19	0	8	0.14	1	U	K.GQVIPGGEWDPIR.T
4455	465 - 477	712.3710	1422.7275	1422.7256	1.37	0	7	0.19	1	U	K.GQVIPGGEWDPIR.T
4456	465 - 477	712.3712	1422.7278	1422.7256	1.54	0	8	0.18	1	U	K.GQVIPGGEWDPIR.T
4459	465 - 477	712.3716	1422.7287	1422.7256	2.22	0	7	0.18	1	U	K.GQVIPGGEWDPIR.T
4460	465 - 477	712.3717	1422.7289	1422.7256	2.31	0	10	0.1	1	U	K.GQVIPGGEWDPIR.T
4461	465 - 477	712.3717	1422.7289	1422.7256	2.31	0	12	0.065	1	U	K.GQVIPGGEWDPIR.T
4462	465 - 477	712.3722	1422.7298	1422.7256	2.99	0	14	0.044	1	U	K.GQVIPGGEWDPIR.T
13510	465 - 495	792.1688	3164.6462	3164.6466	-0.13	1	17	0.019	1	U	K.GQVIPGGEWDPIRTNIDAVSGSLTLNLGTGK.D
13511	465 - 495	1055.8906	3164.6500	3164.6466	1.09	1	17	0.018	1	U	K.GQVIPGGEWDPIRTNIDAVSGSLTLNLGTGK.D
15263	465 - 500	1255.9895	3764.9467	3764.9333	3.54	2	23	0.0055	1	U	K.GQVIPGGEWDPIRTNIDAVSGSLTLNLGTGKDSLER.R
6011	478 - 495	880.9713	1759.9281	1759.9316	-2.00	0	114	4.1e-012	1	U	R.TNIDAVSGSLTLNLGTGK.D
6012	478 - 495	880.9716	1759.9286	1759.9316	-1.72	0	100	9.5e-011	1	U	R.TNIDAVSGSLTLNLGTGK.D
6013	478 - 495	880.9717	1759.9289	1759.9316	-1.51	0	106	2.6e-011	1	U	R.TNIDAVSGSLTLNLGTGK.D
6014	478 - 495	880.9718	1759.9291	1759.9316	-1.44	0	107	1.8e-011	1	U	R.TNIDAVSGSLTLNLGTGK.D
6015	478 - 495	880.9722	1759.9298	1759.9316	-1.03	0	122	7e-013	1	U	R.TNIDAVSGSLTLNLGTGK.D
6016	478 - 495	880.9722	1759.9298	1759.9316	-1.03	0	100	9.6e-011	1	U	R.TNIDAVSGSLTLNLGTGK.D
6017	478 - 495	880.9722	1759.9299	1759.9316	-0.96	0	113	4.9e-012	1	U	R.TNIDAVSGSLTLNLGTGK.D
6018	478 - 495	587.6506	1759.9301	1759.9316	-0.86	0	57	1.9e-006	1	U	R.TNIDAVSGSLTLNLGTGK.D

Query	Start - End	Observed	Mr(expt)	Mr(calc)	ppm	M	Score	Expect	Rank	U	Peptide
6019	478 - 495	880.9724	1759.9303	1759.9316	-0.75	0	97	2.1e-010	1	U	R.TNIDAVSGSLTLNLGTGK.D
6020	478 - 495	587.6509	1759.9308	1759.9316	-0.45	0	26	0.0025	1	U	R.TNIDAVSGSLTLNLGTGK.D
6021	478 - 495	880.9727	1759.9309	1759.9316	-0.41	0	106	2.6e-011	1	U	R.TNIDAVSGSLTLNLGTGK.D
6022	478 - 495	587.6509	1759.9310	1759.9316	-0.34	0	28	0.0016	1	U	R.TNIDAVSGSLTLNLGTGK.D
6023	478 - 495	880.9728	1759.9311	1759.9316	-0.27	0	125	3e-013	1	U	R.TNIDAVSGSLTLNLGTGK.D
6024	478 - 495	587.6510	1759.9312	1759.9316	-0.24	0	57	2.2e-006	1	U	R.TNIDAVSGSLTLNLGTGK.D
6025	478 - 495	587.6510	1759.9312	1759.9316	-0.24	0	37	0.00021	1	U	R.TNIDAVSGSLTLNLGTGK.D
6026	478 - 495	587.6511	1759.9314	1759.9316	-0.13	0	62	6e-007	1	U	R.TNIDAVSGSLTLNLGTGK.D
6027	478 - 495	880.9730	1759.9314	1759.9316	-0.13	0	103	4.9e-011	1	U	R.TNIDAVSGSLTLNLGTGK.D
6028	478 - 495	880.9730	1759.9314	1759.9316	-0.13	0	108	1.4e-011	1	U	R.TNIDAVSGSLTLNLGTGK.D
6029	478 - 495	880.9730	1759.9315	1759.9316	-0.059	0	106	2.7e-011	1	U	R.TNIDAVSGSLTLNLGTGK.D
6030	478 - 495	880.9730	1759.9315	1759.9316	-0.059	0	90	1.1e-009	1	U	R.TNIDAVSGSLTLNLGTGK.D
6031	478 - 495	880.9730	1759.9315	1759.9316	-0.059	0	115	3e-012	1	U	R.TNIDAVSGSLTLNLGTGK.D
6032	478 - 495	587.6512	1759.9317	1759.9316	0.073	0	25	0.0032	1	U	R.TNIDAVSGSLTLNLGTGK.D
6033	478 - 495	587.6512	1759.9317	1759.9316	0.073	0	28	0.0015	1	U	R.TNIDAVSGSLTLNLGTGK.D
6034	478 - 495	587.6512	1759.9317	1759.9316	0.073	0	24	0.0038	1	U	R.TNIDAVSGSLTLNLGTGK.D
6035	478 - 495	587.6512	1759.9317	1759.9316	0.073	0	32	0.00066	1	U	R.TNIDAVSGSLTLNLGTGK.D
6036	478 - 495	880.9731	1759.9317	1759.9316	0.081	0	114	4e-012	1	U	R.TNIDAVSGSLTLNLGTGK.D
6037	478 - 495	880.9731	1759.9317	1759.9316	0.081	0	102	5.6e-011	1	U	R.TNIDAVSGSLTLNLGTGK.D
6038	478 - 495	880.9732	1759.9319	1759.9316	0.15	0	100	9.5e-011	1	U	R.TNIDAVSGSLTLNLGTGK.D
6039	478 - 495	880.9732	1759.9319	1759.9316	0.15	0	70	1e-007	1	U	R.TNIDAVSGSLTLNLGTGK.D
6040	478 - 495	880.9732	1759.9319	1759.9316	0.15	0	113	4.5e-012	1	U	R.TNIDAVSGSLTLNLGTGK.D
6041	478 - 495	587.6512	1759.9319	1759.9316	0.18	0	50	9.7e-006	1	U	R.TNIDAVSGSLTLNLGTGK.D
6042	478 - 495	880.9733	1759.9320	1759.9316	0.22	0	91	7.3e-010	1	U	R.TNIDAVSGSLTLNLGTGK.D
6043	478 - 495	880.9733	1759.9320	1759.9316	0.22	0	104	4e-011	1	U	R.TNIDAVSGSLTLNLGTGK.D
6044	478 - 495	880.9733	1759.9320	1759.9316	0.22	0	101	8.6e-011	1	U	R.TNIDAVSGSLTLNLGTGK.D
6045	478 - 495	880.9733	1759.9320	1759.9316	0.22	0	102	6.5e-011	1	U	R.TNIDAVSGSLTLNLGTGK.D
6046	478 - 495	587.6513	1759.9321	1759.9316	0.28	0	40	9.6e-005	1	U	R.TNIDAVSGSLTLNLGTGK.D
6047	478 - 495	880.9733	1759.9321	1759.9316	0.29	0	129	1.1e-013	1	U	R.TNIDAVSGSLTLNLGTGK.D
6048	478 - 495	880.9734	1759.9322	1759.9316	0.36	0	120	1.1e-012	1	U	R.TNIDAVSGSLTLNLGTGK.D
6049	478 - 495	587.6514	1759.9323	1759.9316	0.39	0	33	0.00048	1	U	R.TNIDAVSGSLTLNLGTGK.D
6050	478 - 495	587.6514	1759.9323	1759.9316	0.39	0	47	2e-005	1	U	R.TNIDAVSGSLTLNLGTGK.D
6051	478 - 495	587.6514	1759.9325	1759.9316	0.49	0	32	0.00067	1	U	R.TNIDAVSGSLTLNLGTGK.D
6052	478 - 495	587.6514	1759.9325	1759.9316	0.49	0	25	0.0029	1	U	R.TNIDAVSGSLTLNLGTGK.D
6053	478 - 495	587.6514	1759.9325	1759.9316	0.49	0	66	2.6e-007	1	U	R.TNIDAVSGSLTLNLGTGK.D
6054	478 - 495	587.6514	1759.9325	1759.9316	0.49	0	35	0.00029	1	U	R.TNIDAVSGSLTLNLGTGK.D
6055	478 - 495	587.6514	1759.9325	1759.9316	0.49	0	38	0.00018	1	U	R.TNIDAVSGSLTLNLGTGK.D
6056	478 - 495	587.6514	1759.9325	1759.9316	0.49	0	27	0.0019	1	U	R.TNIDAVSGSLTLNLGTGK.D
6057	478 - 495	587.6514	1759.9325	1759.9316	0.49	0	44	3.8e-005	1	U	R.TNIDAVSGSLTLNLGTGK.D
6058	478 - 495	880.9735	1759.9325	1759.9316	0.50	0	100	1e-010	1	U	R.TNIDAVSGSLTLNLGTGK.D
6059	478 - 495	880.9736	1759.9326	1759.9316	0.57	0	116	2.6e-012	1	U	R.TNIDAVSGSLTLNLGTGK.D
6060	478 - 495	880.9736	1759.9326	1759.9316	0.57	0	101	8.1e-011	1	U	R.TNIDAVSGSLTLNLGTGK.D
6061	478 - 495	587.6515	1759.9326	1759.9316	0.59	0	34	0.00037	1	U	R.TNIDAVSGSLTLNLGTGK.D
6062	478 - 495	587.6515	1759.9326	1759.9316	0.59	0	34	0.0004	1	U	R.TNIDAVSGSLTLNLGTGK.D
6063	478 - 495	880.9736	1759.9327	1759.9316	0.64	0	116	2.7e-012	1	U	R.TNIDAVSGSLTLNLGTGK.D
6064	478 - 495	880.9736	1759.9327	1759.9316	0.64	0	115	3.4e-012	1	U	R.TNIDAVSGSLTLNLGTGK.D
6065	478 - 495	880.9736	1759.9327	1759.9316	0.64	0	108	1.5e-011	1	U	R.TNIDAVSGSLTLNLGTGK.D
6066	478 - 495	587.6516	1759.9328	1759.9316	0.70	0	46	2.5e-005	1	U	R.TNIDAVSGSLTLNLGTGK.D
6067	478 - 495	587.6516	1759.9328	1759.9316	0.70	0	40	0.00011	1	U	R.TNIDAVSGSLTLNLGTGK.D
6068	478 - 495	880.9737	1759.9328	1759.9316	0.71	0	109	1.3e-011	1	U	R.TNIDAVSGSLTLNLGTGK.D
6069	478 - 495	880.9737	1759.9328	1759.9316	0.71	0	123	5.2e-013	1	U	R.TNIDAVSGSLTLNLGTGK.D
6070	478 - 495	880.9738	1759.9330	1759.9316	0.77	0	110	9.7e-012	1	U	R.TNIDAVSGSLTLNLGTGK.D
6071	478 - 495	587.6516	1759.9330	1759.9316	0.80	0	27	0.0021	1	U	R.TNIDAVSGSLTLNLGTGK.D
6072	478 - 495	587.6516	1759.9330	1759.9316	0.80	0	40	9.1e-005	1	U	R.TNIDAVSGSLTLNLGTGK.D
6073	478 - 495	880.9738	1759.9331	1759.9316	0.84	0	110	9.6e-012	1	U	R.TNIDAVSGSLTLNLGTGK.D
6074	478 - 495	880.9738	1759.9331	1759.9316	0.84	0	116	2.5e-012	1	U	R.TNIDAVSGSLTLNLGTGK.D
6075	478 - 495	880.9739	1759.9333	1759.9316	0.98	0	99	1.1e-010	1	U	R.TNIDAVSGSLTLNLGTGK.D
6076	478 - 495	880.9739	1759.9333	1759.9316	0.98	0	102	6.5e-011	1	U	R.TNIDAVSGSLTLNLGTGK.D
6077	478 - 495	587.6517	1759.9334	1759.9316	1.01	0	29	0.0013	1	U	R.TNIDAVSGSLTLNLGTGK.D
6078	478 - 495	587.6517	1759.9334	1759.9316	1.01	0	58	1.5e-006	1	U	R.TNIDAVSGSLTLNLGTGK.D
6079	478 - 495	587.6517	1759.9334	1759.9316	1.01	0	43	5.4e-005	1	U	R.TNIDAVSGSLTLNLGTGK.D
6080	478 - 495	587.6518	1759.9336	1759.9316	1.11	0	21	0.0087	1	U	R.TNIDAVSGSLTLNLGTGK.D
6081	478 - 495	880.9741	1759.9337	1759.9316	1.19	0	99	1.3e-010	1	U	R.TNIDAVSGSLTLNLGTGK.D
6082	478 - 495	880.9741	1759.9337	1759.9316	1.19	0	104	3.7e-011	1	U	R.TNIDAVSGSLTLNLGTGK.D
6083	478 - 495	587.6519	1759.9337	1759.9316	1.22	0	15	0.033	1	U	R.TNIDAVSGSLTLNLGTGK.D
6084	478 - 495	587.6519	1759.9337	1759.9316	1.22	0	30	0.00096	1	U	R.TNIDAVSGSLTLNLGTGK.D
6085	478 - 495	587.6519	1759.9339	1759.9316	1.32	0	23	0.0046	1	U	R.TNIDAVSGSLTLNLGTGK.D
6086	478 - 495	587.6519	1759.9339	1759.9316	1.32	0	12	0.058	1	U	R.TNIDAVSGSLTLNLGTGK.D
6087	478 - 495	587.6519	1759.9339	1759.9316	1.32	0	27	0.002	1	U	R.TNIDAVSGSLTLNLGTGK.D
6088	478 - 495	587.6519	1759.9339	1759.9316	1.32	0	62	6.4e-007	1	U	R.TNIDAVSGSLTLNLGTGK.D
6089	478 - 495	880.9742	1759.9339	1759.9316	1.33	0	78	1.6e-008	1	U	R.TNIDAVSGSLTLNLGTGK.D
6090	478 - 495	880.9744	1759.9342	1759.9316	1.47	0	103	5.6e-011	1	U	R.TNIDAVSGSLTLNLGTGK.D
6091	478 - 495	880.9744	1759.9342	1759.9316	1.47	0	101	7.7e-011	1	U	R.TNIDAVSGSLTLNLGTGK.D
6092	478 - 495	880.9745	1759.9344	1759.9316	1.61	0	104	3.6e-011	1	U	R.TNIDAVSGSLTLNLGTGK.D
6093	478 - 495	587.6521	1759.9345	1759.9316	1.63	0	31	0.00077	1	U	R.TNIDAVSGSLTLNLGTGK.D
6094	478 - 495	880.9747	1759.9348	1759.9316	1.81	0	91	7.6e-010	1	U	R.TNIDAVSGSLTLNLGTGK.D
6095	478 - 495	587.6522	1759.9348	1759.9316	1.84	0	7	0.2	1	U	R.TNIDAVSGSLTLNLGTGK.D
6096	478 - 495	587.6523	1759.9350	1759.9316	1.95	0	14	0.036	1	U	R.TNIDAVSGSLTLNLGTGK.D
6097	478 - 495	880.9748	1759.9350	1759.9316	1.95	0	91	7.8e-010	1	U	R.TNIDAVSGSLTLNLGTGK.D
6098	478 - 495	587.6524	1759.9354	1759.9316	2.15	0	24	0.0042	1	U	R.TNIDAVSGSLTLNLGTGK.D
6099	478 - 495	880.9756	1759.9367	1759.9316	2.92	0	136	2.5e-014	1	U	R.TNIDAVSGSLTLNLGTGK.D
6100	478 - 495	880.9761	1759.9377	1759.9316	3.48	0	65	3.2e-007	1	U	R.TNIDAVSGSLTLNLGTGK.D
9672	478 - 500	787.7455	2360.2148	2360.2183	-1.49	1	18	0.016	1	U	R.TNIDAVSGSLTLNLGTGKDSLER.R
9673	478 - 500	787.7456	2360.2150	2360.2183	-1.41	1	16	0.025	1	U	R.TNIDAVSGSLTLNLGTGKDSLER.R
9674	478 - 500	787.7460	2360.2161	2360.2183	-0.95	1	36	0.00023	1	U	R.TNIDAVSGSLTLNLGTGKDSLER.R
9675	478 - 500	787.7462	2360.2168	2360.2183	-0.64	1	17	0.02	1	U	R.TNIDAVSGSLTLNLGTGKDSLER.R
9676	478 - 500	787.7463	2360.2170	2360.2183	-0.56	1	22	0.0062	1	U	R.TNIDAVSGSLTLNLGTGKDSLER.R
9677	478 - 500	787.7465	2360.2176	2360.2183	-0.33	1	27	0.0019	1	U	R.TNIDAVSGSLTLNLGTGKDSLER.R

Query	Start - End	Observed	Mr(expt)	Mr(calc)	ppm	M	Score	Expect	Rank	U	Peptide
9678	478 - 500	787.7466	2360.2179	2360.2183	-0.17	1	10	0.11	1	U	R.TNIDAVSGSLTLNLGTGKDSLER.R
9679	478 - 500	787.7466	2360.2179	2360.2183	-0.17	1	13	0.056	1	U	R.TNIDAVSGSLTLNLGTGKDSLER.R
9680	478 - 500	787.7466	2360.2181	2360.2183	-0.094	1	17	0.019	1	U	R.TNIDAVSGSLTLNLGTGKDSLER.R
9681	478 - 500	787.7467	2360.2183	2360.2183	-0.017	1	19	0.013	1	U	R.TNIDAVSGSLTLNLGTGKDSLER.R
9682	478 - 500	787.7467	2360.2183	2360.2183	-0.017	1	13	0.053	1	U	R.TNIDAVSGSLTLNLGTGKDSLER.R
9683	478 - 500	787.7468	2360.2185	2360.2183	0.061	1	10	0.095	1	U	R.TNIDAVSGSLTLNLGTGKDSLER.R
9684	478 - 500	787.7468	2360.2185	2360.2183	0.061	1	14	0.036	1	U	R.TNIDAVSGSLTLNLGTGKDSLER.R
9685	478 - 500	787.7468	2360.2185	2360.2183	0.061	1	18	0.016	1	U	R.TNIDAVSGSLTLNLGTGKDSLER.R
9686	478 - 500	787.7469	2360.2188	2360.2183	0.22	1	47	1.9e-005	1	U	R.TNIDAVSGSLTLNLGTGKDSLER.R
9687	478 - 500	787.7471	2360.2194	2360.2183	0.45	1	13	0.051	1	U	R.TNIDAVSGSLTLNLGTGKDSLER.R
9688	478 - 500	787.7471	2360.2196	2360.2183	0.53	1	20	0.0096	1	U	R.TNIDAVSGSLTLNLGTGKDSLER.R
9689	478 - 500	787.7472	2360.2197	2360.2183	0.60	1	5	0.31	1	U	R.TNIDAVSGSLTLNLGTGKDSLER.R
9690	478 - 500	1181.1172	2360.2198	2360.2183	0.64	1	85	3.2e-009	1	U	R.TNIDAVSGSLTLNLGTGKDSLER.R
9691	478 - 500	591.0623	2360.2199	2360.2183	0.68	1	33	0.00056	1	U	R.TNIDAVSGSLTLNLGTGKDSLER.R
9692	478 - 500	787.7473	2360.2199	2360.2183	0.68	1	13	0.051	1	U	R.TNIDAVSGSLTLNLGTGKDSLER.R
9693	478 - 500	787.7473	2360.2199	2360.2183	0.68	1	32	0.00064	1	U	R.TNIDAVSGSLTLNLGTGKDSLER.R
9694	478 - 500	787.7474	2360.2203	2360.2183	0.84	1	24	0.0042	1	U	R.TNIDAVSGSLTLNLGTGKDSLER.R
9695	478 - 500	787.7476	2360.2210	2360.2183	1.15	1	46	2.6e-005	1	U	R.TNIDAVSGSLTLNLGTGKDSLER.R
9696	478 - 500	787.7477	2360.2212	2360.2183	1.23	1	7	0.19	1	U	R.TNIDAVSGSLTLNLGTGKDSLER.R
9697	478 - 500	787.7477	2360.2212	2360.2183	1.23	1	18	0.014	1	U	R.TNIDAVSGSLTLNLGTGKDSLER.R
9698	478 - 500	787.7477	2360.2214	2360.2183	1.30	1	10	0.1	1	U	R.TNIDAVSGSLTLNLGTGKDSLER.R
9699	478 - 500	787.7478	2360.2216	2360.2183	1.38	1	14	0.036	1	U	R.TNIDAVSGSLTLNLGTGKDSLER.R
9700	478 - 500	787.7478	2360.2216	2360.2183	1.38	1	20	0.011	1	U	R.TNIDAVSGSLTLNLGTGKDSLER.R
9701	478 - 500	787.7479	2360.2218	2360.2183	1.46	1	16	0.023	1	U	R.TNIDAVSGSLTLNLGTGKDSLER.R
9702	478 - 500	787.7482	2360.2229	2360.2183	1.92	1	25	0.0032	1	U	R.TNIDAVSGSLTLNLGTGKDSLER.R
9703	478 - 500	787.7484	2360.2232	2360.2183	2.08	1	21	0.0086	1	U	R.TNIDAVSGSLTLNLGTGKDSLER.R
9704	478 - 500	787.7484	2360.2232	2360.2183	2.08	1	28	0.0015	1	U	R.TNIDAVSGSLTLNLGTGKDSLER.R
9705	478 - 500	787.7484	2360.2234	2360.2183	2.16	1	21	0.0075	1	U	R.TNIDAVSGSLTLNLGTGKDSLER.R
9706	478 - 500	787.7485	2360.2236	2360.2183	2.23	1	8	0.16	1	U	R.TNIDAVSGSLTLNLGTGKDSLER.R
9707	478 - 500	787.7486	2360.2240	2360.2183	2.39	1	19	0.012	1	U	R.TNIDAVSGSLTLNLGTGKDSLER.R
9708	478 - 500	787.7487	2360.2243	2360.2183	2.54	1	13	0.048	1	U	R.TNIDAVSGSLTLNLGTGKDSLER.R
9709	478 - 500	787.7488	2360.2247	2360.2183	2.70	1	15	0.029	1	U	R.TNIDAVSGSLTLNLGTGKDSLER.R
9710	478 - 500	1181.1219	2360.2293	2360.2183	4.67	1	41	8e-005	1	U	R.TNIDAVSGSLTLNLGTGKDSLER.R
235	496 - 500	310.1559	618.2973	618.2973	-0.018	0	27	0.0018	1	U	K.DSLER.R
236	496 - 500	310.1559	618.2973	618.2973	-0.018	0	25	0.0031	1	U	K.DSLER.R
237	496 - 500	310.1559	618.2973	618.2973	0.082	0	21	0.0073	1	U	K.DSLER.R
238	496 - 500	310.1560	618.2975	618.2973	0.38	0	18	0.016	1	U	K.DSLER.R
239	496 - 500	310.1561	618.2977	618.2973	0.67	0	19	0.013	1	U	K.DSLER.R
665	496 - 501	388.2065	774.3984	774.3984	0.026	1	10	0.1	1	U	K.DSLERR.V
666	496 - 501	388.2066	774.3986	774.3984	0.26	1	13	0.046	1	U	K.DSLERR.V
111	501 - 505	272.6819	543.3492	543.3493	-0.074	1	20	0.011	1	U	R.RVAAK.N
112	501 - 505	272.6819	543.3493	543.3493	0.037	1	6	0.25	1	U	R.RVAAK.N
8702	502 - 520	710.6968	2129.0685	2129.0674	0.52	2	19	0.011	1	U	R.VAAKNMNDPEDKTPEITIK.E + Oxidation (M)
1810	506 - 513	489.6952	977.3758	977.3760	-0.24	0	15	0.03	1	U	K.NMNDPEDK.T + Oxidation (M)
1811	506 - 513	489.6953	977.3760	977.3760	0.0051	0	15	0.029	1	U	K.NMNDPEDK.T + Oxidation (M)
1812	506 - 513	489.6954	977.3763	977.3760	0.32	0	20	0.0094	1	U	K.NMNDPEDK.T + Oxidation (M)
1813	506 - 513	489.6956	977.3766	977.3760	0.57	0	19	0.013	1	U	K.NMNDPEDK.T + Oxidation (M)
5944	506 - 520	582.2851	1743.8335	1743.8349	-0.82	1	36	0.00023	1	U	K.NMNDPEDKTPEITIK.E
5945	506 - 520	582.2855	1743.8347	1743.8349	-0.084	1	33	0.00045	1	U	K.NMNDPEDKTPEITIK.E
5946	506 - 520	582.2856	1743.8351	1743.8349	0.13	1	4	0.4	1	U	K.NMNDPEDKTPEITIK.E
5947	506 - 520	872.9254	1743.8362	1743.8349	0.73	1	85	3.4e-009	1	U	K.NMNDPEDKTPEITIK.E
5997	506 - 520	880.9209	1759.8272	1759.8298	-1.45	1	57	1.9e-006	1	U	K.NMNDPEDKTPEITIK.E + Oxidation (M)
5998	506 - 520	587.6165	1759.8277	1759.8298	-1.18	1	38	0.00015	1	U	K.NMNDPEDKTPEITIK.E + Oxidation (M)
5999	506 - 520	587.6169	1759.8290	1759.8298	-0.46	1	41	7.5e-005	1	U	K.NMNDPEDKTPEITIK.E + Oxidation (M)
6001	506 - 520	587.6169	1759.8290	1759.8298	-0.46	1	36	0.00026	1	U	K.NMNDPEDKTPEITIK.E + Oxidation (M)
6002	506 - 520	880.9219	1759.8292	1759.8298	-0.34	1	64	3.7e-007	1	U	K.NMNDPEDKTPEITIK.E + Oxidation (M)
6003	506 - 520	880.9222	1759.8298	1759.8298	0.0028	1	50	1e-005	1	U	K.NMNDPEDKTPEITIK.E + Oxidation (M)
6004	506 - 520	880.9224	1759.8303	1759.8298	0.28	1	19	0.012	1	U	K.NMNDPEDKTPEITIK.E + Oxidation (M)
6005	506 - 520	587.6176	1759.8310	1759.8298	0.69	1	38	0.00014	1	U	K.NMNDPEDKTPEITIK.E + Oxidation (M)
6006	506 - 520	587.6181	1759.8325	1759.8298	1.52	1	43	4.5e-005	1	U	K.NMNDPEDKTPEITIK.E + Oxidation (M)
6007	506 - 520	587.6183	1759.8330	1759.8298	1.83	1	3	0.55	1	U	K.NMNDPEDKTPEITIK.E + Oxidation (M)
6008	506 - 520	587.6183	1759.8330	1759.8298	1.83	1	36	0.00028	1	U	K.NMNDPEDKTPEITIK.E + Oxidation (M)
6009	506 - 520	587.6184	1759.8334	1759.8298	2.04	1	17	0.02	1	U	K.NMNDPEDKTPEITIK.E + Oxidation (M)
781	514 - 520	401.2390	800.4635	800.4644	-1.10	0	17	0.02	1	U	K.TPEITIK.E
782	514 - 520	401.2394	800.4643	800.4644	-0.031	0	5	0.33	1	U	K.TPEITIK.E
783	514 - 520	401.2394	800.4643	800.4644	-0.031	0	5	0.32	1	U	K.TPEITIK.E
180	521 - 525	294.6893	587.3641	587.3642	-0.30	1	16	0.025	1	U	K.EAIKK.A
181	521 - 525	294.6893	587.3641	587.3642	-0.30	1	15	0.033	1	U	K.EAIKK.A
182	521 - 525	294.6894	587.3642	587.3642	0.0085	1	17	0.02	1	U	K.EAIKK.A
1490	525 - 532	468.2507	934.4869	934.4872	-0.37	1	18	0.016	1	U	K.KAFNAQEK.D
1491	525 - 532	468.2507	934.4869	934.4872	-0.37	1	22	0.0057	1	U	K.KAFNAQEK.D
1492	525 - 532	468.2510	934.4875	934.4872	0.28	1	49	1.1e-005	1	U	K.KAFNAQEK.D
1493	525 - 532	468.2511	934.4877	934.4872	0.54	1	39	0.00011	1	U	K.KAFNAQEK.D
3626	525 - 535	421.8861	1262.6366	1262.6367	-0.10	2	22	0.0067	1	U	K.KAFNAQEKDGR.L
3628	525 - 535	632.3261	1262.6377	1262.6367	0.75	2	48	1.5e-005	1	U	K.KAFNAQEKDGR.L
804	526 - 532	404.2033	806.3921	806.3922	-0.14	0	29	0.0013	1	U	K.AFNAQEK.D
805	526 - 532	404.2034	806.3922	806.3922	-0.069	0	28	0.0016	1	U	K.AFNAQEK.D
807	526 - 532	404.2038	806.3930	806.3922	0.92	0	17	0.021	1	U	K.AFNAQEK.D
808	526 - 532	404.2039	806.3933	806.3922	1.29	0	20	0.01	1	U	K.AFNAQEK.D
2731	526 - 535	379.1873	1134.5401	1134.5418	-1.45	1	15	0.032	1	U	K.AFNAQEKDGR.L
2732	526 - 535	379.1876	1134.5409	1134.5418	-0.80	1	10	0.11	1	U	K.AFNAQEKDGR.L
2733	526 - 535	568.2779	1134.5412	1134.5418	-0.47	1	40	9.4e-005	1	U	K.AFNAQEKDGR.L
2734	526 - 535	379.1879	1134.5418	1134.5418	0.0062	1	11	0.085	1	U	K.AFNAQEKDGR.L
2735	526 - 535	379.1879	1134.5419	1134.5418	0.088	1	12	0.065	1	U	K.AFNAQEKDGR.L
2736	526 - 535	568.2784	1134.5422	1134.5418	0.39	1	28	0.0015	1	U	K.AFNAQEKDGR.L
2737	526 - 535	568.2784	1134.5422	1134.5418	0.39	1	47	2.2e-005	1	U	K.AFNAQEKDGR.L
2738	526 - 535	568.2784	1134.5423	1134.5418	0.50	1	44	3.7e-005	1	U	K.AFNAQEKDGR.L
2739	526 - 535	379.1881	1134.5424	1134.5418	0.57	1	1	0.83	1	U	K.AFNAQEKDGR.L

Query	Start - End	Observed	Mr(expt)	Mr(calc)	ppm	M	Score	Expect	Rank	U	Peptide
2612	536 - 544	558.7637	1115.5128	1115.5135	-0.62	0	28	0.0017	1	U	R.LYYTDQGEK.D
2613	536 - 544	558.7641	1115.5136	1115.5135	0.14	0	33	0.00051	1	U	R.LYYTDQGEK.D
2614	536 - 544	558.7646	1115.5146	1115.5135	1.02	0	24	0.004	1	U	R.LYYTDQGEK.D
2615	536 - 544	558.7650	1115.5155	1115.5135	1.78	0	46	2.2e-005	1	U	R.LYYTDQGEK.D
13549	536 - 562	1587.7690	3173.5235	3173.5292	-1.78	1	49	1.3e-005	1	U	R.LYYTDQGEKDIFIDEPSINLITDENTK.K
13550	536 - 562	1058.8490	3173.5252	3173.5292	-1.27	1	48	1.8e-005	1	U	R.LYYTDQGEKDIFIDEPSINLITDENTK.K
13551	536 - 562	1058.8492	3173.5259	3173.5292	-1.04	1	17	0.022	1	U	R.LYYTDQGEKDIFIDEPSINLITDENTK.K
13552	536 - 562	1058.8494	3173.5263	3173.5292	-0.92	1	22	0.0065	1	U	R.LYYTDQGEKDIFIDEPSINLITDENTK.K
13553	536 - 562	1058.8496	3173.5270	3173.5292	-0.69	1	11	0.087	1	U	R.LYYTDQGEKDIFIDEPSINLITDENTK.K
13554	536 - 562	1058.8496	3173.5270	3173.5292	-0.69	1	30	0.0011	1	U	R.LYYTDQGEKDIFIDEPSINLITDENTK.K
13555	536 - 562	1058.8497	3173.5274	3173.5292	-0.58	1	29	0.0012	1	U	R.LYYTDQGEKDIFIDEPSINLITDENTK.K
13556	536 - 562	1058.8499	3173.5277	3173.5292	-0.46	1	29	0.0012	1	U	R.LYYTDQGEKDIFIDEPSINLITDENTK.K
13557	536 - 562	1058.8500	3173.5281	3173.5292	-0.34	1	9	0.12	1	U	R.LYYTDQGEKDIFIDEPSINLITDENTK.K
13558	536 - 562	1058.8501	3173.5285	3173.5292	-0.23	1	17	0.021	1	U	R.LYYTDQGEKDIFIDEPSINLITDENTK.K
13559	536 - 562	1058.8501	3173.5285	3173.5292	-0.23	1	13	0.055	1	U	R.LYYTDQGEKDIFIDEPSINLITDENTK.K
13560	536 - 562	1058.8503	3173.5292	3173.5292	0.0025	1	10	0.11	1	U	R.LYYTDQGEKDIFIDEPSINLITDENTK.K
13561	536 - 562	1058.8506	3173.5299	3173.5292	0.23	1	20	0.01	1	U	R.LYYTDQGEKDIFIDEPSINLITDENTK.K
13562	536 - 562	1058.8508	3173.5307	3173.5292	0.46	1	52	5.6e-006	1	U	R.LYYTDQGEKDIFIDEPSINLITDENTK.K
13563	536 - 562	1058.8511	3173.5314	3173.5292	0.69	1	10	0.09	1	U	R.LYYTDQGEKDIFIDEPSINLITDENTK.K
13564	536 - 562	1058.8511	3173.5314	3173.5292	0.69	1	43	5.3e-005	1	U	R.LYYTDQGEKDIFIDEPSINLITDENTK.K
13565	536 - 562	1058.8512	3173.5318	3173.5292	0.81	1	37	0.00021	1	U	R.LYYTDQGEKDIFIDEPSINLITDENTK.K
13566	536 - 562	1058.8516	3173.5329	3173.5292	1.16	1	60	1.1e-006	1	U	R.LYYTDQGEKDIFIDEPSINLITDENTK.K
13567	536 - 562	1058.8517	3173.5332	3173.5292	1.27	1	59	1.3e-006	1	U	R.LYYTDQGEKDIFIDEPSINLITDENTK.K
13568	536 - 562	1058.8519	3173.5340	3173.5292	1.50	1	37	0.00021	1	U	R.LYYTDQGEKDIFIDEPSINLITDENTK.K
13569	536 - 562	1058.8523	3173.5351	3173.5292	1.85	1	31	0.00089	1	U	R.LYYTDQGEKDIFIDEPSINLITDENTK.K
13570	536 - 562	1058.8523	3173.5351	3173.5292	1.85	1	45	2.8e-005	1	U	R.LYYTDQGEKDIFIDEPSINLITDENTK.K
13571	536 - 562	1058.8525	3173.5358	3173.5292	2.08	1	54	4.1e-006	1	U	R.LYYTDQGEKDIFIDEPSINLITDENTK.K
13572	536 - 562	794.3915	3173.5371	3173.5292	2.48	1	59	1.3e-006	1	U	R.LYYTDQGEKDIFIDEPSINLITDENTK.K
13573	536 - 562	1058.8531	3173.5376	3173.5292	2.66	1	7	0.19	1	U	R.LYYTDQGEKDIFIDEPSINLITDENTK.K
13574	536 - 562	1058.8534	3173.5384	3173.5292	2.89	1	11	0.074	1	U	R.LYYTDQGEKDIFIDEPSINLITDENTK.K
13575	536 - 562	1058.8536	3173.5391	3173.5292	3.12	1	25	0.0033	1	U	R.LYYTDQGEKDIFIDEPSINLITDENTK.K
13576	536 - 562	1058.8538	3173.5395	3173.5292	3.23	1	14	0.037	1	U	R.LYYTDQGEKDIFIDEPSINLITDENTK.K
13577	536 - 562	1058.8538	3173.5395	3173.5292	3.23	1	20	0.011	1	U	R.LYYTDQGEKDIFIDEPSINLITDENTK.K
13578	536 - 562	1058.8540	3173.5402	3173.5292	3.46	1	34	0.00044	1	U	R.LYYTDQGEKDIFIDEPSINLITDENTK.K
13579	536 - 562	1058.8542	3173.5409	3173.5292	3.69	1	14	0.041	1	U	R.LYYTDQGEKDIFIDEPSINLITDENTK.K
13580	536 - 562	1058.8545	3173.5416	3173.5292	3.93	1	22	0.0057	1	U	R.LYYTDQGEKDIFIDEPSINLITDENTK.K
13581	536 - 562	1058.8546	3173.5420	3173.5292	4.04	1	17	0.022	1	U	R.LYYTDQGEKDIFIDEPSINLITDENTK.K
13582	536 - 562	1058.8556	3173.5449	3173.5292	4.96	1	13	0.051	1	U	R.LYYTDQGEKDIFIDEPSINLITDENTK.K
14206	536 - 563	1101.5461	3301.6166	3301.6241	-2.29	2	15	0.034	1	U	R.LYYTDQGEKDIFIDEPSINLITDENTKK.E
14207	536 - 563	661.3309	3301.6180	3301.6241	-1.87	2	29	0.0012	1	U	R.LYYTDQGEKDIFIDEPSINLITDENTKK.E
14209	536 - 563	826.4125	3301.6208	3301.6241	-1.01	2	23	0.0051	1	U	R.LYYTDQGEKDIFIDEPSINLITDENTKK.E
14210	536 - 563	826.4130	3301.6230	3301.6241	-0.35	2	38	0.00015	1	U	R.LYYTDQGEKDIFIDEPSINLITDENTKK.E
14211	536 - 563	826.4131	3301.6232	3301.6241	-0.27	2	15	0.032	1	U	R.LYYTDQGEKDIFIDEPSINLITDENTKK.E
14212	536 - 563	826.4131	3301.6235	3301.6241	-0.20	2	31	0.00086	1	U	R.LYYTDQGEKDIFIDEPSINLITDENTKK.E
14213	536 - 563	826.4131	3301.6235	3301.6241	-0.20	2	18	0.016	1	U	R.LYYTDQGEKDIFIDEPSINLITDENTKK.E
14214	536 - 563	826.4133	3301.6240	3301.6241	-0.053	2	8	0.16	1	U	R.LYYTDQGEKDIFIDEPSINLITDENTKK.E
14215	536 - 563	826.4135	3301.6247	3301.6241	0.17	2	27	0.0021	1	U	R.LYYTDQGEKDIFIDEPSINLITDENTKK.E
14216	536 - 563	551.2782	3301.6255	3301.6241	0.42	2	51	7.8e-006	1	U	R.LYYTDQGEKDIFIDEPSINLITDENTKK.E
14217	536 - 563	826.4138	3301.6259	3301.6241	0.54	2	15	0.03	1	U	R.LYYTDQGEKDIFIDEPSINLITDENTKK.E
14218	536 - 563	826.4139	3301.6264	3301.6241	0.69	2	26	0.0023	1	U	R.LYYTDQGEKDIFIDEPSINLITDENTKK.E
14219	536 - 563	826.4139	3301.6267	3301.6241	0.76	2	23	0.005	1	U	R.LYYTDQGEKDIFIDEPSINLITDENTKK.E
14220	536 - 563	826.4140	3301.6269	3301.6241	0.83	2	1	0.77	1	U	R.LYYTDQGEKDIFIDEPSINLITDENTKK.E
14221	536 - 563	826.4140	3301.6269	3301.6241	0.83	2	21	0.0081	1	U	R.LYYTDQGEKDIFIDEPSINLITDENTKK.E
14222	536 - 563	826.4140	3301.6269	3301.6241	0.83	2	33	0.00055	1	U	R.LYYTDQGEKDIFIDEPSINLITDENTKK.E
14223	536 - 563	1651.8209	3301.6273	3301.6241	0.95	2	28	0.0017	1	U	R.LYYTDQGEKDIFIDEPSINLITDENTKK.E
14224	536 - 563	1101.5498	3301.6276	3301.6241	1.04	2	63	5.2e-007	1	U	R.LYYTDQGEKDIFIDEPSINLITDENTKK.E
14225	536 - 563	826.4142	3301.6279	3301.6241	1.13	2	24	0.0038	1	U	R.LYYTDQGEKDIFIDEPSINLITDENTKK.E
14226	536 - 563	826.4144	3301.6286	3301.6241	1.35	2	9	0.13	1	U	R.LYYTDQGEKDIFIDEPSINLITDENTKK.E
14227	536 - 563	826.4145	3301.6289	3301.6241	1.43	2	13	0.051	1	U	R.LYYTDQGEKDIFIDEPSINLITDENTKK.E
14228	536 - 563	826.4147	3301.6298	3301.6241	1.72	2	9	0.13	1	U	R.LYYTDQGEKDIFIDEPSINLITDENTKK.E
14229	536 - 563	826.4147	3301.6298	3301.6241	1.72	2	19	0.012	1	U	R.LYYTDQGEKDIFIDEPSINLITDENTKK.E
14230	536 - 563	826.4147	3301.6298	3301.6241	1.72	2	30	0.00097	1	U	R.LYYTDQGEKDIFIDEPSINLITDENTKK.E
14231	536 - 563	826.4148	3301.6301	3301.6241	1.80	2	23	0.0046	1	U	R.LYYTDQGEKDIFIDEPSINLITDENTKK.E
14232	536 - 563	826.4149	3301.6303	3301.6241	1.87	2	10	0.1	1	U	R.LYYTDQGEKDIFIDEPSINLITDENTKK.E
14233	536 - 563	826.4149	3301.6306	3301.6241	1.94	2	28	0.0015	1	U	R.LYYTDQGEKDIFIDEPSINLITDENTKK.E
14234	536 - 563	826.4149	3301.6306	3301.6241	1.94	2	23	0.0053	1	U	R.LYYTDQGEKDIFIDEPSINLITDENTKK.E
14235	536 - 563	826.4150	3301.6308	3301.6241	2.02	2	17	0.019	1	U	R.LYYTDQGEKDIFIDEPSINLITDENTKK.E
14236	536 - 563	826.4150	3301.6311	3301.6241	2.09	2	25	0.0035	1	U	R.LYYTDQGEKDIFIDEPSINLITDENTKK.E
14237	536 - 563	826.4152	3301.6318	3301.6241	2.31	2	23	0.0051	1	U	R.LYYTDQGEKDIFIDEPSINLITDENTKK.E
14238	536 - 563	826.4153	3301.6323	3301.6241	2.46	2	23	0.0055	1	U	R.LYYTDQGEKDIFIDEPSINLITDENTKK.E
14239	536 - 563	826.4158	3301.6342	3301.6241	3.05	2	15	0.032	1	U	R.LYYTDQGEKDIFIDEPSINLITDENTKK.E
8367	545 - 562	1039.0173	2076.0201	2076.0263	-2.96	0	35	0.00031	1	U	K.DIFIDEPSINLITDENTK.K
8368	545 - 562	1039.0179	2076.0213	2076.0263	-2.37	0	15	0.03	1	U	K.DIFIDEPSINLITDENTK.K
8369	545 - 562	1039.0186	2076.0226	2076.0263	-1.78	0	5	0.33	1	U	K.DIFIDEPSINLITDENTK.K
8370	545 - 562	1039.0194	2076.0243	2076.0263	-0.96	0	10	0.091	1	U	K.DIFIDEPSINLITDENTK.K
8371	545 - 562	1039.0194	2076.0243	2076.0263	-0.96	0	26	0.0026	1	U	K.DIFIDEPSINLITDENTK.K
8372	545 - 562	693.0163	2076.0271	2076.0263	0.39	0	56	2.6e-006	1	U	K.DIFIDEPSINLITDENTK.K
8374	545 - 562	1039.0217	2076.0289	2076.0263	1.27	0	6	0.28	1	U	K.DIFIDEPSINLITDENTK.K
8375	545 - 562	1039.0222	2076.0299	2076.0263	1.74	0	12	0.065	1	U	K.DIFIDEPSINLITDENTK.K
8376	545 - 562	1039.0223	2076.0301	2076.0263	1.86	0	96	2.7e-010	1	U	K.DIFIDEPSINLITDENTK.K
8377	545 - 562	1039.0231	2076.0316	2076.0263	2.57	0	73	5e-008	1	U	K.DIFIDEPSINLITDENTK.K
9010	545 - 563	735.7136	2204.1190	2204.1212	-0.99	1	27	0.0019	1	U	K.DIFIDEPSINLITDENTKK.E
9011	545 - 563	735.7145	2204.1218	2204.1212	0.26	1	3	0.46	1	U	K.DIFIDEPSINLITDENTKK.E
9012	545 - 563	735.7147	2204.1222	2204.1212	0.43	1	9	0.14	1	U	K.DIFIDEPSINLITDENTKK.E
9013	545 - 563	1103.0692	2204.1239	2204.1212	1.21	1	89	1.3e-009	1	U	K.DIFIDEPSINLITDENTKK.E
9014	545 - 563	735.7158	2204.1254	2204.1212	1.92	1	4	0.41	1	U	K.DIFIDEPSINLITDENTKK.E
9015	545 - 563	735.7159	2204.1258	2204.1212	2.09	1	18	0.017	1	U	K.DIFIDEPSINLITDENTKK.E
9016	545 - 563	735.7177	2204.1313	2204.1212	4.58	1	9	0.14	1	U	K.DIFIDEPSINLITDENTKK.E

Query	Start - End	Observed	Mr(expt)	Mr(calc)	ppm	M	Score	Expect	Rank	U	Peptide
360	563 - 567	337.6951	673.3756	673.3759	-0.40	1	14	0.043	1	U	K.KEIER.Q
114	564 - 567	273.6477	545.2809	545.2809	-0.075	0	7	0.22	1	U	K.EIER.Q
115	564 - 567	273.6477	545.2809	545.2809	-0.075	0	6	0.25	1	U	K.EIER.Q
116	564 - 567	273.6477	545.2809	545.2809	0.039	0	5	0.32	1	U	K.EIER.Q
117	564 - 567	273.6477	545.2809	545.2809	0.039	0	4	0.36	1	U	K.EIER.Q
1352	568 - 575	458.2391	914.4637	914.4644	-0.71	0	4	0.36	1	U	R.QLNQMPGK.T
1353	568 - 575	458.2392	914.4639	914.4644	-0.51	0	42	6.3e-005	1	U	R.QLNQMPGK.T
1469	568 - 575	466.2366	930.4587	930.4593	-0.62	0	29	0.0014	1	U	R.QLNQMPGK.T + Oxidation (M)
1470	568 - 575	466.2368	930.4590	930.4593	-0.29	0	37	0.00018	1	U	R.QLNQMPGK.T + Oxidation (M)
1471	568 - 575	466.2370	930.4595	930.4593	0.23	0	39	0.00014	1	U	R.QLNQMPGK.T + Oxidation (M)
1472	568 - 575	466.2370	930.4595	930.4593	0.23	0	40	0.0001	1	U	R.QLNQMPGK.T + Oxidation (M)
1473	568 - 575	466.2372	930.4599	930.4593	0.63	0	25	0.0029	1	U	R.QLNQMPGK.T + Oxidation (M)
1474	568 - 575	466.2373	930.4601	930.4593	0.83	0	41	7.5e-005	1	U	R.QLNQMPGK.T + Oxidation (M)
1475	568 - 575	466.2390	930.4635	930.4593	4.56	0	34	0.00041	1	U	R.QLNQMPGK.T + Oxidation (M)
5396	568 - 581	546.2839	1635.8298	1635.8290	0.46	1	16	0.024	1	U	R.QLNQMPGKTVYDVK.W + Oxidation (M)
474	576 - 581	362.6970	723.3794	723.3803	-1.30	0	14	0.044	1	U	K.TVYDVK.W
475	576 - 581	362.6971	723.3796	723.3803	-1.05	0	14	0.038	1	U	K.TVYDVK.W
476	576 - 581	362.6971	723.3796	723.3803	-0.96	0	19	0.012	1	U	K.TVYDVK.W
477	576 - 581	362.6973	723.3800	723.3803	-0.37	0	15	0.03	1	U	K.TVYDVK.W
478	576 - 581	362.6973	723.3800	723.3803	-0.37	0	15	0.03	1	U	K.TVYDVK.W
479	576 - 581	362.6974	723.3803	723.3803	-0.036	0	16	0.027	1	U	K.TVYDVK.W
480	576 - 581	362.6975	723.3804	723.3803	0.13	0	12	0.057	1	U	K.TVYDVK.W
481	576 - 581	362.6975	723.3805	723.3803	0.22	0	10	0.093	1	U	K.TVYDVK.W
482	576 - 581	362.6976	723.3807	723.3803	0.56	0	22	0.0058	1	U	K.TVYDVK.W
1394	588 - 595	307.5353	919.5842	919.5855	-1.40	0	25	0.0034	1	U	K.ITLHVPIK.Y
1395	588 - 595	460.7997	919.5848	919.5855	-0.79	0	19	0.013	1	U	K.ITLHVPIK.Y
1396	588 - 595	307.5356	919.5849	919.5855	-0.61	0	33	0.00047	1	U	K.ITLHVPIK.Y
1397	588 - 595	307.5357	919.5854	919.5855	-0.11	0	35	0.00031	1	U	K.ITLHVPIK.Y
1398	588 - 595	307.5357	919.5854	919.5855	-0.11	0	33	0.00055	1	U	K.ITLHVPIK.Y
1399	588 - 595	307.5358	919.5856	919.5855	0.090	0	31	0.00074	1	U	K.ITLHVPIK.Y
1400	588 - 595	307.5358	919.5857	919.5855	0.19	0	31	0.00077	1	U	K.ITLHVPIK.Y
1401	588 - 595	307.5358	919.5857	919.5855	0.19	0	33	0.00046	1	U	K.ITLHVPIK.Y
1402	588 - 595	307.5359	919.5857	919.5855	0.29	0	38	0.00016	1	U	K.ITLHVPIK.Y
1403	588 - 595	460.8002	919.5858	919.5855	0.34	0	20	0.0099	1	U	K.ITLHVPIK.Y
1404	588 - 595	460.8004	919.5863	919.5855	0.87	0	22	0.0062	1	U	K.ITLHVPIK.Y
1405	588 - 595	307.5360	919.5863	919.5855	0.89	0	19	0.012	1	U	K.ITLHVPIK.Y
1406	588 - 595	307.5360	919.5863	919.5855	0.89	0	33	0.00052	1	U	K.ITLHVPIK.Y
1407	588 - 595	460.8005	919.5864	919.5855	1.00	0	28	0.0015	1	U	K.ITLHVPIK.Y
1408	588 - 595	307.5361	919.5865	919.5855	1.09	0	23	0.0052	1	U	K.ITLHVPIK.Y
1409	588 - 595	460.8013	919.5880	919.5855	2.73	0	19	0.011	1	U	K.ITLHVPIK.Y
12754	596 - 619	988.7520	2963.2342	2963.2450	-3.63	0	40	0.00011	1	U	K.YYDFETSENLWYYTYQESGGYTGK.K
12755	596 - 619	1482.6255	2963.2364	2963.2450	-2.89	0	3	0.49	1	U	K.YYDFETSENLWYYTYQESGGYTGK.K
12756	596 - 619	988.7533	2963.2381	2963.2450	-2.34	0	18	0.014	1	U	K.YYDFETSENLWYYTYQESGGYTGK.K
12757	596 - 619	1482.6268	2963.2391	2963.2450	-1.98	0	15	0.033	1	U	K.YYDFETSENLWYYTYQESGGYTGK.K
12758	596 - 619	988.7537	2963.2392	2963.2450	-1.96	0	57	2.2e-006	1	U	K.YYDFETSENLWYYTYQESGGYTGK.K
12759	596 - 619	988.7538	2963.2395	2963.2450	-1.84	0	50	1e-005	1	U	K.YYDFETSENLWYYTYQESGGYTGK.K
12760	596 - 619	988.7539	2963.2399	2963.2450	-1.72	0	43	5.3e-005	1	U	K.YYDFETSENLWYYTYQESGGYTGK.K
12761	596 - 619	1482.6277	2963.2408	2963.2450	-1.40	0	8	0.15	1	U	K.YYDFETSENLWYYTYQESGGYTGK.K
12762	596 - 619	988.7545	2963.2415	2963.2450	-1.16	0	50	1e-005	1	U	K.YYDFETSENLWYYTYQESGGYTGK.K
12763	596 - 619	1482.6282	2963.2418	2963.2450	-1.07	0	12	0.066	1	U	K.YYDFETSENLWYYTYQESGGYTGK.K
12764	596 - 619	988.7546	2963.2419	2963.2450	-1.04	0	28	0.0017	1	U	K.YYDFETSENLWYYTYQESGGYTGK.K
12765	596 - 619	988.7546	2963.2419	2963.2450	-1.04	0	57	1.9e-006	1	U	K.YYDFETSENLWYYTYQESGGYTGK.K
12766	596 - 619	1482.6283	2963.2420	2963.2450	-0.99	0	30	0.00098	1	U	K.YYDFETSENLWYYTYQESGGYTGK.K
12767	596 - 619	988.7547	2963.2423	2963.2450	-0.91	0	64	3.7e-007	1	U	K.YYDFETSENLWYYTYQESGGYTGK.K
12768	596 - 619	1482.6288	2963.2430	2963.2450	-0.66	0	13	0.05	1	U	K.YYDFETSENLWYYTYQESGGYTGK.K
12769	596 - 619	1482.6289	2963.2433	2963.2450	-0.58	0	9	0.13	1	U	K.YYDFETSENLWYYTYQESGGYTGK.K
12770	596 - 619	1482.6290	2963.2435	2963.2450	-0.50	0	19	0.012	1	U	K.YYDFETSENLWYYTYQESGGYTGK.K
12771	596 - 619	988.7551	2963.2436	2963.2450	-0.48	0	60	1e-006	1	U	K.YYDFETSENLWYYTYQESGGYTGK.K
12772	596 - 619	988.7552	2963.2439	2963.2450	-0.36	0	62	5.6e-007	1	U	K.YYDFETSENLWYYTYQESGGYTGK.K
12773	596 - 619	988.7553	2963.2441	2963.2450	-0.30	0	32	0.0006	1	U	K.YYDFETSENLWYYTYQESGGYTGK.K
12774	596 - 619	1482.6294	2963.2442	2963.2450	-0.25	0	9	0.12	1	U	K.YYDFETSENLWYYTYQESGGYTGK.K
12775	596 - 619	988.7554	2963.2443	2963.2450	-0.23	0	47	2e-005	1	U	K.YYDFETSENLWYYTYQESGGYTGK.K
12776	596 - 619	1482.6296	2963.2447	2963.2450	-0.086	0	19	0.012	1	U	K.YYDFETSENLWYYTYQESGGYTGK.K
12777	596 - 619	988.7557	2963.2454	2963.2450	0.14	0	72	6e-008	1	U	K.YYDFETSENLWYYTYQESGGYTGK.K
12778	596 - 619	988.7557	2963.2454	2963.2450	0.14	0	85	3.2e-009	1	U	K.YYDFETSENLWYYTYQESGGYTGK.K
12780	596 - 619	988.7559	2963.2459	2963.2450	0.32	0	67	2e-007	1	U	K.YYDFETSENLWYYTYQESGGYTGK.K
12781	596 - 619	1482.6302	2963.2459	2963.2450	0.33	0	0	0.95	1	U	K.YYDFETSENLWYYTYQESGGYTGK.K
12782	596 - 619	1482.6302	2963.2459	2963.2450	0.33	0	1	0.74	1	U	K.YYDFETSENLWYYTYQESGGYTGK.K
12783	596 - 619	988.7560	2963.2463	2963.2450	0.44	0	37	0.0002	1	U	K.YYDFETSENLWYYTYQESGGYTGK.K
12784	596 - 619	1482.6305	2963.2464	2963.2450	0.49	0	4	0.39	1	U	K.YYDFETSENLWYYTYQESGGYTGK.K
12785	596 - 619	988.7562	2963.2467	2963.2450	0.57	0	60	9.4e-007	1	U	K.YYDFETSENLWYYTYQESGGYTGK.K
12786	596 - 619	1482.6306	2963.2467	2963.2450	0.57	0	15	0.029	1	U	K.YYDFETSENLWYYTYQESGGYTGK.K
12787	596 - 619	988.7562	2963.2469	2963.2450	0.63	0	58	1.8e-006	1	U	K.YYDFETSENLWYYTYQESGGYTGK.K
12788	596 - 619	988.7563	2963.2470	2963.2450	0.69	0	56	2.6e-006	1	U	K.YYDFETSENLWYYTYQESGGYTGK.K
12789	596 - 619	988.7563	2963.2470	2963.2450	0.69	0	64	4.1e-007	1	U	K.YYDFETSENLWYYTYQESGGYTGK.K
12790	596 - 619	988.7563	2963.2470	2963.2450	0.69	0	38	0.00014	1	U	K.YYDFETSENLWYYTYQESGGYTGK.K
12792	596 - 619	988.7565	2963.2476	2963.2450	0.88	0	37	0.00022	1	U	K.YYDFETSENLWYYTYQESGGYTGK.K
12793	596 - 619	1482.6311	2963.2477	2963.2450	0.90	0	45	3.1e-005	1	U	K.YYDFETSENLWYYTYQESGGYTGK.K
12794	596 - 619	988.7565	2963.2478	2963.2450	0.94	0	38	0.00014	1	U	K.YYDFETSENLWYYTYQESGGYTGK.K
12795	596 - 619	1482.6312	2963.2479	2963.2450	0.99	0	0	0.9	1	U	K.YYDFETSENLWYYTYQESGGYTGK.K
12796	596 - 619	988.7568	2963.2487	2963.2450	1.25	0	69	1.3e-007	1	U	K.YYDFETSENLWYYTYQESGGYTGK.K
12797	596 - 619	988.7571	2963.2494	2963.2450	1.50	0	45	3.4e-005	1	U	K.YYDFETSENLWYYTYQESGGYTGK.K
12798	596 - 619	988.7571	2963.2496	2963.2450	1.56	0	39	0.00011	1	U	K.YYDFETSENLWYYTYQESGGYTGK.K
12800	596 - 619	988.7574	2963.2503	2963.2450	1.80	0	58	1.6e-006	1	U	K.YYDFETSENLWYYTYQESGGYTGK.K
12801	596 - 619	1482.6326	2963.2506	2963.2450	1.89	0	61	7.3e-007	1	U	K.YYDFETSENLWYYTYQESGGYTGK.K
12802	596 - 619	988.7577	2963.2512	2963.2450	2.11	0	28	0.0016	1	U	K.YYDFETSENLWYYTYQESGGYTGK.K
12803	596 - 619	1482.6331	2963.2516	2963.2450	2.22	0	10	0.1	1	U	K.YYDFETSENLWYYTYQESGGYTGK.K
12804	596 - 619	1482.6331	2963.2516	2963.2450	2.22	0	2	0.67	1	U	K.YYDFETSENLWYYTYQESGGYTGK.K

Query	Start - End	Observed	Mr(expt)	Mr(calc)	ppm	M	Score	Expect	Rank	U	Peptide
12805	596 - 619	1482.6333	2963.2521	2963.2450	2.39	0	67	2.1e-007	1	U	K.YYDFETSENLWYYTYQESGGYTGK.K
12806	596 - 619	1482.6335	2963.2525	2963.2450	2.55	0	8	0.16	1	U	K.YYDFETSENLWYYTYQESGGYTGK.K
12807	596 - 619	988.7582	2963.2527	2963.2450	2.61	0	36	0.00027	1	U	K.YYDFETSENLWYYTYQESGGYTGK.K
12808	596 - 619	741.8206	2963.2531	2963.2450	2.75	0	56	2.7e-006	1	U	K.YYDFETSENLWYYTYQESGGYTGK.K
12810	596 - 619	988.7590	2963.2551	2963.2450	3.41	0	48	1.5e-005	1	U	K.YYDFETSENLWYYTYQESGGYTGK.K
12811	596 - 619	1482.6355	2963.2564	2963.2450	3.87	0	16	0.023	1	U	K.YYDFETSENLWYYTYQESGGYTGK.K
13263	596 - 620	1031.4535	3091.3386	3091.3399	-0.42	1	33	0.00051	1	U	K.YYDFETSENLWYYTYQESGGYTGKK.R
13264	596 - 620	1031.4543	3091.3412	3091.3399	0.41	1	68	1.5e-007	1	U	K.YYDFETSENLWYYTYQESGGYTGKK.R
13265	596 - 620	1031.4562	3091.3467	3091.3399	2.19	1	18	0.016	1	U	K.YYDFETSENLWYYTYQESGGYTGKK.R
11134	624 - 647	1297.6353	2593.2560	2593.2595	-1.36	0	40	0.00011	1	U	R.IGTDGHGTAMSNPQLKPYTSYTVR.A
11135	624 - 647	649.3219	2593.2585	2593.2595	-0.39	0	27	0.0018	1	U	R.IGTDGHGTAMSNPQLKPYTSYTVR.A
11136	624 - 647	865.4268	2593.2586	2593.2595	-0.33	0	40	0.00011	1	U	R.IGTDGHGTAMSNPQLKPYTSYTVR.A
11137	624 - 647	649.3221	2593.2592	2593.2595	-0.10	0	39	0.00012	1	U	R.IGTDGHGTAMSNPQLKPYTSYTVR.A
11138	624 - 647	649.3221	2593.2595	2593.2595	-0.0073	0	16	0.027	1	U	R.IGTDGHGTAMSNPQLKPYTSYTVR.A
11139	624 - 647	865.4274	2593.2603	2593.2595	0.30	0	44	3.6e-005	1	U	R.IGTDGHGTAMSNPQLKPYTSYTVR.A
11220	624 - 647	1305.6298	2609.2450	2609.2544	-3.62	0	57	2.1e-006	1	U	R.IGTDGHGTAMSNPQLKPYTSYTVR.A + Oxidation (M)
11221	624 - 647	653.3199	2609.2507	2609.2544	-1.43	0	38	0.00015	1	U	R.IGTDGHGTAMSNPQLKPYTSYTVR.A + Oxidation (M)
11222	624 - 647	870.7582	2609.2529	2609.2544	-0.58	0	22	0.0065	1	U	R.IGTDGHGTAMSNPQLKPYTSYTVR.A + Oxidation (M)
11224	624 - 647	653.3206	2609.2534	2609.2544	-0.40	0	27	0.0019	1	U	R.IGTDGHGTAMSNPQLKPYTSYTVR.A + Oxidation (M)
11226	624 - 647	1305.6344	2609.2542	2609.2544	-0.063	0	70	1.1e-007	1	U	R.IGTDGHGTAMSNPQLKPYTSYTVR.A + Oxidation (M)
11227	624 - 647	522.8582	2609.2544	2609.2544	-0.0077	0	33	0.00052	1	U	R.IGTDGHGTAMSNPQLKPYTSYTVR.A + Oxidation (M)
11228	624 - 647	870.7589	2609.2547	2609.2544	0.12	0	35	0.00033	1	U	R.IGTDGHGTAMSNPQLKPYTSYTVR.A + Oxidation (M)
11229	624 - 647	653.3211	2609.2553	2609.2544	0.35	0	27	0.002	1	U	R.IGTDGHGTAMSNPQLKPYTSYTVR.A + Oxidation (M)
11230	624 - 647	870.7591	2609.2555	2609.2544	0.40	0	24	0.0038	1	U	R.IGTDGHGTAMSNPQLKPYTSYTVR.A + Oxidation (M)
11231	624 - 647	653.3212	2609.2556	2609.2544	0.44	0	42	5.6e-005	1	U	R.IGTDGHGTAMSNPQLKPYTSYTVR.A + Oxidation (M)
11232	624 - 647	870.7592	2609.2556	2609.2544	0.47	0	6	0.23	1	U	R.IGTDGHGTAMSNPQLKPYTSYTVR.A + Oxidation (M)
11233	624 - 647	653.3212	2609.2558	2609.2544	0.54	0	12	0.06	1	U	R.IGTDGHGTAMSNPQLKPYTSYTVR.A + Oxidation (M)
11234	624 - 647	653.3213	2609.2561	2609.2544	0.63	0	5	0.35	1	U	R.IGTDGHGTAMSNPQLKPYTSYTVR.A + Oxidation (M)
11236	624 - 647	653.3214	2609.2563	2609.2544	0.72	0	22	0.0059	1	U	R.IGTDGHGTAMSNPQLKPYTSYTVR.A + Oxidation (M)
11237	624 - 647	653.3214	2609.2563	2609.2544	0.72	0	28	0.0015	1	U	R.IGTDGHGTAMSNPQLKPYTSYTVR.A + Oxidation (M)
11238	624 - 647	653.3214	2609.2565	2609.2544	0.82	0	16	0.023	1	U	R.IGTDGHGTAMSNPQLKPYTSYTVR.A + Oxidation (M)
11239	624 - 647	870.7595	2609.2567	2609.2544	0.89	0	28	0.0017	1	U	R.IGTDGHGTAMSNPQLKPYTSYTVR.A + Oxidation (M)
11240	624 - 647	653.3215	2609.2570	2609.2544	1.00	0	4	0.42	1	U	R.IGTDGHGTAMSNPQLKPYTSYTVR.A + Oxidation (M)
11241	624 - 647	870.7598	2609.2575	2609.2544	1.17	0	28	0.0014	1	U	R.IGTDGHGTAMSNPQLKPYTSYTVR.A + Oxidation (M)
11242	624 - 647	653.3218	2609.2580	2609.2544	1.38	0	1	0.8	1	U	R.IGTDGHGTAMSNPQLKPYTSYTVR.A + Oxidation (M)
11243	624 - 647	870.7600	2609.2582	2609.2544	1.45	0	25	0.0028	1	U	R.IGTDGHGTAMSNPQLKPYTSYTVR.A + Oxidation (M)
11245	624 - 647	653.3218	2609.2582	2609.2544	1.47	0	30	0.001	1	U	R.IGTDGHGTAMSNPQLKPYTSYTVR.A + Oxidation (M)
11246	624 - 647	653.3219	2609.2585	2609.2544	1.56	0	26	0.0026	1	U	R.IGTDGHGTAMSNPQLKPYTSYTVR.A + Oxidation (M)
11247	624 - 647	653.3221	2609.2592	2609.2544	1.85	0	3	0.54	1	U	R.IGTDGHGTAMSNPQLKPYTSYTVR.A + Oxidation (M)
11248	624 - 647	653.3221	2609.2592	2609.2544	1.85	0	8	0.15	1	U	R.IGTDGHGTAMSNPQLKPYTSYTVR.A + Oxidation (M)
11250	624 - 647	653.3221	2609.2595	2609.2544	1.94	0	1	0.72	1	U	R.IGTDGHGTAMSNPQLKPYTSYTVR.A + Oxidation (M)
11251	624 - 647	653.3226	2609.2612	2609.2544	2.59	0	1	0.88	1	U	R.IGTDGHGTAMSNPQLKPYTSYTVR.A + Oxidation (M)
11252	624 - 647	870.7611	2609.2615	2609.2544	2.72	0	36	0.00025	1	U	R.IGTDGHGTAMSNPQLKPYTSYTVR.A + Oxidation (M)
56	648 - 651	254.6474	507.2803	507.2805	-0.35	0	3	0.49	1	U	R.AYVR.T
57	648 - 651	254.6475	507.2804	507.2805	-0.29	0	13	0.05	1	U	R.AYVR.T
58	648 - 651	254.6475	507.2805	507.2805	-0.11	0	16	0.026	1	U	R.AYVR.T
59	648 - 651	254.6475	507.2805	507.2805	-0.11	0	1	0.8	1	U	R.AYVR.T
60	648 - 651	254.6476	507.2806	507.2805	0.25	0	13	0.048	1	U	R.AYVR.T
10174	652 - 676	821.3716	2461.0929	2461.0994	-2.61	0	29	0.0013	1	U	R.TASTTGSNEVVFYADNSSGNGQGAK.V
10175	652 - 676	821.3726	2461.0960	2461.0994	-1.35	0	54	3.6e-006	1	U	R.TASTTGSNEVVFYADNSSGNGQGAK.V
10178	652 - 676	1231.5562	2461.0978	2461.0994	-0.65	0	78	1.7e-008	1	U	R.TASTTGSNEVVFYADNSSGNGQGAK.V
10179	652 - 676	821.3734	2461.0982	2461.0994	-0.46	0	52	6.5e-006	1	U	R.TASTTGSNEVVFYADNSSGNGQGAK.V
10180	652 - 676	821.3735	2461.0988	2461.0994	-0.23	0	20	0.01	1	U	R.TASTTGSNEVVFYADNSSGNGQGAK.V
10181	652 - 676	821.3737	2461.0991	2461.0994	-0.084	0	6	0.25	1	U	R.TASTTGSNEVVFYADNSSGNGQGAK.V
10182	652 - 676	821.3738	2461.0997	2461.0994	0.14	0	20	0.011	1	U	R.TASTTGSNEVVFYADNSSGNGQGAK.V
10183	652 - 676	1231.5579	2461.1012	2461.0994	0.74	0	59	1.2e-006	1	U	R.TASTTGSNEVVFYADNSSGNGQGAK.V
10184	652 - 676	821.3745	2461.1017	2461.0994	0.96	0	13	0.056	1	U	R.TASTTGSNEVVFYADNSSGNGQGAK.V
10185	652 - 676	821.3746	2461.1019	2461.0994	1.03	0	19	0.013	1	U	R.TASTTGSNEVVFYADNSSGNGQGAK.V
10186	652 - 676	821.3749	2461.1028	2461.0994	1.40	0	25	0.0032	1	U	R.TASTTGSNEVVFYADNSSGNGQGAK.V
10187	652 - 676	821.3750	2461.1032	2461.0994	1.55	0	29	0.0012	1	U	R.TASTTGSNEVVFYADNSSGNGQGAK.V
10188	652 - 676	821.3751	2461.1035	2461.0994	1.70	0	8	0.17	1	U	R.TASTTGSNEVVFYADNSSGNGQGAK.V
10190	652 - 676	821.3758	2461.1056	2461.0994	2.52	0	34	0.00041	1	U	R.TASTTGSNEVVFYADNSSGNGQGAK.V
10191	652 - 676	821.3772	2461.1098	2461.0994	4.23	0	18	0.015	1	U	R.TASTTGSNEVVFYADNSSGNGQGAK.V
26	681 - 685	231.1395	460.2644	460.2646	-0.25	0	15	0.029	1	U	K.VTGGK.W
670	681 - 687	388.2263	774.4381	774.4388	-0.96	1	3	0.47	1	U	K.VTGGKWK.I
7447	688 - 703	984.4598	1966.9051	1966.9101	-2.53	0	36	0.00024	1	U	K.IAEFSFNTFNNPEYFK.I
7448	688 - 703	984.4600	1966.9055	1966.9101	-2.35	0	58	1.6e-006	1	U	K.IAEFSFNTFNNPEYFK.I
7449	688 - 703	984.4607	1966.9068	1966.9101	-1.67	0	44	3.7e-005	1	U	K.IAEFSFNTFNNPEYFK.I
7450	688 - 703	984.4609	1966.9073	1966.9101	-1.42	0	62	5.6e-007	1	U	K.IAEFSFNTFNNPEYFK.I
7451	688 - 703	656.6432	1966.9077	1966.9101	-1.21	0	41	8.2e-005	1	U	K.IAEFSFNTFNNPEYFK.I
7452	688 - 703	984.4613	1966.9081	1966.9101	-1.04	0	109	1.3e-011	1	U	K.IAEFSFNTFNNPEYFK.I
7453	688 - 703	656.6434	1966.9083	1966.9101	-0.93	0	17	0.018	1	U	K.IAEFSFNTFNNPEYFK.I
7454	688 - 703	984.4615	1966.9085	1966.9101	-0.80	0	62	5.9e-007	1	U	K.IAEFSFNTFNNPEYFK.I
7455	688 - 703	656.6436	1966.9088	1966.9101	-0.65	0	8	0.14	1	U	K.IAEFSFNTFNNPEYFK.I
7456	688 - 703	656.6437	1966.9092	1966.9101	-0.46	0	30	0.001	1	U	K.IAEFSFNTFNNPEYFK.I
7457	688 - 703	984.4619	1966.9093	1966.9101	-0.42	0	61	7.1e-007	1	U	K.IAEFSFNTFNNPEYFK.I
7458	688 - 703	984.4620	1966.9094	1966.9101	-0.36	0	66	2.4e-007	1	U	K.IAEFSFNTFNNPEYFK.I
7459	688 - 703	984.4620	1966.9094	1966.9101	-0.36	0	45	3e-005	1	U	K.IAEFSFNTFNNPEYFK.I
7460	688 - 703	984.4620	1966.9095	1966.9101	-0.30	0	86	2.6e-009	1	U	K.IAEFSFNTFNNPEYFK.I
7461	688 - 703	656.6439	1966.9098	1966.9101	-0.18	0	7	0.18	1	U	K.IAEFSFNTFNNPEYFK.I
7462	688 - 703	656.6439	1966.9099	1966.9101	-0.089	0	35	0.00032	1	U	K.IAEFSFNTFNNPEYFK.I
7463	688 - 703	656.6440	1966.9101	1966.9101	0.0041	0	31	0.00086	1	U	K.IAEFSFNTFNNPEYFK.I
7464	688 - 703	984.4623	1966.9101	1966.9101	0.010	0	86	2.7e-009	1	U	K.IAEFSFNTFNNPEYFK.I
7465	688 - 703	656.6440	1966.9103	1966.9101	0.097	0	21	0.0079	1	U	K.IAEFSFNTFNNPEYFK.I
7466	688 - 703	984.4625	1966.9104	1966.9101	0.13	0	35	0.0003	1	U	K.IAEFSFNTFNNPEYFK.I
7467	688 - 703	656.6441	1966.9105	1966.9101	0.19	0	27	0.0022	1	U	K.IAEFSFNTFNNPEYFK.I
7468	688 - 703	656.6441	1966.9105	1966.9101	0.19	0	54	3.9e-006	1	U	K.IAEFSFNTFNNPEYFK.I
7469	688 - 703	984.4625	1966.9105	1966.9101	0.20	0	47	1.9e-005	1	U	K.IAEFSFNTFNNPEYFK.I

Query	Start - End	Observed	Mr(expt)	Mr(calc)	ppm	M	Score	Expect	Rank	U	Peptide
7470	688 - 703	984.4626	1966.9106	1966.9101	0.26	0	39	0.00011	1	U	K.IAEFSFNTFNNPEYFK.I
7471	688 - 703	984.4626	1966.9106	1966.9101	0.26	0	73	5.2e-008	1	U	K.IAEFSFNTFNNPEYFK.I
7472	688 - 703	984.4626	1966.9106	1966.9101	0.26	0	63	4.8e-007	1	U	K.IAEFSFNTFNNPEYFK.I
7473	688 - 703	656.6442	1966.9107	1966.9101	0.28	0	49	1.3e-005	1	U	K.IAEFSFNTFNNPEYFK.I
7474	688 - 703	984.4626	1966.9107	1966.9101	0.32	0	67	2.1e-007	1	U	K.IAEFSFNTFNNPEYFK.I
7475	688 - 703	656.6442	1966.9109	1966.9101	0.38	0	30	0.00094	1	U	K.IAEFSFNTFNNPEYFK.I
7476	688 - 703	984.4628	1966.9110	1966.9101	0.45	0	63	5.2e-007	1	U	K.IAEFSFNTFNNPEYFK.I
7477	688 - 703	984.4628	1966.9110	1966.9101	0.45	0	76	2.6e-008	1	U	K.IAEFSFNTFNNPEYFK.I
7478	688 - 703	984.4629	1966.9112	1966.9101	0.57	0	61	7.5e-007	1	U	K.IAEFSFNTFNNPEYFK.I
7479	688 - 703	984.4630	1966.9114	1966.9101	0.63	0	86	2.8e-009	1	U	K.IAEFSFNTFNNPEYFK.I
7480	688 - 703	656.6444	1966.9114	1966.9101	0.66	0	14	0.036	1	U	K.IAEFSFNTFNNPEYFK.I
7481	688 - 703	984.4630	1966.9115	1966.9101	0.69	0	87	2.2e-009	1	U	K.IAEFSFNTFNNPEYFK.I
7482	688 - 703	656.6445	1966.9116	1966.9101	0.75	0	36	0.00026	1	U	K.IAEFSFNTFNNPEYFK.I
7483	688 - 703	656.6445	1966.9116	1966.9101	0.75	0	44	3.9e-005	1	U	K.IAEFSFNTFNNPEYFK.I
7484	688 - 703	984.4631	1966.9116	1966.9101	0.76	0	73	5.1e-008	1	U	K.IAEFSFNTFNNPEYFK.I
7485	688 - 703	984.4631	1966.9116	1966.9101	0.76	0	57	2.1e-006	1	U	K.IAEFSFNTFNNPEYFK.I
7486	688 - 703	656.6445	1966.9118	1966.9101	0.84	0	20	0.0094	1	U	K.IAEFSFNTFNNPEYFK.I
7487	688 - 703	984.4632	1966.9118	1966.9101	0.88	0	51	7.7e-006	1	U	K.IAEFSFNTFNNPEYFK.I
7488	688 - 703	656.6446	1966.9119	1966.9101	0.93	0	6	0.23	1	U	K.IAEFSFNTFNNPEYFK.I
7489	688 - 703	656.6446	1966.9119	1966.9101	0.93	0	20	0.009	1	U	K.IAEFSFNTFNNPEYFK.I
7490	688 - 703	984.4633	1966.9120	1966.9101	0.94	0	44	4.2e-005	1	U	K.IAEFSFNTFNNPEYFK.I
7491	688 - 703	984.4633	1966.9121	1966.9101	1.00	0	52	5.6e-006	1	U	K.IAEFSFNTFNNPEYFK.I
7492	688 - 703	984.4633	1966.9121	1966.9101	1.00	0	76	2.7e-008	1	U	K.IAEFSFNTFNNPEYFK.I
7493	688 - 703	984.4633	1966.9121	1966.9101	1.00	0	57	2e-006	1	U	K.IAEFSFNTFNNPEYFK.I
7494	688 - 703	984.4634	1966.9122	1966.9101	1.07	0	65	3.4e-007	1	U	K.IAEFSFNTFNNPEYFK.I
7495	688 - 703	984.4634	1966.9122	1966.9101	1.07	0	55	3.1e-006	1	U	K.IAEFSFNTFNNPEYFK.I
7496	688 - 703	984.4634	1966.9123	1966.9101	1.13	0	71	7.8e-008	1	U	K.IAEFSFNTFNNPEYFK.I
7497	688 - 703	656.6448	1966.9127	1966.9101	1.31	0	10	0.11	1	U	K.IAEFSFNTFNNPEYFK.I
7498	688 - 703	656.6448	1966.9127	1966.9101	1.31	0	33	0.00051	1	U	K.IAEFSFNTFNNPEYFK.I
7499	688 - 703	984.4636	1966.9127	1966.9101	1.31	0	46	2.4e-005	1	U	K.IAEFSFNTFNNPEYFK.I
7500	688 - 703	656.6449	1966.9129	1966.9101	1.40	0	35	0.00028	1	U	K.IAEFSFNTFNNPEYFK.I
7501	688 - 703	656.6450	1966.9130	1966.9101	1.49	0	12	0.057	1	U	K.IAEFSFNTFNNPEYFK.I
7502	688 - 703	656.6451	1966.9134	1966.9101	1.68	0	32	0.00064	1	U	K.IAEFSFNTFNNPEYFK.I
7503	688 - 703	984.4640	1966.9134	1966.9101	1.69	0	57	2.2e-006	1	U	K.IAEFSFNTFNNPEYFK.I
7504	688 - 703	984.4641	1966.9135	1966.9101	1.75	0	60	9.9e-007	1	U	K.IAEFSFNTFNNPEYFK.I
7506	688 - 703	984.4641	1966.9137	1966.9101	1.81	0	50	1.1e-005	1	U	K.IAEFSFNTFNNPEYFK.I
7507	688 - 703	984.4641	1966.9137	1966.9101	1.81	0	60	9e-007	1	U	K.IAEFSFNTFNNPEYFK.I
7508	688 - 703	656.6452	1966.9138	1966.9101	1.87	0	11	0.076	1	U	K.IAEFSFNTFNNPEYFK.I
7509	688 - 703	656.6452	1966.9138	1966.9101	1.87	0	32	0.00064	1	U	K.IAEFSFNTFNNPEYFK.I
7510	688 - 703	984.4642	1966.9139	1966.9101	1.93	0	50	1.1e-005	1	U	K.IAEFSFNTFNNPEYFK.I
7511	688 - 703	984.4644	1966.9142	1966.9101	2.06	0	58	1.5e-006	1	U	K.IAEFSFNTFNNPEYFK.I
7512	688 - 703	984.4645	1966.9144	1966.9101	2.18	0	21	0.0077	1	U	K.IAEFSFNTFNNPEYFK.I
7513	688 - 703	656.6455	1966.9147	1966.9101	2.33	0	29	0.0011	1	U	K.IAEFSFNTFNNPEYFK.I
7514	688 - 703	984.4647	1966.9149	1966.9101	2.43	0	78	1.7e-008	1	U	K.IAEFSFNTFNNPEYFK.I
7515	688 - 703	984.4667	1966.9189	1966.9101	4.48	0	28	0.0016	1	U	K.IAEFSFNTFNNPEYFK.I
101	704 - 708	272.1966	542.3787	542.3792	-0.86	0	17	0.022	1	U	K.IIGLK.N
102	704 - 708	272.1967	542.3789	542.3792	-0.41	0	12	0.062	1	U	K.IIGLK.N
103	704 - 708	272.1967	542.3789	542.3792	-0.41	0	20	0.0092	1	U	K.IIGLK.N
104	704 - 708	272.1968	542.3791	542.3792	-0.19	0	21	0.0089	1	U	K.IIGLK.N
105	704 - 708	272.1968	542.3791	542.3792	-0.19	0	22	0.006	1	U	K.IIGLK.N
106	704 - 708	272.1970	542.3794	542.3792	0.37	0	16	0.025	1	U	K.IIGLK.N
107	704 - 708	272.1970	542.3794	542.3792	0.49	0	15	0.03	1	U	K.IIGLK.N
108	704 - 708	272.1971	542.3796	542.3792	0.71	0	21	0.0088	1	U	K.IIGLK.N
109	704 - 708	272.1971	542.3797	542.3792	0.94	0	16	0.026	1	U	K.IIGLK.N
110	704 - 708	272.1973	542.3800	542.3792	1.61	0	24	0.0041	1	U	K.IIGLK.N
8244	709 - 726	691.3297	2070.9671	2070.9759	-4.24	0	33	0.00048	1	U	K.NNGNANLHFDDVSVIEWK.T
8245	709 - 726	691.3311	2070.9715	2070.9759	-2.12	0	28	0.0016	1	U	K.NNGNANLHFDDVSVIEWK.T
8246	709 - 726	691.3314	2070.9724	2070.9759	-1.68	0	24	0.0035	1	U	K.NNGNANLHFDDVSVIEWK.T
8247	709 - 726	1036.4940	2070.9735	2070.9759	-1.17	0	53	5.4e-006	1	U	K.NNGNANLHFDDVSVIEWK.T
8248	709 - 726	691.3319	2070.9739	2070.9759	-0.97	0	40	0.0001	1	U	K.NNGNANLHFDDVSVIEWK.T
8249	709 - 726	691.3322	2070.9746	2070.9759	-0.62	0	11	0.08	1	U	K.NNGNANLHFDDVSVIEWK.T
8250	709 - 726	691.3322	2070.9748	2070.9759	-0.53	0	27	0.0022	1	U	K.NNGNANLHFDDVSVIEWK.T
8251	709 - 726	691.3322	2070.9748	2070.9759	-0.53	0	38	0.00014	1	U	K.NNGNANLHFDDVSVIEWK.T
8252	709 - 726	691.3323	2070.9750	2070.9759	-0.44	0	30	0.001	1	U	K.NNGNANLHFDDVSVIEWK.T
8253	709 - 726	691.3323	2070.9752	2070.9759	-0.35	0	17	0.019	1	U	K.NNGNANLHFDDVSVIEWK.T
8254	709 - 726	691.3326	2070.9759	2070.9759	0.0043	0	44	3.8e-005	1	U	K.NNGNANLHFDDVSVIEWK.T
8255	709 - 726	691.3326	2070.9759	2070.9759	0.0043	0	9	0.12	1	U	K.NNGNANLHFDDVSVIEWK.T
8256	709 - 726	691.3326	2070.9761	2070.9759	0.093	0	29	0.0014	1	U	K.NNGNANLHFDDVSVIEWK.T
8257	709 - 726	691.3328	2070.9765	2070.9759	0.27	0	9	0.13	1	U	K.NNGNANLHFDDVSVIEWK.T
8258	709 - 726	691.3328	2070.9765	2070.9759	0.27	0	43	5.3e-005	1	U	K.NNGNANLHFDDVSVIEWK.T
8259	709 - 726	691.3328	2070.9765	2070.9759	0.27	0	30	0.00089	1	U	K.NNGNANLHFDDVSVIEWK.T
8260	709 - 726	691.3328	2070.9766	2070.9759	0.36	0	38	0.00016	1	U	K.NNGNANLHFDDVSVIEWK.T
8261	709 - 726	691.3329	2070.9770	2070.9759	0.53	0	35	0.00031	1	U	K.NNGNANLHFDDVSVIEWK.T
8262	709 - 726	691.3330	2070.9772	2070.9759	0.62	0	29	0.0012	1	U	K.NNGNANLHFDDVSVIEWK.T
8263	709 - 726	691.3330	2070.9772	2070.9759	0.62	0	37	0.00022	1	U	K.NNGNANLHFDDVSVIEWK.T
8264	709 - 726	691.3330	2070.9772	2070.9759	0.62	0	35	0.00028	1	U	K.NNGNANLHFDDVSVIEWK.T
8265	709 - 726	691.3331	2070.9774	2070.9759	0.71	0	27	0.0018	1	U	K.NNGNANLHFDDVSVIEWK.T
8266	709 - 726	691.3331	2070.9774	2070.9759	0.71	0	46	2.3e-005	1	U	K.NNGNANLHFDDVSVIEWK.T
8267	709 - 726	691.3331	2070.9774	2070.9759	0.71	0	35	0.00031	1	U	K.NNGNANLHFDDVSVIEWK.T
8268	709 - 726	691.3331	2070.9776	2070.9759	0.80	0	19	0.011	1	U	K.NNGNANLHFDDVSVIEWK.T
8269	709 - 726	691.3332	2070.9777	2070.9759	0.89	0	35	0.00035	1	U	K.NNGNANLHFDDVSVIEWK.T
8270	709 - 726	691.3334	2070.9783	2070.9759	1.15	0	41	7.3e-005	1	U	K.NNGNANLHFDDVSVIEWK.T
8271	709 - 726	691.3334	2070.9783	2070.9759	1.15	0	31	0.00081	1	U	K.NNGNANLHFDDVSVIEWK.T
8272	709 - 726	691.3334	2070.9783	2070.9759	1.15	0	41	8e-005	1	U	K.NNGNANLHFDDVSVIEWK.T
8273	709 - 726	691.3335	2070.9787	2070.9759	1.33	0	34	0.00044	1	U	K.NNGNANLHFDDVSVIEWK.T
8274	709 - 726	691.3336	2070.9788	2070.9759	1.42	0	30	0.00091	1	U	K.NNGNANLHFDDVSVIEWK.T
8275	709 - 726	691.3336	2070.9790	2070.9759	1.51	0	47	2e-005	1	U	K.NNGNANLHFDDVSVIEWK.T
8276	709 - 726	691.3339	2070.9799	2070.9759	1.95	0	39	0.00013	1	U	K.NNGNANLHFDDVSVIEWK.T

Query	Start - End	Observed	Mr(expt)	Mr(calc)	ppm	M	Score	Expect	Rank	U	Peptide
8277	709 - 726	1036.4973	2070.9801	2070.9759	2.01	0	2	0.58	1	U	K.NNGNANLHFDDVSVIEWK.T
8278	709 - 726	691.3342	2070.9809	2070.9759	2.39	0	38	0.00017	1	U	K.NNGNANLHFDDVSVIEWK.T
8279	709 - 726	691.3343	2070.9810	2070.9759	2.48	0	29	0.0012	1	U	K.NNGNANLHFDDVSVIEWK.T
8280	709 - 726	691.3344	2070.9812	2070.9759	2.57	0	17	0.021	1	U	K.NNGNANLHFDDVSVIEWK.T
8281	709 - 726	691.3345	2070.9816	2070.9759	2.75	0	25	0.0032	1	U	K.NNGNANLHFDDVSVIEWK.T
8282	709 - 726	691.3346	2070.9820	2070.9759	2.92	0	27	0.0018	1	U	K.NNGNANLHFDDVSVIEWK.T
8283	709 - 726	1036.4989	2070.9833	2070.9759	3.55	0	57	1.8e-006	1	U	K.NNGNANLHFDDVSVIEWK.T
8284	709 - 726	1036.4989	2070.9833	2070.9759	3.55	0	86	2.4e-009	1	U	K.NNGNANLHFDDVSVIEWK.T
1001	727 - 733	423.7194	845.4242	845.4243	-0.12	0	34	0.00042	1	U	K.TNENLQK.K
1002	727 - 733	423.7194	845.4243	845.4243	0.022	0	45	3.2e-005	1	U	K.TNENLQK.K
1003	727 - 733	423.7195	845.4244	845.4243	0.096	0	46	2.4e-005	1	U	K.TNENLQK.K
1778	727 - 734	487.7668	973.5190	973.5192	-0.21	1	12	0.067	1	U	K.TNENLQKK.H
1780	727 - 734	487.7671	973.5196	973.5192	0.35	1	10	0.1	1	U	K.TNENLQKK.H
1781	727 - 734	487.7673	973.5200	973.5192	0.79	1	11	0.076	1	U	K.TNENLQKK.H
778	734 - 739	401.2345	800.4544	800.4545	-0.089	1	21	0.0078	1	U	K.KHIFEK.W
779	734 - 739	401.2345	800.4544	800.4545	-0.014	1	21	0.0085	1	U	K.KHIFEK.W
780	734 - 739	401.2347	800.4549	800.4545	0.52	1	19	0.012	1	U	K.KHIFEK.W
346	735 - 739	337.1869	672.3593	672.3595	-0.31	0	2	0.6	1	U	K.HIFEK.W
347	735 - 739	337.1870	672.3594	672.3595	-0.13	0	17	0.021	1	U	K.HIFEK.W
348	735 - 739	337.1871	672.3596	672.3595	0.14	0	22	0.0064	1	U	K.HIFEK.W
349	735 - 739	337.1872	672.3598	672.3595	0.41	0	8	0.16	1	U	K.HIFEK.W
351	735 - 739	337.1873	672.3600	672.3595	0.78	0	17	0.019	1	U	K.HIFEK.W
352	735 - 739	337.1877	672.3609	672.3595	2.05	0	10	0.094	1	U	K.HIFEK.W
6923	740 - 756	959.4394	1916.8642	1916.8727	-4.42	0	103	4.9e-011	1	U	K.WSFGSNDEMVIGATFTR.V
6924	740 - 756	959.4420	1916.8694	1916.8727	-1.75	0	54	3.7e-006	1	U	K.WSFGSNDEMVIGATFTR.V
6925	740 - 756	959.4421	1916.8697	1916.8727	-1.56	0	45	3.2e-005	1	U	K.WSFGSNDEMVIGATFTR.V
6926	740 - 756	639.9642	1916.8709	1916.8727	-0.96	0	44	3.8e-005	1	U	K.WSFGSNDEMVIGATFTR.V
6927	740 - 756	959.4427	1916.8709	1916.8727	-0.92	0	31	0.00078	1	U	K.WSFGSNDEMVIGATFTR.V
6928	740 - 756	639.9643	1916.8711	1916.8727	-0.86	0	22	0.0067	1	U	K.WSFGSNDEMVIGATFTR.V
6929	740 - 756	639.9643	1916.8711	1916.8727	-0.86	0	33	0.00047	1	U	K.WSFGSNDEMVIGATFTR.V
6930	740 - 756	959.4431	1916.8716	1916.8727	-0.60	0	66	2.4e-007	1	U	K.WSFGSNDEMVIGATFTR.V
6931	740 - 756	959.4433	1916.8720	1916.8727	-0.35	0	64	3.6e-007	1	U	K.WSFGSNDEMVIGATFTR.V
6932	740 - 756	959.4433	1916.8720	1916.8727	-0.35	0	51	8.3e-006	1	U	K.WSFGSNDEMVIGATFTR.V
6933	740 - 756	959.4435	1916.8725	1916.8727	-0.093	0	35	0.00032	1	U	K.WSFGSNDEMVIGATFTR.V
6934	740 - 756	959.4436	1916.8727	1916.8727	-0.029	0	62	6e-007	1	U	K.WSFGSNDEMVIGATFTR.V
6935	740 - 756	959.4436	1916.8727	1916.8727	-0.029	0	88	1.7e-009	1	U	K.WSFGSNDEMVIGATFTR.V
6936	740 - 756	959.4436	1916.8727	1916.8727	-0.029	0	77	2e-008	1	U	K.WSFGSNDEMVIGATFTR.V
6937	740 - 756	959.4437	1916.8729	1916.8727	0.099	0	40	8.9e-005	1	U	K.WSFGSNDEMVIGATFTR.V
6938	740 - 756	959.4437	1916.8729	1916.8727	0.099	0	62	6.7e-007	1	U	K.WSFGSNDEMVIGATFTR.V
6939	740 - 756	959.4437	1916.8729	1916.8727	0.099	0	103	4.7e-011	1	U	K.WSFGSNDEMVIGATFTR.V
6940	740 - 756	959.4437	1916.8729	1916.8727	0.099	0	79	1.4e-008	1	U	K.WSFGSNDEMVIGATFTR.V
6941	740 - 756	959.4437	1916.8729	1916.8727	0.099	0	62	6.5e-007	1	U	K.WSFGSNDEMVIGATFTR.V
6942	740 - 756	959.4438	1916.8731	1916.8727	0.23	0	18	0.017	1	U	K.WSFGSNDEMVIGATFTR.V
6943	740 - 756	959.4438	1916.8731	1916.8727	0.23	0	103	5.2e-011	1	U	K.WSFGSNDEMVIGATFTR.V
6944	740 - 756	959.4438	1916.8731	1916.8727	0.23	0	55	3.2e-006	1	U	K.WSFGSNDEMVIGATFTR.V
6945	740 - 756	639.9650	1916.8733	1916.8727	0.28	0	5	0.31	1	U	K.WSFGSNDEMVIGATFTR.V
6946	740 - 756	959.4440	1916.8734	1916.8727	0.35	0	55	3.3e-006	1	U	K.WSFGSNDEMVIGATFTR.V
6947	740 - 756	959.4440	1916.8734	1916.8727	0.35	0	90	9e-010	1	U	K.WSFGSNDEMVIGATFTR.V
6948	740 - 756	959.4441	1916.8736	1916.8727	0.48	0	27	0.0019	1	U	K.WSFGSNDEMVIGATFTR.V
6949	740 - 756	959.4442	1916.8738	1916.8727	0.54	0	68	1.7e-007	1	U	K.WSFGSNDEMVIGATFTR.V
6950	740 - 756	959.4442	1916.8739	1916.8727	0.61	0	69	1.2e-007	1	U	K.WSFGSNDEMVIGATFTR.V
6951	740 - 756	639.9653	1916.8740	1916.8727	0.66	0	25	0.0031	1	U	K.WSFGSNDEMVIGATFTR.V
6952	740 - 756	959.4444	1916.8742	1916.8727	0.80	0	27	0.0019	1	U	K.WSFGSNDEMVIGATFTR.V
6953	740 - 756	639.9656	1916.8749	1916.8727	1.14	0	43	5.4e-005	1	U	K.WSFGSNDEMVIGATFTR.V
6954	740 - 756	959.4448	1916.8750	1916.8727	1.18	0	46	2.6e-005	1	U	K.WSFGSNDEMVIGATFTR.V
6955	740 - 756	639.9656	1916.8751	1916.8727	1.24	0	49	1.4e-005	1	U	K.WSFGSNDEMVIGATFTR.V
6956	740 - 756	959.4448	1916.8751	1916.8727	1.24	0	68	1.6e-007	1	U	K.WSFGSNDEMVIGATFTR.V
6957	740 - 756	959.4448	1916.8751	1916.8727	1.24	0	85	3.1e-009	1	U	K.WSFGSNDEMVIGATFTR.V
6958	740 - 756	959.4449	1916.8752	1916.8727	1.31	0	67	1.9e-007	1	U	K.WSFGSNDEMVIGATFTR.V
6959	740 - 756	959.4449	1916.8753	1916.8727	1.37	0	59	1.2e-006	1	U	K.WSFGSNDEMVIGATFTR.V
6960	740 - 756	959.4450	1916.8755	1916.8727	1.44	0	78	1.8e-008	1	U	K.WSFGSNDEMVIGATFTR.V
6961	740 - 756	959.4451	1916.8757	1916.8727	1.56	0	49	1.2e-005	1	U	K.WSFGSNDEMVIGATFTR.V
6962	740 - 756	959.4452	1916.8758	1916.8727	1.63	0	72	6e-008	1	U	K.WSFGSNDEMVIGATFTR.V
6963	740 - 756	959.4453	1916.8759	1916.8727	1.69	0	42	6.2e-005	1	U	K.WSFGSNDEMVIGATFTR.V
6964	740 - 756	959.4454	1916.8763	1916.8727	1.88	0	68	1.6e-007	1	U	K.WSFGSNDEMVIGATFTR.V
6965	740 - 756	639.9661	1916.8764	1916.8727	1.91	0	9	0.11	1	U	K.WSFGSNDEMVIGATFTR.V
6966	740 - 756	959.4455	1916.8764	1916.8727	1.95	0	97	1.8e-010	1	U	K.WSFGSNDEMVIGATFTR.V
6967	740 - 756	959.4456	1916.8766	1916.8727	2.01	0	96	2.3e-010	1	U	K.WSFGSNDEMVIGATFTR.V
6968	740 - 756	959.4464	1916.8781	1916.8727	2.84	0	36	0.00024	1	U	K.WSFGSNDEMVIGATFTR.V
6969	740 - 756	959.4464	1916.8781	1916.8727	2.84	0	35	0.00033	1	U	K.WSFGSNDEMVIGATFTR.V
7024	740 - 756	967.4379	1932.8612	1932.8676	-3.34	0	42	5.6e-005	1	U	K.WSFGSNDEMVIGATFTR.V + Oxidation (M)
7025	740 - 756	967.4380	1932.8614	1932.8676	-3.21	0	65	3.1e-007	1	U	K.WSFGSNDEMVIGATFTR.V + Oxidation (M)
7026	740 - 756	967.4384	1932.8623	1932.8676	-2.77	0	17	0.021	1	U	K.WSFGSNDEMVIGATFTR.V + Oxidation (M)
7027	740 - 756	967.4386	1932.8626	1932.8676	-2.58	0	81	7.5e-009	1	U	K.WSFGSNDEMVIGATFTR.V + Oxidation (M)
7028	740 - 756	967.4390	1932.8634	1932.8676	-2.20	0	36	0.00027	1	U	K.WSFGSNDEMVIGATFTR.V + Oxidation (M)
7029	740 - 756	645.2952	1932.8639	1932.8676	-1.95	0	31	0.00077	1	U	K.WSFGSNDEMVIGATFTR.V + Oxidation (M)
7030	740 - 756	645.2953	1932.8642	1932.8676	-1.76	0	12	0.062	1	U	K.WSFGSNDEMVIGATFTR.V + Oxidation (M)
7031	740 - 756	967.4394	1932.8642	1932.8676	-1.76	0	70	1e-007	1	U	K.WSFGSNDEMVIGATFTR.V + Oxidation (M)
7032	740 - 756	967.4401	1932.8656	1932.8676	-1.06	0	68	1.4e-007	1	U	K.WSFGSNDEMVIGATFTR.V + Oxidation (M)
7033	740 - 756	967.4401	1932.8656	1932.8676	-1.06	0	50	1.1e-005	1	U	K.WSFGSNDEMVIGATFTR.V + Oxidation (M)
7034	740 - 756	967.4403	1932.8661	1932.8676	-0.81	0	52	6.3e-006	1	U	K.WSFGSNDEMVIGATFTR.V + Oxidation (M)
7035	740 - 756	967.4404	1932.8662	1932.8676	-0.75	0	60	1e-006	1	U	K.WSFGSNDEMVIGATFTR.V + Oxidation (M)
7036	740 - 756	967.4404	1932.8662	1932.8676	-0.75	0	58	1.6e-006	1	U	K.WSFGSNDEMVIGATFTR.V + Oxidation (M)
7037	740 - 756	645.2960	1932.8662	1932.8676	-0.72	0	49	1.1e-005	1	U	K.WSFGSNDEMVIGATFTR.V + Oxidation (M)
7038	740 - 756	967.4404	1932.8663	1932.8676	-0.68	0	43	5.5e-005	1	U	K.WSFGSNDEMVIGATFTR.V + Oxidation (M)
7039	740 - 756	645.2961	1932.8666	1932.8676	-0.53	0	19	0.011	1	U	K.WSFGSNDEMVIGATFTR.V + Oxidation (M)
7040	740 - 756	645.2962	1932.8668	1932.8676	-0.44	0	20	0.01	1	U	K.WSFGSNDEMVIGATFTR.V + Oxidation (M)
7041	740 - 756	967.4407	1932.8668	1932.8676	-0.43	0	32	0.00056	1	U	K.WSFGSNDEMVIGATFTR.V + Oxidation (M)

Query	Start - End	Observed	Mr(expt)	Mr(calc)	ppm	M	Score	Expect	Rank	U	Peptide
7042	740 - 756	967.4407	1932.8668	1932.8676	-0.43	0	31	0.00074	1	U	K.WSFGSNDEMVIGATFTR.V + Oxidation (M)
7043	740 - 756	645.2963	1932.8672	1932.8676	-0.25	0	36	0.00027	1	U	K.WSFGSNDEMVIGATFTR.V + Oxidation (M)
7044	740 - 756	645.2963	1932.8672	1932.8676	-0.25	0	46	2.3e-005	1	U	K.WSFGSNDEMVIGATFTR.V + Oxidation (M)
7045	740 - 756	967.4409	1932.8672	1932.8676	-0.24	0	56	2.8e-006	1	U	K.WSFGSNDEMVIGATFTR.V + Oxidation (M)
7046	740 - 756	645.2964	1932.8673	1932.8676	-0.15	0	8	0.17	1	U	K.WSFGSNDEMVIGATFTR.V + Oxidation (M)
7047	740 - 756	645.2964	1932.8673	1932.8676	-0.15	0	16	0.024	1	U	K.WSFGSNDEMVIGATFTR.V + Oxidation (M)
7048	740 - 756	967.4410	1932.8674	1932.8676	-0.12	0	36	0.00023	1	U	K.WSFGSNDEMVIGATFTR.V + Oxidation (M)
7049	740 - 756	967.4410	1932.8675	1932.8676	-0.053	0	51	8.9e-006	1	U	K.WSFGSNDEMVIGATFTR.V + Oxidation (M)
7050	740 - 756	967.4411	1932.8676	1932.8676	0.010	0	80	1e-008	1	U	K.WSFGSNDEMVIGATFTR.V + Oxidation (M)
7051	740 - 756	645.2965	1932.8677	1932.8676	0.036	0	27	0.0021	1	U	K.WSFGSNDEMVIGATFTR.V + Oxidation (M)
7052	740 - 756	967.4412	1932.8679	1932.8676	0.14	0	36	0.00025	1	U	K.WSFGSNDEMVIGATFTR.V + Oxidation (M)
7053	740 - 756	967.4412	1932.8679	1932.8676	0.14	0	49	1.3e-005	1	U	K.WSFGSNDEMVIGATFTR.V + Oxidation (M)
7054	740 - 756	967.4413	1932.8681	1932.8676	0.26	0	70	8.9e-008	1	U	K.WSFGSNDEMVIGATFTR.V + Oxidation (M)
7055	740 - 756	645.2967	1932.8682	1932.8676	0.32	0	11	0.077	1	U	K.WSFGSNDEMVIGATFTR.V + Oxidation (M)
7056	740 - 756	645.2967	1932.8682	1932.8676	0.32	0	8	0.17	1	U	K.WSFGSNDEMVIGATFTR.V + Oxidation (M)
7057	740 - 756	645.2967	1932.8682	1932.8676	0.32	0	9	0.14	1	U	K.WSFGSNDEMVIGATFTR.V + Oxidation (M)
7058	740 - 756	967.4414	1932.8683	1932.8676	0.33	0	54	4.2e-006	1	U	K.WSFGSNDEMVIGATFTR.V + Oxidation (M)
7059	740 - 756	967.4414	1932.8683	1932.8676	0.33	0	35	0.00035	1	U	K.WSFGSNDEMVIGATFTR.V + Oxidation (M)
7060	740 - 756	645.2968	1932.8684	1932.8676	0.41	0	37	0.00018	1	U	K.WSFGSNDEMVIGATFTR.V + Oxidation (M)
7061	740 - 756	967.4415	1932.8685	1932.8676	0.45	0	44	3.6e-005	1	U	K.WSFGSNDEMVIGATFTR.V + Oxidation (M)
7062	740 - 756	967.4416	1932.8687	1932.8676	0.58	0	98	1.6e-010	1	U	K.WSFGSNDEMVIGATFTR.V + Oxidation (M)
7063	740 - 756	967.4416	1932.8687	1932.8676	0.58	0	65	3.5e-007	1	U	K.WSFGSNDEMVIGATFTR.V + Oxidation (M)
7064	740 - 756	967.4416	1932.8687	1932.8676	0.58	0	47	2.2e-005	1	U	K.WSFGSNDEMVIGATFTR.V + Oxidation (M)
7065	740 - 756	967.4416	1932.8687	1932.8676	0.58	0	73	5e-008	1	U	K.WSFGSNDEMVIGATFTR.V + Oxidation (M)
7066	740 - 756	967.4417	1932.8689	1932.8676	0.64	0	53	5.2e-006	1	U	K.WSFGSNDEMVIGATFTR.V + Oxidation (M)
7067	740 - 756	645.2969	1932.8690	1932.8676	0.70	0	38	0.00017	1	U	K.WSFGSNDEMVIGATFTR.V + Oxidation (M)
7068	740 - 756	645.2969	1932.8690	1932.8676	0.70	0	34	0.00044	1	U	K.WSFGSNDEMVIGATFTR.V + Oxidation (M)
7069	740 - 756	967.4418	1932.8690	1932.8676	0.70	0	74	3.9e-008	1	U	K.WSFGSNDEMVIGATFTR.V + Oxidation (M)
7070	740 - 756	645.2970	1932.8692	1932.8676	0.79	0	44	4.2e-005	1	U	K.WSFGSNDEMVIGATFTR.V + Oxidation (M)
7071	740 - 756	645.2971	1932.8693	1932.8676	0.89	0	27	0.002	1	U	K.WSFGSNDEMVIGATFTR.V + Oxidation (M)
7072	740 - 756	645.2971	1932.8695	1932.8676	0.98	0	24	0.0041	1	U	K.WSFGSNDEMVIGATFTR.V + Oxidation (M)
7073	740 - 756	967.4421	1932.8697	1932.8676	1.08	0	57	2.1e-006	1	U	K.WSFGSNDEMVIGATFTR.V + Oxidation (M)
7074	740 - 756	645.2972	1932.8699	1932.8676	1.17	0	24	0.0042	1	U	K.WSFGSNDEMVIGATFTR.V + Oxidation (M)
7075	740 - 756	645.2972	1932.8699	1932.8676	1.17	0	23	0.0055	1	U	K.WSFGSNDEMVIGATFTR.V + Oxidation (M)
7076	740 - 756	645.2972	1932.8699	1932.8676	1.17	0	42	6.3e-005	1	U	K.WSFGSNDEMVIGATFTR.V + Oxidation (M)
7077	740 - 756	967.4423	1932.8700	1932.8676	1.21	0	66	2.7e-007	1	U	K.WSFGSNDEMVIGATFTR.V + Oxidation (M)
7078	740 - 756	645.2973	1932.8701	1932.8676	1.27	0	45	3.5e-005	1	U	K.WSFGSNDEMVIGATFTR.V + Oxidation (M)
7079	740 - 756	967.4423	1932.8701	1932.8676	1.27	0	34	0.0004	1	U	K.WSFGSNDEMVIGATFTR.V + Oxidation (M)
7080	740 - 756	967.4423	1932.8701	1932.8676	1.27	0	70	1e-007	1	U	K.WSFGSNDEMVIGATFTR.V + Oxidation (M)
7081	740 - 756	645.2974	1932.8703	1932.8676	1.36	0	21	0.0082	1	U	K.WSFGSNDEMVIGATFTR.V + Oxidation (M)
7082	740 - 756	645.2974	1932.8703	1932.8676	1.36	0	20	0.01	1	U	K.WSFGSNDEMVIGATFTR.V + Oxidation (M)
7083	740 - 756	645.2974	1932.8703	1932.8676	1.36	0	48	1.7e-005	1	U	K.WSFGSNDEMVIGATFTR.V + Oxidation (M)
7084	740 - 756	645.2974	1932.8704	1932.8676	1.46	0	6	0.23	1	U	K.WSFGSNDEMVIGATFTR.V + Oxidation (M)
7085	740 - 756	645.2975	1932.8706	1932.8676	1.55	0	5	0.3	1	U	K.WSFGSNDEMVIGATFTR.V + Oxidation (M)
7086	740 - 756	967.4426	1932.8707	1932.8676	1.59	0	35	0.00035	1	U	K.WSFGSNDEMVIGATFTR.V + Oxidation (M)
7087	740 - 756	967.4426	1932.8707	1932.8676	1.59	0	63	4.8e-007	1	U	K.WSFGSNDEMVIGATFTR.V + Oxidation (M)
7088	740 - 756	645.2976	1932.8710	1932.8676	1.74	0	28	0.0017	1	U	K.WSFGSNDEMVIGATFTR.V + Oxidation (M)
7089	740 - 756	645.2977	1932.8712	1932.8676	1.83	0	11	0.081	1	U	K.WSFGSNDEMVIGATFTR.V + Oxidation (M)
7090	740 - 756	645.2977	1932.8712	1932.8676	1.83	0	22	0.007	1	U	K.WSFGSNDEMVIGATFTR.V + Oxidation (M)
7091	740 - 756	645.2977	1932.8714	1932.8676	1.93	0	20	0.01	1	U	K.WSFGSNDEMVIGATFTR.V + Oxidation (M)
7092	740 - 756	645.2979	1932.8719	1932.8676	2.21	0	19	0.012	1	U	K.WSFGSNDEMVIGATFTR.V + Oxidation (M)
7093	740 - 756	967.4434	1932.8723	1932.8676	2.41	0	53	5.2e-006	1	U	K.WSFGSNDEMVIGATFTR.V + Oxidation (M)
7094	740 - 756	967.4435	1932.8724	1932.8676	2.47	0	59	1.3e-006	1	U	K.WSFGSNDEMVIGATFTR.V + Oxidation (M)
7095	740 - 756	645.2982	1932.8726	1932.8676	2.59	0	15	0.03	1	U	K.WSFGSNDEMVIGATFTR.V + Oxidation (M)
7096	740 - 756	967.4438	1932.8731	1932.8676	2.85	0	70	9e-008	1	U	K.WSFGSNDEMVIGATFTR.V + Oxidation (M)
7097	740 - 756	645.2985	1932.8736	1932.8676	3.07	0	13	0.054	1	U	K.WSFGSNDEMVIGATFTR.V + Oxidation (M)
7098	740 - 756	967.4445	1932.8744	1932.8676	3.48	0	44	4.4e-005	1	U	K.WSFGSNDEMVIGATFTR.V + Oxidation (M)
7099	740 - 756	967.4445	1932.8745	1932.8676	3.55	0	58	1.5e-006	1	U	K.WSFGSNDEMVIGATFTR.V + Oxidation (M)
73	757 - 761	259.1526	516.2907	516.2908	-0.14	0	19	0.013	1	U	R.VPSSK.I
1228	762 - 767	447.2534	892.4922	892.4919	0.30	1	9	0.12	1	U	K.IRYQWK.I
246	764 - 767	312.6603	623.3060	623.3067	-1.17	0	7	0.19	1	U	R.YQWK.I
247	764 - 767	312.6604	623.3063	623.3067	-0.68	0	15	0.032	1	U	R.YQWK.I
248	764 - 767	312.6606	623.3066	623.3067	-0.19	0	1	0.76	1	U	R.YQWK.I
249	764 - 767	312.6606	623.3066	623.3067	-0.19	0	16	0.023	1	U	R.YQWK.I
250	764 - 767	312.6606	623.3067	623.3067	0.0096	0	10	0.11	1	U	R.YQWK.I
251	764 - 767	312.6609	623.3072	623.3067	0.69	0	6	0.24	1	U	R.YQWK.I
252	764 - 767	312.6609	623.3073	623.3067	0.89	0	11	0.088	1	U	R.YQWK.I
4621	772 - 786	488.2802	1461.8189	1461.8191	-0.14	0	23	0.0047	1	U	R.LGSIIPAPPLDANGK.R
4623	772 - 786	731.9174	1461.8203	1461.8191	0.81	0	27	0.0018	1	U	R.LGSIIPAPPLDANGK.R
5300	772 - 787	405.4854	1617.9124	1617.9202	-4.81	1	16	0.027	1	U	R.LGSIIPAPPLDANGKR.T
5301	772 - 787	809.9641	1617.9137	1617.9202	-4.04	1	18	0.015	1	U	R.LGSIIPAPPLDANGKR.T
5302	772 - 787	540.3138	1617.9195	1617.9202	-0.43	1	21	0.0086	1	U	R.LGSIIPAPPLDANGKR.T
5304	772 - 787	809.9688	1617.9231	1617.9202	1.77	1	24	0.0044	1	U	R.LGSIIPAPPLDANGKR.T
5305	772 - 787	809.9692	1617.9238	1617.9202	2.22	1	8	0.17	1	U	R.LGSIIPAPPLDANGKR.T
5306	772 - 787	540.3163	1617.9270	1617.9202	4.21	1	27	0.0019	1	U	R.LGSIIPAPPLDANGKR.T
13112	787 - 813	1014.8553	3041.5440	3041.5379	2.01	2	67	1.9e-007	1	U	K.RTVTYGSITAITPMELYAVDEKNDNLK.V
13162	787 - 813	765.3887	3057.5256	3057.5328	-2.37	2	33	0.00045	1	U	K.RTVTYGSITAITPMELYAVDEKNDNLK.V + Oxidation (M)
13163	787 - 813	765.3918	3057.5380	3057.5328	1.70	2	15	0.035	1	U	K.RTVTYGSITAITPMELYAVDEKNDNLK.V + Oxidation (M)
13164	787 - 813	1020.1874	3057.5403	3057.5328	2.44	2	70	1e-007	1	U	K.RTVTYGSITAITPMELYAVDEKNDNLK.V + Oxidation (M)
9378	788 - 808	1151.5791	2301.1437	2301.1450	-0.59	0	10	0.11	1	U	R.TVTYGSITAITPMELYAVDEK.N
9379	788 - 808	768.0562	2301.1468	2301.1450	0.79	0	26	0.0027	1	U	R.TVTYGSITAITPMELYAVDEK.N
9380	788 - 808	768.0576	2301.1510	2301.1450	2.62	0	38	0.00017	1	U	R.TVTYGSITAITPMELYAVDEK.N
9426	788 - 808	1159.5758	2317.1371	2317.1399	-1.24	0	31	0.00074	1	U	R.TVTYGSITAITPMELYAVDEK.N + Oxidation (M)
9427	788 - 808	773.3875	2317.1407	2317.1399	0.34	0	26	0.0023	1	U	R.TVTYGSITAITPMELYAVDEK.N + Oxidation (M)

Query	Start - End	Observed	Mr(expt)	Mr(calc)	ppm	M	Score	Expect	Rank	U	Peptide
9428	788 - 808	1159.5782	2317.1419	2317.1399	0.87	0	42	7e-005	1	U	R.TVTYGSITAITPMELYAVDEK.N + Oxidation (M)
9429	788 - 808	773.3880	2317.1422	2317.1399	0.97	0	39	0.00012	1	U	R.TVTYGSITAITPMELYAVDEK.N + Oxidation (M)
9430	788 - 808	773.3881	2317.1424	2317.1399	1.05	0	46	2.4e-005	1	U	R.TVTYGSITAITPMELYAVDEK.N + Oxidation (M)
9431	788 - 808	1159.5786	2317.1427	2317.1399	1.19	0	56	2.3e-006	1	U	R.TVTYGSITAITPMELYAVDEK.N + Oxidation (M)
9432	788 - 808	773.3882	2317.1429	2317.1399	1.29	0	40	0.00011	1	U	R.TVTYGSITAITPMELYAVDEK.N + Oxidation (M)
9433	788 - 808	1159.5793	2317.1441	2317.1399	1.82	0	34	0.00037	1	U	R.TVTYGSITAITPMELYAVDEK.N + Oxidation (M)
9434	788 - 808	1159.5795	2317.1444	2317.1399	1.93	0	16	0.026	1	U	R.TVTYGSITAITPMELYAVDEK.N + Oxidation (M)
9435	788 - 808	1159.5797	2317.1449	2317.1399	2.14	0	11	0.086	1	U	R.TVTYGSITAITPMELYAVDEK.N + Oxidation (M)
9436	788 - 808	773.3890	2317.1451	2317.1399	2.24	0	29	0.0012	1	U	R.TVTYGSITAITPMELYAVDEK.N + Oxidation (M)
9437	788 - 808	773.3890	2317.1453	2317.1399	2.31	0	44	3.6e-005	1	U	R.TVTYGSITAITPMELYAVDEK.N + Oxidation (M)
9438	788 - 808	773.3890	2317.1453	2317.1399	2.31	0	22	0.0058	1	U	R.TVTYGSITAITPMELYAVDEK.N + Oxidation (M)
9439	788 - 808	1159.5804	2317.1463	2317.1399	2.77	0	26	0.0024	1	U	R.TVTYGSITAITPMELYAVDEK.N + Oxidation (M)
9440	788 - 808	773.3900	2317.1482	2317.1399	3.58	0	36	0.00024	1	U	R.TVTYGSITAITPMELYAVDEK.N + Oxidation (M)
12378	788 - 813	962.8171	2885.4296	2885.4368	-2.50	1	21	0.0076	1	U	R.TVTYGSITAITPMELYAVDEKNDNLK.V
12379	788 - 813	962.8189	2885.4349	2885.4368	-0.66	1	48	1.5e-005	1	U	R.TVTYGSITAITPMELYAVDEKNDNLK.V
12380	788 - 813	962.8192	2885.4356	2885.4368	-0.41	1	9	0.12	1	U	R.TVTYGSITAITPMELYAVDEKNDNLK.V
12382	788 - 813	962.8195	2885.4365	2885.4368	-0.093	1	35	0.00032	1	U	R.TVTYGSITAITPMELYAVDEKNDNLK.V
12383	788 - 813	962.8196	2885.4369	2885.4368	0.034	1	1	0.87	1	U	R.TVTYGSITAITPMELYAVDEKNDNLK.V
12384	788 - 813	962.8201	2885.4386	2885.4368	0.61	1	29	0.0014	1	U	R.TVTYGSITAITPMELYAVDEKNDNLK.V
12385	788 - 813	962.8203	2885.4391	2885.4368	0.80	1	41	8e-005	1	U	R.TVTYGSITAITPMELYAVDEKNDNLK.V
12386	788 - 813	722.3671	2885.4394	2885.4368	0.90	1	31	0.00074	1	U	R.TVTYGSITAITPMELYAVDEKNDNLK.V
12387	788 - 813	962.8207	2885.4404	2885.4368	1.24	1	8	0.15	1	U	R.TVTYGSITAITPMELYAVDEKNDNLK.V
12388	788 - 813	1443.7277	2885.4408	2885.4368	1.37	1	63	4.8e-007	1	U	R.TVTYGSITAITPMELYAVDEKNDNLK.V
12389	788 - 813	962.8209	2885.4409	2885.4368	1.43	1	37	0.0002	1	U	R.TVTYGSITAITPMELYAVDEKNDNLK.V
12542	788 - 813	726.3624	2901.4204	2901.4317	-3.92	1	26	0.0025	1	U	R.TVTYGSITAITPMELYAVDEKNDNLK.V + Oxidation (M)
12543	788 - 813	968.1513	2901.4321	2901.4317	0.12	1	32	0.00056	1	U	R.TVTYGSITAITPMELYAVDEKNDNLK.V + Oxidation (M)
12544	788 - 813	968.1530	2901.4370	2901.4317	1.83	1	24	0.0036	1	U	R.TVTYGSITAITPMELYAVDEKNDNLK.V + Oxidation (M)
12545	788 - 813	726.3666	2901.4372	2901.4317	1.89	1	29	0.0011	1	U	R.TVTYGSITAITPMELYAVDEKNDNLK.V + Oxidation (M)
12546	788 - 813	726.3666	2901.4372	2901.4317	1.89	1	27	0.0022	1	U	R.TVTYGSITAITPMELYAVDEKNDNLK.V + Oxidation (M)
4486	814 - 826	715.8899	1429.7652	1429.7664	-0.81	1	68	1.6e-007	1	U	K.VKVAELGESEIEK.V
4487	814 - 826	477.5962	1429.7668	1429.7664	0.31	1	53	5.6e-006	1	U	K.VKVAELGESEIEK.V
9145	814 - 833	747.7304	2240.1694	2240.1722	-1.25	2	35	0.00029	1	U	K.VKVAELGESEIEKVMIDAHK.F + Oxidation (M)
9146	814 - 833	561.0502	2240.1716	2240.1722	-0.27	2	26	0.0022	1	U	K.VKVAELGESEIEKVMIDAHK.F + Oxidation (M)
3128	816 - 826	602.3060	1202.5975	1202.6030	-4.58	0	14	0.036	1	U	K.VAELGESEIEK.V
3129	816 - 826	602.3076	1202.6006	1202.6030	-2.04	0	46	2.7e-005	1	U	K.VAELGESEIEK.V
3130	816 - 826	602.3080	1202.6015	1202.6030	-1.23	0	12	0.066	1	U	K.VAELGESEIEK.V
3131	816 - 826	602.3081	1202.6017	1202.6030	-1.13	0	8	0.15	1	U	K.VAELGESEIEK.V
3132	816 - 826	602.3081	1202.6017	1202.6030	-1.13	0	11	0.072	1	U	K.VAELGESEIEK.V
3133	816 - 826	602.3082	1202.6019	1202.6030	-0.92	0	55	3.5e-006	1	U	K.VAELGESEIEK.V
3134	816 - 826	602.3084	1202.6023	1202.6030	-0.62	0	52	5.8e-006	1	U	K.VAELGESEIEK.V
3135	816 - 826	602.3085	1202.6024	1202.6030	-0.52	0	13	0.049	1	U	K.VAELGESEIEK.V
3136	816 - 826	602.3085	1202.6024	1202.6030	-0.52	0	6	0.24	1	U	K.VAELGESEIEK.V
3137	816 - 826	602.3085	1202.6024	1202.6030	-0.52	0	34	0.00037	1	U	K.VAELGESEIEK.V
3138	816 - 826	602.3087	1202.6028	1202.6030	-0.21	0	42	6.5e-005	1	U	K.VAELGESEIEK.V
3139	816 - 826	602.3087	1202.6028	1202.6030	-0.21	0	20	0.01	1	U	K.VAELGESEIEK.V
3140	816 - 826	602.3088	1202.6031	1202.6030	0.093	0	17	0.019	1	U	K.VAELGESEIEK.V
3141	816 - 826	602.3089	1202.6032	1202.6030	0.19	0	4	0.43	1	U	K.VAELGESEIEK.V
3142	816 - 826	602.3089	1202.6032	1202.6030	0.19	0	39	0.00012	1	U	K.VAELGESEIEK.V
3143	816 - 826	602.3090	1202.6034	1202.6030	0.30	0	13	0.046	1	U	K.VAELGESEIEK.V
3144	816 - 826	602.3090	1202.6034	1202.6030	0.30	0	14	0.044	1	U	K.VAELGESEIEK.V
3145	816 - 826	602.3090	1202.6034	1202.6030	0.30	0	18	0.015	1	U	K.VAELGESEIEK.V
3146	816 - 826	602.3090	1202.6034	1202.6030	0.30	0	38	0.00017	1	U	K.VAELGESEIEK.V
3147	816 - 826	602.3091	1202.6036	1202.6030	0.50	0	7	0.2	1	U	K.VAELGESEIEK.V
3148	816 - 826	602.3091	1202.6036	1202.6030	0.50	0	47	1.8e-005	1	U	K.VAELGESEIEK.V
3149	816 - 826	602.3091	1202.6036	1202.6030	0.50	0	30	0.00096	1	U	K.VAELGESEIEK.V
3150	816 - 826	602.3092	1202.6039	1202.6030	0.70	0	30	0.0011	1	U	K.VAELGESEIEK.V
3151	816 - 826	602.3092	1202.6039	1202.6030	0.70	0	30	0.0011	1	U	K.VAELGESEIEK.V
3152	816 - 826	602.3092	1202.6039	1202.6030	0.70	0	41	7.7e-005	1	U	K.VAELGESEIEK.V
3153	816 - 826	602.3093	1202.6040	1202.6030	0.80	0	7	0.19	1	U	K.VAELGESEIEK.V
3154	816 - 826	602.3094	1202.6042	1202.6030	1.01	0	18	0.017	1	U	K.VAELGESEIEK.V
3155	816 - 826	602.3094	1202.6042	1202.6030	1.01	0	19	0.012	1	U	K.VAELGESEIEK.V
3156	816 - 826	602.3094	1202.6042	1202.6030	1.01	0	19	0.012	1	U	K.VAELGESEIEK.V
3157	816 - 826	602.3094	1202.6043	1202.6030	1.11	0	20	0.011	1	U	K.VAELGESEIEK.V
3158	816 - 826	602.3094	1202.6043	1202.6030	1.11	0	25	0.0033	1	U	K.VAELGESEIEK.V
3159	816 - 826	602.3097	1202.6048	1202.6030	1.51	0	14	0.036	1	U	K.VAELGESEIEK.V
3160	816 - 826	602.3098	1202.6051	1202.6030	1.72	0	22	0.0062	1	U	K.VAELGESEIEK.V
3161	816 - 826	602.3099	1202.6053	1202.6030	1.92	0	12	0.06	1	U	K.VAELGESEIEK.V
3162	816 - 826	602.3102	1202.6058	1202.6030	2.33	0	29	0.0011	1	U	K.VAELGESEIEK.V
3163	816 - 826	602.3103	1202.6061	1202.6030	2.53	0	25	0.003	1	U	K.VAELGESEIEK.V
7722	816 - 833	500.2614	1997.0164	1997.0139	1.27	1	40	9.3e-005	1	U	K.VAELGESEIEKVMIDAHK.F
7723	816 - 833	666.6802	1997.0187	1997.0139	2.40	1	33	0.00048	1	U	K.VAELGESEIEKVMIDAHK.F
7836	816 - 833	504.2570	2012.9991	2013.0088	-4.83	1	20	0.011	1	U	K.VAELGESEIEKVMIDAHK.F + Oxidation (M)
7837	816 - 833	672.0074	2013.0005	2013.0088	-4.13	1	39	0.00014	1	U	K.VAELGESEIEKVMIDAHK.F + Oxidation (M)
7838	816 - 833	504.2587	2013.0058	2013.0088	-1.50	1	13	0.047	1	U	K.VAELGESEIEKVMIDAHK.F + Oxidation (M)
7839	816 - 833	672.0094	2013.0064	2013.0088	-1.22	1	35	0.0003	1	U	K.VAELGESEIEKVMIDAHK.F + Oxidation (M)
7840	816 - 833	504.2592	2013.0075	2013.0088	-0.65	1	26	0.0024	1	U	K.VAELGESEIEKVMIDAHK.F + Oxidation (M)
7841	816 - 833	672.0098	2013.0077	2013.0088	-0.58	1	11	0.084	1	U	K.VAELGESEIEKVMIDAHK.F + Oxidation (M)
7842	816 - 833	672.0098	2013.0077	2013.0088	-0.58	1	17	0.022	1	U	K.VAELGESEIEKVMIDAHK.F + Oxidation (M)
7843	816 - 833	672.0099	2013.0078	2013.0088	-0.49	1	15	0.034	1	U	K.VAELGESEIEKVMIDAHK.F + Oxidation (M)
7844	816 - 833	504.2593	2013.0080	2013.0088	-0.41	1	12	0.058	1	U	K.VAELGESEIEKVMIDAHK.F + Oxidation (M)
7845	816 - 833	504.2593	2013.0081	2013.0088	-0.34	1	20	0.0092	1	U	K.VAELGESEIEKVMIDAHK.F + Oxidation (M)
7846	816 - 833	1007.5114	2013.0082	2013.0088	-0.33	1	56	2.6e-006	1	U	K.VAELGESEIEKVMIDAHK.F + Oxidation (M)
7847	816 - 833	672.0100	2013.0082	2013.0088	-0.31	1	33	0.00055	1	U	K.VAELGESEIEKVMIDAHK.F + Oxidation (M)
7848	816 - 833	672.0100	2013.0082	2013.0088	-0.31	1	45	3.5e-005	1	U	K.VAELGESEIEKVMIDAHK.F + Oxidation (M)
7849	816 - 833	504.2594	2013.0084	2013.0088	-0.22	1	9	0.13	1	U	K.VAELGESEIEKVMIDAHK.F + Oxidation (M)
7850	816 - 833	672.0101	2013.0084	2013.0088	-0.22	1	13	0.054	1	U	K.VAELGESEIEKVMIDAHK.F + Oxidation (M)
7851	816 - 833	672.0101	2013.0084	2013.0088	-0.22	1	33	0.00048	1	U	K.VAELGESEIEKVMIDAHK.F + Oxidation (M)
7852	816 - 833	672.0101	2013.0086	2013.0088	-0.13	1	34	0.00036	1	U	K.VAELGESEIEKVMIDAHK.F + Oxidation (M)

Query	Start - End	Observed	Mr(expt)	Mr(calc)	ppm	M	Score	Expect	Rank	U	Peptide
7853	816 - 833	672.0101	2013.0086	2013.0088	-0.13	1	17	0.022	1	U	K.VAELGESEIEKVMIDAHK.F + Oxidation (M)
7854	816 - 833	504.2594	2013.0086	2013.0088	-0.10	1	13	0.055	1	U	K.VAELGESEIEKVMIDAHK.F + Oxidation (M)
7855	816 - 833	504.2595	2013.0087	2013.0088	-0.042	1	9	0.14	1	U	K.VAELGESEIEKVMIDAHK.F + Oxidation (M)
7856	816 - 833	672.0102	2013.0088	2013.0088	-0.034	1	17	0.022	1	U	K.VAELGESEIEKVMIDAHK.F + Oxidation (M)
7857	816 - 833	672.0102	2013.0088	2013.0088	-0.034	1	37	0.0002	1	U	K.VAELGESEIEKVMIDAHK.F + Oxidation (M)
7858	816 - 833	672.0102	2013.0088	2013.0088	-0.034	1	13	0.051	1	U	K.VAELGESEIEKVMIDAHK.F + Oxidation (M)
7859	816 - 833	504.2595	2013.0089	2013.0088	0.020	1	22	0.0057	1	U	K.VAELGESEIEKVMIDAHK.F + Oxidation (M)
7860	816 - 833	504.2596	2013.0091	2013.0088	0.14	1	17	0.019	1	U	K.VAELGESEIEKVMIDAHK.F + Oxidation (M)
7861	816 - 833	504.2596	2013.0091	2013.0088	0.14	1	20	0.011	1	U	K.VAELGESEIEKVMIDAHK.F + Oxidation (M)
7862	816 - 833	504.2596	2013.0091	2013.0088	0.14	1	17	0.02	1	U	K.VAELGESEIEKVMIDAHK.F + Oxidation (M)
7863	816 - 833	504.2596	2013.0092	2013.0088	0.20	1	17	0.019	1	U	K.VAELGESEIEKVMIDAHK.F + Oxidation (M)
7864	816 - 833	672.0104	2013.0093	2013.0088	0.24	1	9	0.12	1	U	K.VAELGESEIEKVMIDAHK.F + Oxidation (M)
7866	816 - 833	672.0104	2013.0093	2013.0088	0.24	1	11	0.081	1	U	K.VAELGESEIEKVMIDAHK.F + Oxidation (M)
7867	816 - 833	504.2596	2013.0093	2013.0088	0.26	1	22	0.0059	1	U	K.VAELGESEIEKVMIDAHK.F + Oxidation (M)
7868	816 - 833	504.2597	2013.0096	2013.0088	0.38	1	28	0.0017	1	U	K.VAELGESEIEKVMIDAHK.F + Oxidation (M)
7869	816 - 833	504.2597	2013.0096	2013.0088	0.38	1	11	0.072	1	U	K.VAELGESEIEKVMIDAHK.F + Oxidation (M)
7870	816 - 833	504.2597	2013.0098	2013.0088	0.50	1	11	0.089	1	U	K.VAELGESEIEKVMIDAHK.F + Oxidation (M)
7871	816 - 833	504.2597	2013.0098	2013.0088	0.50	1	16	0.023	1	U	K.VAELGESEIEKVMIDAHK.F + Oxidation (M)
7872	816 - 833	672.0106	2013.0098	2013.0088	0.51	1	12	0.06	1	U	K.VAELGESEIEKVMIDAHK.F + Oxidation (M)
7873	816 - 833	504.2598	2013.0100	2013.0088	0.57	1	18	0.017	1	U	K.VAELGESEIEKVMIDAHK.F + Oxidation (M)
7874	816 - 833	504.2598	2013.0100	2013.0088	0.57	1	13	0.051	1	U	K.VAELGESEIEKVMIDAHK.F + Oxidation (M)
7875	816 - 833	672.0106	2013.0100	2013.0088	0.60	1	0	0.99	1	U	K.VAELGESEIEKVMIDAHK.F + Oxidation (M)
7876	816 - 833	672.0106	2013.0100	2013.0088	0.60	1	15	0.028	1	U	K.VAELGESEIEKVMIDAHK.F + Oxidation (M)
7877	816 - 833	504.2598	2013.0101	2013.0088	0.63	1	12	0.058	1	U	K.VAELGESEIEKVMIDAHK.F + Oxidation (M)
7878	816 - 833	504.2598	2013.0101	2013.0088	0.63	1	7	0.19	1	U	K.VAELGESEIEKVMIDAHK.F + Oxidation (M)
7879	816 - 833	504.2598	2013.0101	2013.0088	0.63	1	20	0.01	1	U	K.VAELGESEIEKVMIDAHK.F + Oxidation (M)
7880	816 - 833	504.2598	2013.0101	2013.0088	0.63	1	14	0.036	1	U	K.VAELGESEIEKVMIDAHK.F + Oxidation (M)
7881	816 - 833	504.2598	2013.0102	2013.0088	0.69	1	18	0.016	1	U	K.VAELGESEIEKVMIDAHK.F + Oxidation (M)
7882	816 - 833	672.0107	2013.0102	2013.0088	0.69	1	11	0.081	1	U	K.VAELGESEIEKVMIDAHK.F + Oxidation (M)
7883	816 - 833	672.0107	2013.0102	2013.0088	0.69	1	19	0.011	1	U	K.VAELGESEIEKVMIDAHK.F + Oxidation (M)
7884	816 - 833	504.2599	2013.0103	2013.0088	0.75	1	12	0.057	1	U	K.VAELGESEIEKVMIDAHK.F + Oxidation (M)
7885	816 - 833	672.0107	2013.0104	2013.0088	0.78	1	41	7.3e-005	1	U	K.VAELGESEIEKVMIDAHK.F + Oxidation (M)
7886	816 - 833	672.0107	2013.0104	2013.0088	0.78	1	20	0.011	1	U	K.VAELGESEIEKVMIDAHK.F + Oxidation (M)
7887	816 - 833	504.2599	2013.0104	2013.0088	0.81	1	31	0.00086	1	U	K.VAELGESEIEKVMIDAHK.F + Oxidation (M)
7889	816 - 833	672.0108	2013.0106	2013.0088	0.87	1	3	0.48	1	U	K.VAELGESEIEKVMIDAHK.F + Oxidation (M)
7891	816 - 833	672.0108	2013.0106	2013.0088	0.87	1	20	0.01	1	U	K.VAELGESEIEKVMIDAHK.F + Oxidation (M)
7892	816 - 833	672.0109	2013.0108	2013.0088	0.97	1	11	0.077	1	U	K.VAELGESEIEKVMIDAHK.F + Oxidation (M)
7893	816 - 833	504.2600	2013.0108	2013.0088	0.99	1	16	0.025	1	U	K.VAELGESEIEKVMIDAHK.F + Oxidation (M)
7894	816 - 833	504.2600	2013.0109	2013.0088	1.05	1	10	0.1	1	U	K.VAELGESEIEKVMIDAHK.F + Oxidation (M)
7895	816 - 833	672.0109	2013.0109	2013.0088	1.06	1	30	0.00095	1	U	K.VAELGESEIEKVMIDAHK.F + Oxidation (M)
7896	816 - 833	504.2601	2013.0112	2013.0088	1.17	1	16	0.024	1	U	K.VAELGESEIEKVMIDAHK.F + Oxidation (M)
7897	816 - 833	672.0110	2013.0113	2013.0088	1.24	1	6	0.24	1	U	K.VAELGESEIEKVMIDAHK.F + Oxidation (M)
7898	816 - 833	672.0110	2013.0113	2013.0088	1.24	1	38	0.00017	1	U	K.VAELGESEIEKVMIDAHK.F + Oxidation (M)
7899	816 - 833	672.0110	2013.0113	2013.0088	1.24	1	19	0.013	1	U	K.VAELGESEIEKVMIDAHK.F + Oxidation (M)
7900	816 - 833	672.0110	2013.0113	2013.0088	1.24	1	13	0.05	1	U	K.VAELGESEIEKVMIDAHK.F + Oxidation (M)
7901	816 - 833	672.0112	2013.0119	2013.0088	1.51	1	13	0.054	1	U	K.VAELGESEIEKVMIDAHK.F + Oxidation (M)
7902	816 - 833	672.0114	2013.0122	2013.0088	1.69	1	30	0.0011	1	U	K.VAELGESEIEKVMIDAHK.F + Oxidation (M)
7903	816 - 833	672.0114	2013.0122	2013.0088	1.69	1	13	0.054	1	U	K.VAELGESEIEKVMIDAHK.F + Oxidation (M)
7905	816 - 833	672.0115	2013.0126	2013.0088	1.88	1	13	0.052	1	U	K.VAELGESEIEKVMIDAHK.F + Oxidation (M)
7906	816 - 833	672.0115	2013.0128	2013.0088	1.97	1	31	0.00074	1	U	K.VAELGESEIEKVMIDAHK.F + Oxidation (M)
7907	816 - 833	672.0115	2013.0128	2013.0088	1.97	1	33	0.00054	1	U	K.VAELGESEIEKVMIDAHK.F + Oxidation (M)
7909	816 - 833	672.0119	2013.0139	2013.0088	2.51	1	1	0.89	1	U	K.VAELGESEIEKVMIDAHK.F + Oxidation (M)
7910	816 - 833	672.0120	2013.0141	2013.0088	2.60	1	8	0.18	1	U	K.VAELGESEIEKVMIDAHK.F + Oxidation (M)
7912	816 - 833	672.0120	2013.0142	2013.0088	2.69	1	7	0.2	1	U	K.VAELGESEIEKVMIDAHK.F + Oxidation (M)
7913	816 - 833	672.0121	2013.0146	2013.0088	2.88	1	13	0.05	1	U	K.VAELGESEIEKVMIDAHK.F + Oxidation (M)
7915	816 - 833	672.0123	2013.0150	2013.0088	3.06	1	7	0.2	1	U	K.VAELGESEIEKVMIDAHK.F + Oxidation (M)
847	827 - 833	271.8144	812.4214	812.4214	-0.0086	0	4	0.39	1	U	K.VMIDAHK.F
848	827 - 833	407.2180	812.4215	812.4214	0.044	0	25	0.0032	1	U	K.VMIDAHK.F
849	827 - 833	271.8145	812.4216	812.4214	0.22	0	16	0.026	1	U	K.VMIDAHK.F
850	827 - 833	407.2182	812.4219	812.4214	0.57	0	19	0.014	1	U	K.VMIDAHK.F
915	827 - 833	415.2150	828.4154	828.4164	-1.19	0	17	0.018	1	U	K.VMIDAHK.F + Oxidation (M)
916	827 - 833	415.2153	828.4161	828.4164	-0.38	0	25	0.0033	1	U	K.VMIDAHK.F + Oxidation (M)
917	827 - 833	415.2154	828.4162	828.4164	-0.23	0	34	0.0004	1	U	K.VMIDAHK.F + Oxidation (M)
918	827 - 833	415.2155	828.4164	828.4164	-0.013	0	37	0.00022	1	U	K.VMIDAHK.F + Oxidation (M)
919	827 - 833	277.1461	828.4164	828.4164	0.083	0	24	0.0037	1	U	K.VMIDAHK.F + Oxidation (M)
920	827 - 833	277.1461	828.4164	828.4164	0.083	0	33	0.00055	1	U	K.VMIDAHK.F + Oxidation (M)
921	827 - 833	415.2155	828.4165	828.4164	0.21	0	28	0.0015	1	U	K.VMIDAHK.F + Oxidation (M)
922	827 - 833	277.1461	828.4166	828.4164	0.30	0	24	0.0044	1	U	K.VMIDAHK.F + Oxidation (M)
923	827 - 833	277.1461	828.4166	828.4164	0.30	0	18	0.017	1	U	K.VMIDAHK.F + Oxidation (M)
924	827 - 833	415.2166	828.4187	828.4164	2.79	0	2	0.64	1	U	K.VMIDAHK.F + Oxidation (M)
10654	834 - 854	1262.6093	2523.2040	2523.2110	-2.80	0	6	0.23	1	U	K.FSGWWYLSENPNLYSGLSLYK.L
10655	834 - 854	842.0757	2523.2054	2523.2110	-2.23	0	2	0.57	1	U	K.FSGWWYLSENPNLYSGLSLYK.L
10656	834 - 854	1262.6106	2523.2066	2523.2110	-1.74	0	2	0.61	1	U	K.FSGWWYLSENPNLYSGLSLYK.L
10657	834 - 854	631.8096	2523.2092	2523.2110	-0.73	0	31	0.00075	1	U	K.FSGWWYLSENPNLYSGLSLYK.L
10658	834 - 854	842.0770	2523.2092	2523.2110	-0.71	0	13	0.049	1	U	K.FSGWWYLSENPNLYSGLSLYK.L
10659	834 - 854	842.0771	2523.2094	2523.2110	-0.63	0	60	9e-007	1	U	K.FSGWWYLSENPNLYSGLSLYK.L
10660	834 - 854	842.0771	2523.2096	2523.2110	-0.56	0	43	5.1e-005	1	U	K.FSGWWYLSENPNLYSGLSLYK.L
10661	834 - 854	842.0773	2523.2102	2523.2110	-0.34	0	14	0.04	1	U	K.FSGWWYLSENPNLYSGLSLYK.L
10662	834 - 854	842.0775	2523.2105	2523.2110	-0.20	0	50	1.1e-005	1	U	K.FSGWWYLSENPNLYSGLSLYK.L
10665	834 - 854	1262.6130	2523.2115	2523.2110	0.20	0	25	0.0032	1	U	K.FSGWWYLSENPNLYSGLSLYK.L
10667	834 - 854	842.0782	2523.2127	2523.2110	0.67	0	15	0.031	1	U	K.FSGWWYLSENPNLYSGLSLYK.L
10668	834 - 854	1262.6138	2523.2130	2523.2110	0.78	0	28	0.0016	1	U	K.FSGWWYLSENPNLYSGLSLYK.L
10669	834 - 854	842.0786	2523.2138	2523.2110	1.11	0	34	0.00042	1	U	K.FSGWWYLSENPNLYSGLSLYK.L
10670	834 - 854	842.0789	2523.2147	2523.2110	1.47	0	54	3.9e-006	1	U	K.FSGWWYLSENPNLYSGLSLYK.L
10671	834 - 854	842.0790	2523.2151	2523.2110	1.62	0	32	0.00058	1	U	K.FSGWWYLSENPNLYSGLSLYK.L
10672	834 - 854	1262.6149	2523.2152	2523.2110	1.65	0	63	5.4e-007	1	U	K.FSGWWYLSENPNLYSGLSLYK.L
4972	855 - 867	780.3884	1558.7623	1558.7667	-2.85	0	52	6.9e-006	1	U	K.LPDIFYNNVSSYK.I

Query	Start - End	Observed	Mr(expt)	Mr(calc)	ppm	M	Score	Expect	Rank	U	Peptide
4973	855 - 867	780.3893	1558.7640	1558.7667	-1.75	0	47	2e-005	1	U	K.LPDIFYNNVSSYK.I
4974	855 - 867	780.3895	1558.7645	1558.7667	-1.44	0	54	3.7e-006	1	U	K.LPDIFYNNVSSYK.I
4975	855 - 867	780.3896	1558.7647	1558.7667	-1.28	0	36	0.00023	1	U	K.LPDIFYNNVSSYK.I
4976	855 - 867	780.3901	1558.7657	1558.7667	-0.65	0	60	1e-006	1	U	K.LPDIFYNNVSSYK.I
4977	855 - 867	780.3903	1558.7661	1558.7667	-0.42	0	46	2.6e-005	1	U	K.LPDIFYNNVSSYK.I
4978	855 - 867	780.3903	1558.7661	1558.7667	-0.42	0	46	2.5e-005	1	U	K.LPDIFYNNVSSYK.I
4979	855 - 867	780.3904	1558.7662	1558.7667	-0.34	0	49	1.1e-005	1	U	K.LPDIFYNNVSSYK.I
4980	855 - 867	780.3906	1558.7666	1558.7667	-0.10	0	38	0.00016	1	U	K.LPDIFYNNVSSYK.I
4981	855 - 867	780.3907	1558.7668	1558.7667	0.052	0	31	0.00083	1	U	K.LPDIFYNNVSSYK.I
4982	855 - 867	780.3907	1558.7669	1558.7667	0.13	0	35	0.00028	1	U	K.LPDIFYNNVSSYK.I
4983	855 - 867	780.3907	1558.7669	1558.7667	0.13	0	28	0.0015	1	U	K.LPDIFYNNVSSYK.I
4984	855 - 867	780.3909	1558.7672	1558.7667	0.29	0	43	4.6e-005	1	U	K.LPDIFYNNVSSYK.I
4985	855 - 867	780.3910	1558.7674	1558.7667	0.44	0	55	3.1e-006	1	U	K.LPDIFYNNVSSYK.I
4986	855 - 867	780.3914	1558.7683	1558.7667	0.99	0	52	7e-006	1	U	K.LPDIFYNNVSSYK.I
4987	855 - 867	780.3915	1558.7684	1558.7667	1.07	0	29	0.0013	1	U	K.LPDIFYNNVSSYK.I
4988	855 - 867	780.3915	1558.7685	1558.7667	1.15	0	19	0.013	1	U	K.LPDIFYNNVSSYK.I
4989	855 - 867	780.3916	1558.7687	1558.7667	1.23	0	46	2.5e-005	1	U	K.LPDIFYNNVSSYK.I
4990	855 - 867	780.3916	1558.7687	1558.7667	1.23	0	50	1e-005	1	U	K.LPDIFYNNVSSYK.I
4991	855 - 867	780.3917	1558.7689	1558.7667	1.38	0	29	0.0013	1	U	K.LPDIFYNNVSSYK.I
4992	855 - 867	780.3917	1558.7689	1558.7667	1.38	0	56	2.6e-006	1	U	K.LPDIFYNNVSSYK.I
4993	855 - 867	780.3918	1558.7690	1558.7667	1.46	0	26	0.0026	1	U	K.LPDIFYNNVSSYK.I
4994	855 - 867	780.3918	1558.7690	1558.7667	1.46	0	40	9.2e-005	1	U	K.LPDIFYNNVSSYK.I
4995	855 - 867	780.3920	1558.7694	1558.7667	1.70	0	75	3.5e-008	1	U	K.LPDIFYNNVSSYK.I
4996	855 - 867	780.3920	1558.7694	1558.7667	1.70	0	27	0.0021	1	U	K.LPDIFYNNVSSYK.I
4997	855 - 867	780.3922	1558.7697	1558.7667	1.93	0	42	5.7e-005	1	U	K.LPDIFYNNVSSYK.I
4998	855 - 867	780.3922	1558.7699	1558.7667	2.01	0	38	0.00014	1	U	K.LPDIFYNNVSSYK.I
4999	855 - 867	780.3923	1558.7701	1558.7667	2.17	0	57	1.8e-006	1	U	K.LPDIFYNNVSSYK.I
5000	855 - 867	780.3923	1558.7701	1558.7667	2.17	0	33	0.00053	1	U	K.LPDIFYNNVSSYK.I
5001	855 - 867	780.3924	1558.7702	1558.7667	2.24	0	38	0.00014	1	U	K.LPDIFYNNVSSYK.I
5002	855 - 867	780.3925	1558.7704	1558.7667	2.32	0	32	0.0006	1	U	K.LPDIFYNNVSSYK.I
5003	855 - 867	780.3925	1558.7704	1558.7667	2.32	0	55	3.3e-006	1	U	K.LPDIFYNNVSSYK.I
5004	855 - 867	780.3925	1558.7705	1558.7667	2.40	0	9	0.12	1	U	K.LPDIFYNNVSSYK.I
5005	855 - 867	780.3927	1558.7708	1558.7667	2.64	0	32	0.00069	1	U	K.LPDIFYNNVSSYK.I
5006	855 - 867	780.3930	1558.7715	1558.7667	3.03	0	51	7.9e-006	1	U	K.LPDIFYNNVSSYK.I
5007	855 - 867	780.3932	1558.7718	1558.7667	3.26	0	58	1.5e-006	1	U	K.LPDIFYNNVSSYK.I
6591	855 - 869	610.3247	1827.9523	1827.9519	0.21	1	18	0.014	1	U	K.LPDIFYNNVSSYKIR.V
9462	874 - 893	581.3365	2321.3168	2321.3147	0.91	1	29	0.0013	1	U	K.KVQTVSKPSPFLFQITFNLK.N
9463	874 - 893	774.7797	2321.3172	2321.3147	1.04	1	69	1.4e-007	1	U	K.KVQTVSKPSPFLFQITFNLK.N
9464	874 - 893	1161.6676	2321.3207	2321.3147	2.55	1	24	0.0043	1	U	K.KVQTVSKPSPFLFQITFNLK.N
14601	874 - 903	838.7042	3350.7875	3350.8027	-4.52	2	35	0.0003	1	U	K.KVQTVSKPSPFLFQITFNLKNPNGGTYPTK.D
8955	875 - 893	1097.6147	2193.2149	2193.2198	-2.21	0	44	4e-005	1	U	K.VQTVSKPSPFLFQITFNLK.N
8956	875 - 893	732.0794	2193.2164	2193.2198	-1.55	0	23	0.0049	1	U	K.VQTVSKPSPFLFQITFNLK.N
8959	875 - 893	732.0804	2193.2195	2193.2198	-0.13	0	46	2.5e-005	1	U	K.VQTVSKPSPFLFQITFNLK.N
8960	875 - 893	549.3122	2193.2197	2193.2198	-0.051	0	28	0.0015	1	U	K.VQTVSKPSPFLFQITFNLK.N
8961	875 - 893	732.0805	2193.2197	2193.2198	-0.046	0	5	0.32	1	U	K.VQTVSKPSPFLFQITFNLK.N
8962	875 - 893	732.0807	2193.2202	2193.2198	0.20	0	5	0.34	1	U	K.VQTVSKPSPFLFQITFNLK.N
8963	875 - 893	732.0807	2193.2204	2193.2198	0.29	0	2	0.64	1	U	K.VQTVSKPSPFLFQITFNLK.N
8965	875 - 893	732.0812	2193.2217	2193.2198	0.87	0	5	0.31	1	U	K.VQTVSKPSPFLFQITFNLK.N
8966	875 - 893	732.0812	2193.2217	2193.2198	0.87	0	27	0.0022	1	U	K.VQTVSKPSPFLFQITFNLK.N
8970	875 - 893	732.0817	2193.2232	2193.2198	1.54	0	4	0.41	1	U	K.VQTVSKPSPFLFQITFNLK.N
8971	875 - 893	732.0817	2193.2233	2193.2198	1.62	0	21	0.0075	1	U	K.VQTVSKPSPFLFQITFNLK.N
8973	875 - 893	732.0820	2193.2243	2193.2198	2.04	0	3	0.45	1	U	K.VQTVSKPSPFLFQITFNLK.N
8974	875 - 893	732.0825	2193.2255	2193.2198	2.63	0	3	0.51	1	U	K.VQTVSKPSPFLFQITFNLK.N
8975	875 - 893	732.0827	2193.2263	2193.2198	2.96	0	14	0.041	1	U	K.VQTVSKPSPFLFQITFNLK.N
13798	875 - 903	806.6862	3222.7155	3222.7077	2.41	1	58	1.5e-006	1	U	K.VQTVSKPSPFLFQITFNLKNPNGGTYPTK.D
2245	894 - 903	524.7568	1047.4990	1047.4985	0.46	0	51	8.8e-006	1	U	K.NPNGGTYPTK.D
2246	894 - 903	524.7569	1047.4992	1047.4985	0.69	0	32	0.00064	1	U	K.NPNGGTYPTK.D
9715	894 - 916	1181.5864	2361.1583	2361.1601	-0.76	1	37	0.00021	1	U	K.NPNGGTYPTKDASVELWATVGGK.D
9716	894 - 916	788.0605	2361.1598	2361.1601	-0.12	1	56	2.4e-006	1	U	K.NPNGGTYPTKDASVELWATVGGK.D
3979	904 - 916	444.8959	1331.6658	1331.6721	-4.76	0	50	9.1e-006	1	U	K.DASVELWATVGGK.D
3980	904 - 916	666.8423	1331.6701	1331.6721	-1.50	0	71	8e-008	1	U	K.DASVELWATVGGK.D
3981	904 - 916	666.8431	1331.6717	1331.6721	-0.30	0	71	8.6e-008	1	U	K.DASVELWATVGGK.D
3982	904 - 916	666.8431	1331.6717	1331.6721	-0.30	0	65	3.1e-007	1	U	K.DASVELWATVGGK.D
3983	904 - 916	666.8434	1331.6722	1331.6721	0.062	0	72	5.7e-008	1	U	K.DASVELWATVGGK.D
3984	904 - 916	666.8434	1331.6723	1331.6721	0.15	0	67	2.1e-007	1	U	K.DASVELWATVGGK.D
3985	904 - 916	666.8434	1331.6723	1331.6721	0.15	0	67	2.2e-007	1	U	K.DASVELWATVGGK.D
3986	904 - 916	666.8435	1331.6725	1331.6721	0.24	0	54	4.2e-006	1	U	K.DASVELWATVGGK.D
3987	904 - 916	666.8435	1331.6725	1331.6721	0.24	0	75	3.5e-008	1	U	K.DASVELWATVGGK.D
3988	904 - 916	666.8436	1331.6726	1331.6721	0.34	0	65	3e-007	1	U	K.DASVELWATVGGK.D
3989	904 - 916	666.8436	1331.6726	1331.6721	0.34	0	67	2e-007	1	U	K.DASVELWATVGGK.D
3990	904 - 916	666.8436	1331.6727	1331.6721	0.43	0	75	3.5e-008	1	U	K.DASVELWATVGGK.D
3991	904 - 916	666.8436	1331.6727	1331.6721	0.43	0	68	1.7e-007	1	U	K.DASVELWATVGGK.D
3992	904 - 916	444.8982	1331.6728	1331.6721	0.53	0	49	1.2e-005	1	U	K.DASVELWATVGGK.D
3993	904 - 916	666.8438	1331.6729	1331.6721	0.61	0	49	1.2e-005	1	U	K.DASVELWATVGGK.D
3994	904 - 916	666.8438	1331.6729	1331.6721	0.61	0	67	2.2e-007	1	U	K.DASVELWATVGGK.D
3995	904 - 916	666.8438	1331.6731	1331.6721	0.70	0	72	6e-008	1	U	K.DASVELWATVGGK.D
3996	904 - 916	666.8438	1331.6731	1331.6721	0.70	0	70	1e-007	1	U	K.DASVELWATVGGK.D
3997	904 - 916	666.8439	1331.6732	1331.6721	0.79	0	55	3.4e-006	1	U	K.DASVELWATVGGK.D
3998	904 - 916	666.8439	1331.6732	1331.6721	0.79	0	58	1.5e-006	1	U	K.DASVELWATVGGK.D
3999	904 - 916	666.8439	1331.6732	1331.6721	0.79	0	62	5.9e-007	1	U	K.DASVELWATVGGK.D
4000	904 - 916	666.8439	1331.6733	1331.6721	0.89	0	69	1.1e-007	1	U	K.DASVELWATVGGK.D
4001	904 - 916	666.8440	1331.6734	1331.6721	0.98	0	68	1.7e-007	1	U	K.DASVELWATVGGK.D
4002	904 - 916	666.8441	1331.6736	1331.6721	1.07	0	62	6.3e-007	1	U	K.DASVELWATVGGK.D
4003	904 - 916	666.8445	1331.6745	1331.6721	1.80	0	73	5.2e-008	1	U	K.DASVELWATVGGK.D
4004	904 - 916	666.8464	1331.6783	1331.6721	4.65	0	44	3.7e-005	1	U	K.DASVELWATVGGK.D
5711	904 - 919	563.6316	1687.8729	1687.8781	-3.05	1	19	0.014	1	U	K.DASVELWATVGGKDLK.V
5712	904 - 919	844.9470	1687.8794	1687.8781	0.75	1	29	0.0012	1	U	K.DASVELWATVGGKDLK.V
47	920 - 923	248.6656	495.3167	495.3169	-0.39	0	25	0.003	1	U	K.VLHK.W

Query	Start - End	Observed	Mr(expt)	Mr(calc)	ppm	M	Score	Expect	Rank	U	Peptide
150	924 - 927	287.6710	573.3274	573.3275	-0.18	0	9	0.12	1	U	K.WIQK.S
152	924 - 927	287.6710	573.3275	573.3275	0.037	0	7	0.19	1	U	K.WIQK.S
153	924 - 927	287.6711	573.3277	573.3275	0.46	0	13	0.049	1	U	K.WIQK.S
154	924 - 927	287.6712	573.3278	573.3275	0.57	0	6	0.27	1	U	K.WIQK.S
155	924 - 927	287.6712	573.3278	573.3275	0.57	0	13	0.046	1	U	K.WIQK.S
156	924 - 927	287.6712	573.3279	573.3275	0.68	0	5	0.34	1	U	K.WIQK.S
5880	924 - 937	865.3953	1728.7760	1728.7777	-1.01	1	41	7.8e-005	1	U	K.WIQKSDVMYSQTNN.- + Oxidation (M)
2843	928 - 937	579.7413	1157.4680	1157.4659	1.81	0	54	3.6e-006	1	U	K.SDVMYSQTNN.-
2934	928 - 937	587.7374	1173.4602	1173.4608	-0.54	0	44	4.1e-005	1	U	K.SDVMYSQTNN.- + Oxidation (M)
2935	928 - 937	587.7376	1173.4607	1173.4608	-0.13	0	32	0.00061	1	U	K.SDVMYSQTNN.- + Oxidation (M)
2936	928 - 937	587.7377	1173.4609	1173.4608	0.083	0	37	0.00022	1	U	K.SDVMYSQTNN.- + Oxidation (M)
2937	928 - 937	587.7380	1173.4614	1173.4608	0.50	0	46	2.7e-005	1	U	K.SDVMYSQTNN.- + Oxidation (M)
2938	928 - 937	587.7383	1173.4620	1173.4608	1.02	0	61	8.6e-007	1	U	K.SDVMYSQTNN.- + Oxidation (M)
2939	928 - 937	587.7383	1173.4620	1173.4608	1.02	0	20	0.011	1	U	K.SDVMYSQTNN.- + Oxidation (M)

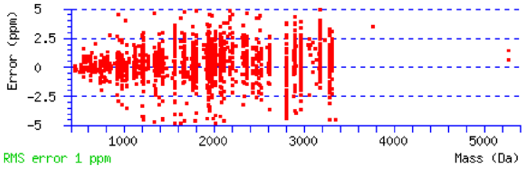

Mascot: http://www.matrixscience.com/
