# Supplementary material for: Food and feed safety of the Bacillus thuringiensis derived protein Vpb4Da2, a novel protein for control of western corn rootworm
Source: PLoS One. 2022 Aug 3;17(8):e0272311. doi: 10.1371/journal.pone.0272311 (PMC9348738; doi:10.1371/journal.pone.0272311)
Supplement: S2 File — (PDF) [file pone.0272311.s003.pdf]

Insect bioassay for protein characterization

	BioAssay ID	Treatment	concentration (µg/mL diet)	Number of test insects	Number of insects survived	Combined mass (mg)	Mean insect mass (mg)	EC50	95% CI
Assay repeat #1	210630.WCR.Vpb4.Activity.R2	BlankControl-R1	0	36	32	16.3	0.51		
	210630.WCR.Vpb4.Activity.R2	BlankControl-R2	0	35	31	20.0	0.65		
	210630.WCR.Vpb4.Activity.R2	BlankControl-R3	0	35	31	17.5	0.56		
	210630.WCR.Vpb4.Activity.R2	Plant-produced Vpb4Da	0.5	35	33	16.7	0.51	14	9.5 - 20
	210630.WCR.Vpb4.Activity.R2	Plant-produced Vpb4Da	1	36	31	15.1	0.49		
	210630.WCR.Vpb4.Activity.R2	Plant-produced Vpb4Da	2	36	34	17.4	0.51		
	210630.WCR.Vpb4.Activity.R2	Plant-produced Vpb4Da	4	36	32	14.0	0.44		
	210630.WCR.Vpb4.Activity.R2	Plant-produced Vpb4Da	8	36	30	12.1	0.40		
	210630.WCR.Vpb4.Activity.R2	Plant-produced Vpb4Da	16	36	31	7.2	0.23		
	210630.WCR.Vpb4.Activity.R2	Plant-produced Vpb4Da	32	35	21	3.2	0.15		
	210630.WCR.Vpb4.Activity.R2	E.coli-produced Vpb4Da	0.5	36	28	16.6	0.59	5.6	4.0 - 7.9
	210630.WCR.Vpb4.Activity.R2	E.coli-produced Vpb4Da	1	36	32	17.0	0.53		
	210630.WCR.Vpb4.Activity.R2	E.coli-produced Vpb4Da	2	36	29	14.2	0.49		
	210630.WCR.Vpb4.Activity.R2	E.coli-produced Vpb4Da	4	36	25	8.9	0.36		
	210630.WCR.Vpb4.Activity.R2	E.coli-produced Vpb4Da	8	36	17	3.1	0.18		
	210630.WCR.Vpb4.Activity.R2	E.coli-produced Vpb4Da	16	34	12	1.3	0.11		
	210630.WCR.Vpb4.Activity.R2	E.coli-produced Vpb4Da	32	34	3	0.4	0.13		
Assay repeat #2	210708.WCR.Vpb4.Activity.R3	BlankControl-R1	0	36	28	13.6	0.49		
	210708.WCR.Vpb4.Activity.R3	BlankControl-R2	0	36	26	13.5	0.52		
	210708.WCR.Vpb4.Activity.R3	BlankControl-R3	0	36	26	13.0	0.50		
	210708.WCR.Vpb4.Activity.R3	Plant-produced Vpb4Da	0.5	37	33	16.1	0.49	11	9.0- 12
	210708.WCR.Vpb4.Activity.R3	Plant-produced Vpb4Da	1	35	28	12.4	0.44		
	210708.WCR.Vpb4.Activity.R3	Plant-produced Vpb4Da	2	36	32	13.5	0.42		
	210708.WCR.Vpb4.Activity.R3	Plant-produced Vpb4Da	4	36	30	10.7	0.36		
	210708.WCR.Vpb4.Activity.R3	Plant-produced Vpb4Da	8	36	26	6.8	0.26		
	210708.WCR.Vpb4.Activity.R3	Plant-produced Vpb4Da	16	37	31	6.6	0.21		
	210708.WCR.Vpb4.Activity.R3	Plant-produced Vpb4Da	32	36	17	2.5	0.15		
	210708.WCR.Vpb4.Activity.R3	E.coli-produced Vpb4Da	0.5	37	25	12.6	0.50	6.4	5.4 - 7.6
	210708.WCR.Vpb4.Activity.R3	E.coli-produced Vpb4Da	1	36	24	10.5	0.44		
	210708.WCR.Vpb4.Activity.R3	E.coli-produced Vpb4Da	2	36	30	12.4	0.41		
	210708.WCR.Vpb4.Activity.R3	E.coli-produced Vpb4Da	4	37	30	10.8	0.36		
	210708.WCR.Vpb4.Activity.R3	E.coli-produced Vpb4Da	8	39	31	5.8	0.19		
	210708.WCR.Vpb4.Activity.R3	E.coli-produced Vpb4Da	16	37	13	1.4	0.11		
	210708.WCR.Vpb4.Activity.R3	E.coli-produced Vpb4Da	32	35	3	0.2	0.07		
Assay repeat #3	210709.WCR.Vpb4.Activity.R4	BlankControl-R1	0	37	35	14.7	0.42		
	210709.WCR.Vpb4.Activity.R4	BlankControl-R2	0	36	33	18.0	0.55		
	210709.WCR.Vpb4.Activity.R4	BlankControl-R3	0	35	32	18.6	0.58		
	210709.WCR.Vpb4.Activity.R4	Plant-produced Vpb4Da	0.5	37	34	18.6	0.55	12	8.0 - 18
	210709.WCR.Vpb4.Activity.R4	Plant-produced Vpb4Da	1	37	31	15.3	0.49		
	210709.WCR.Vpb4.Activity.R4	Plant-produced Vpb4Da	2	37	34	16.5	0.49		
	210709.WCR.Vpb4.Activity.R4	Plant-produced Vpb4Da	4	37	31	13.3	0.43		
	210709.WCR.Vpb4.Activity.R4	Plant-produced Vpb4Da	8	36	24	7.4	0.31		
	210709.WCR.Vpb4.Activity.R4	Plant-produced Vpb4Da	16	36	25	4.9	0.20		
	210709.WCR.Vpb4.Activity.R4	Plant-produced Vpb4Da	32	32	18	2.8	0.16		
	210709.WCR.Vpb4.Activity.R4	E.coli-produced Vpb4Da	0.5	32	25	18.1	0.72	6.3	3.4 - 12
	210709.WCR.Vpb4.Activity.R4	E.coli-produced Vpb4Da	1	36	35	20.9	0.60		
	210709.WCR.Vpb4.Activity.R4	E.coli-produced Vpb4Da	2	32	30	16.7	0.56		
	210709.WCR.Vpb4.Activity.R4	E.coli-produced Vpb4Da	4	37	27	9	0.33		
	210709.WCR.Vpb4.Activity.R4	E.coli-produced Vpb4Da	8	36	30	5.9	0.20		
	210709.WCR.Vpb4.Activity.R4	E.coli-produced Vpb4Da	16	37	15	2	0.13		
	210709.WCR.Vpb4.Activity.R4	E.coli-produced Vpb4Da	32	37	4	0.6	0.15		

Insect bioassay for controls and heat treated samples

BioAssay ID	Treatment	concentration (µg/mL diet)	Number of test insects	Number of insects survived	Combined mass (mg)	Mean insect mass (mg)	EC50	95% CI
201007.WCR.Vpb4Da2.Heat.R1	BlankControl_Vpb4	0	33	32	9.7	0.30		
201007.WCR.Vpb4Da2.Heat.R1	BlankControl_Vpb4	0	35	34	10.1	0.30		
201007.WCR.Vpb4Da2.Heat.R1	BlankControl_Vpb4	0	30	28	7.6	0.27		
201007.WCR.Vpb4Da2.Heat.R1	Vpb4Da2 Ice	0.5	29	27	7.4	0.27		
201007.WCR.Vpb4Da2.Heat.R1	Vpb4Da2 Ice	1	33	32	9.9	0.31		
201007.WCR.Vpb4Da2.Heat.R1	Vpb4Da2 Ice	2	33	33	8.6	0.26		
201007.WCR.Vpb4Da2.Heat.R1	Vpb4Da2 Ice	4	29	28	7.2	0.26	10	7.4 - 14
201007.WCR.Vpb4Da2.Heat.R1	Vpb4Da2 Ice	8	28	26	3.5	0.13		
201007.WCR.Vpb4Da2.Heat.R1	Vpb4Da2 Ice	16	30	23	3.0	0.13		
201007.WCR.Vpb4Da2.Heat.R1	Vpb4Da2 Ice	32	24	6	0.2	0.03		
201007.WCR.Vpb4Da2.Heat.R1	Vpb4Da2 15 @ 25	0.5	31	31	10.0	0.32		
201007.WCR.Vpb4Da2.Heat.R1	Vpb4Da2 15 @ 25	1	26	26	7.2	0.28		
201007.WCR.Vpb4Da2.Heat.R1	Vpb4Da2 15 @ 25	2	25	24	7.2	0.30		
201007.WCR.Vpb4Da2.Heat.R1	Vpb4Da2 15 @ 25	4	31	29	7.6	0.26	14	11 -1 8
201007.WCR.Vpb4Da2.Heat.R1	Vpb4Da2 15 @ 25	8	27	22	4.2	0.19		
201007.WCR.Vpb4Da2.Heat.R1	Vpb4Da2 15 @ 25	16	30	16	2.1	0.13		
201007.WCR.Vpb4Da2.Heat.R1	Vpb4Da2 15 @ 25	32	24	11	1.0	0.09		
201007.WCR.Vpb4Da2.Heat.R1	Vpb4Da2 15 @ 37	0.5	25	21	8.3	0.40		
201007.WCR.Vpb4Da2.Heat.R1	Vpb4Da2 15 @ 37	1	27	22	6.3	0.29		
201007.WCR.Vpb4Da2.Heat.R1	Vpb4Da2 15 @ 37	2	28	27	9.2	0.34		
201007.WCR.Vpb4Da2.Heat.R1	Vpb4Da2 15 @ 37	4	26	22	5.8	0.26	14	8.0 - 26
201007.WCR.Vpb4Da2.Heat.R1	Vpb4Da2 15 @ 37	8	24	21	4.2	0.20		
201007.WCR.Vpb4Da2.Heat.R1	Vpb4Da2 15 @ 37	16	21	13	1.6	0.12		
201007.WCR.Vpb4Da2.Heat.R1	Vpb4Da2 15 @ 37	32	27	5	0.6	0.12		
201007.WCR.Vpb4Da2.Heat.R1	Vpb4Da2 15 @ 55	0.5	16	16	5.0	0.31		
201007.WCR.Vpb4Da2.Heat.R1	Vpb4Da2 15 @ 55	1	27	25	9.5	0.38		
201007.WCR.Vpb4Da2.Heat.R1	Vpb4Da2 15 @ 55	2	26	24	8.9	0.37		
201007.WCR.Vpb4Da2.Heat.R1	Vpb4Da2 15 @ 55	4	29	29	10.4	0.36	NA	NA
201007.WCR.Vpb4Da2.Heat.R1	Vpb4Da2 15 @ 55	8	29	27	10.9	0.40		
201007.WCR.Vpb4Da2.Heat.R1	Vpb4Da2 15 @ 55	16	31	30	11.1	0.37		
201007.WCR.Vpb4Da2.Heat.R1	Vpb4Da2 15 @ 55	32	26	23	8.8	0.38		
201007.WCR.Vpb4Da2.Heat.R1	Vpb4Da2 15 @ 75	0.5	25	23	7.5	0.33		
201007.WCR.Vpb4Da2.Heat.R1	Vpb4Da2 15 @ 75	1	25	20	7.2	0.36		
201007.WCR.Vpb4Da2.Heat.R1	Vpb4Da2 15 @ 75	2	26	25	8.6	0.34		
201007.WCR.Vpb4Da2.Heat.R1	Vpb4Da2 15 @ 75	4	29	25	8.8	0.35	NA	NA
201007.WCR.Vpb4Da2.Heat.R1	Vpb4Da2 15 @ 75	8	26	23	8.5	0.37		
201007.WCR.Vpb4Da2.Heat.R1	Vpb4Da2 15 @ 75	16	22	18	6.5	0.36		
201007.WCR.Vpb4Da2.Heat.R1	Vpb4Da2 15 @ 75	32	24	19	8.5	0.45		
201007.WCR.Vpb4Da2.Heat.R1	Vpb4Da2 15 @ 95	0.5	28	25	10.0	0.40		
201007.WCR.Vpb4Da2.Heat.R1	Vpb4Da2 15 @ 95	1	20	17	7.8	0.46		
201007.WCR.Vpb4Da2.Heat.R1	Vpb4Da2 15 @ 95	2	27	24	8.8	0.37		
201007.WCR.Vpb4Da2.Heat.R1	Vpb4Da2 15 @ 95	4	30	26	10.5	0.40	NA	NA
201007.WCR.Vpb4Da2.Heat.R1	Vpb4Da2 15 @ 95	8	26	21	7.0	0.33		
201007.WCR.Vpb4Da2.Heat.R1	Vpb4Da2 15 @ 95	16	27	18	6.4	0.36		
201007.WCR.Vpb4Da2.Heat.R1	Vpb4Da2 15 @ 95	32	32	27	10.8	0.40		
201007.WCR.Vpb4Da2.Heat.R1	Vpb4Da2 30 @ 25	0.5	29	25	9.2	0.37		
201007.WCR.Vpb4Da2.Heat.R1	Vpb4Da2 30 @ 25	1	30	27	11.5	0.43		
201007.WCR.Vpb4Da2.Heat.R1	Vpb4Da2 30 @ 25	2	30	28	10.4	0.37		
201007.WCR.Vpb4Da2.Heat.R1	Vpb4Da2 30 @ 25	4	31	25	7.4	0.30	10	6.1 - 16
201007.WCR.Vpb4Da2.Heat.R1	Vpb4Da2 30 @ 25	8	31	25	4.3	0.17		
201007.WCR.Vpb4Da2.Heat.R1	Vpb4Da2 30 @ 25	16	31	15	1.7	0.11		
201007.WCR.Vpb4Da2.Heat.R1	Vpb4Da2 30 @ 25	32	31	2	0.1	0.05		
201007.WCR.Vpb4Da2.Heat.R1	Vpb4Da2 30 @ 37	0.5	31	30	14.4	0.48		
201007.WCR.Vpb4Da2.Heat.R1	Vpb4Da2 30 @ 37	1	33	28	14.3	0.51		
201007.WCR.Vpb4Da2.Heat.R1	Vpb4Da2 30 @ 37	2	32	27	12.8	0.47		
201007.WCR.Vpb4Da2.Heat.R1	Vpb4Da2 30 @ 37	4	31	26	8.5	0.33	12	6.4 - 24
201007.WCR.Vpb4Da2.Heat.R1	Vpb4Da2 30 @ 37	8	30	25	7	0.28		
201007.WCR.Vpb4Da2.Heat.R1	Vpb4Da2 30 @ 37	16	31	17	2.4	0.14		
201007.WCR.Vpb4Da2.Heat.R1	Vpb4Da2 30 @ 37	32	30	3	0.1	0.03		
201007.WCR.Vpb4Da2.Heat.R1	Vpb4Da2 30 @ 55	0.5	30	26	13.3	0.51		
201007.WCR.Vpb4Da2.Heat.R1	Vpb4Da2 30 @ 55	1	30	26	14.3	0.55		
201007.WCR.Vpb4Da2.Heat.R1	Vpb4Da2 30 @ 55	2	30	23	11.6	0.50		
201007.WCR.Vpb4Da2.Heat.R1	Vpb4Da2 30 @ 55	4	30	27	15.1	0.56	NA	NA
201007.WCR.Vpb4Da2.Heat.R1	Vpb4Da2 30 @ 55	8	30	23	12.3	0.53		
201007.WCR.Vpb4Da2.Heat.R1	Vpb4Da2 30 @ 55	16	30	26	13.9	0.53		
201007.WCR.Vpb4Da2.Heat.R1	Vpb4Da2 30 @ 55	32	29	22	10.9	0.50		
201007.WCR.Vpb4Da2.Heat.R1	Vpb4Da2 30 @ 75	0.5	24	20	10.2	0.51		
201007.WCR.Vpb4Da2.Heat.R1	Vpb4Da2 30 @ 75	1	30	26	14	0.54		
201007.WCR.Vpb4Da2.Heat.R1	Vpb4Da2 30 @ 75	2	23	21	8.3	0.40		
201007.WCR.Vpb4Da2.Heat.R1	Vpb4Da2 30 @ 75	4	25	22	8.4	0.38	NA	NA
201007.WCR.Vpb4Da2.Heat.R1	Vpb4Da2 30 @ 75	8	25	20	8.2	0.41		
201007.WCR.Vpb4Da2.Heat.R1	Vpb4Da2 30 @ 75	16	23	19	6.7	0.35		
201007.WCR.Vpb4Da2.Heat.R1	Vpb4Da2 30 @ 75	32	18	12	5.5	0.46		
201007.WCR.Vpb4Da2.Heat.R1	Vpb4Da2 30 @ 95	0.5	29	25	12.95	0.52		
201007.WCR.Vpb4Da2.Heat.R1	Vpb4Da2 30 @ 95	1	26	23	12.44	0.54		
201007.WCR.Vpb4Da2.Heat.R1	Vpb4Da2 30 @ 95	2	28	26	16.78	0.65		
201007.WCR.Vpb4Da2.Heat.R1	Vpb4Da2 30 @ 95	4	28	26	11.35	0.44	NA	NA
201007.WCR.Vpb4Da2.Heat.R1	Vpb4Da2 30 @ 95	8	29	26	16.91	0.65		
201007.WCR.Vpb4Da2.Heat.R1	Vpb4Da2 30 @ 95	16	24	19	9.33	0.49		
201007.WCR.Vpb4Da2.Heat.R1	Vpb4Da2 30 @ 95	32	25	17	9.33	0.55		
